# Aberrant CD8^+^T cells drive reproductive dysfunction in female mice with elevated IFN-γ levels

**DOI:** 10.3389/fimmu.2024.1368572

**Published:** 2024-04-18

**Authors:** Enitome E. Bafor, Rebecca A. Erwin-Cohen, Toni Martin, Clayton Baker, Adrienne E. Kimmel, Olivier Duverger, John M. Fenimore, Meredith Ramba, Thea Spindel, Megan M. Hess, Michael Sanford, Vanja Lazarevic, Bérénice A. Benayoun, Howard A. Young, Julio C. Valencia

**Affiliations:** ^1^ Cancer Innovation Laboratory, Center for Cancer Research, National Cancer Institute, National Institutes of Health, Frederick, MD, United States; ^2^ Leonard Davis School of Gerontology, University of Southern California, Los Angeles, CA, United States; ^3^ Molecular and Computational Biology Department, University of Southern California, Dornsife College of Letters, Arts and Sciences, Los Angeles, CA, United States; ^4^ Craniofacial Anomalies and Regeneration Section, National Institute of Dental and Craniofacial Research, National Institutes of Health, Bethesda, MD, United States; ^5^ Experimental Immunology Branch, Center for Cancer Research, National Cancer Institute, Bethesda, MD, United States

**Keywords:** interferon-gamma (IFN-γ), CD8^+^ T cells, hypophysitis, tissue-resident memory, luteinization defect, prolactin deficiency, implantation failure, pregnancy

## Abstract

**Introduction:**

Interferon-gamma (IFN-γ) is pivotal in orchestrating immune responses during healthy pregnancy. However, its dysregulation, often due to autoimmunity, infections, or chronic inflammatory conditions, is implicated in adverse reproductive outcomes such as pregnancy failure or infertility. Additionally, the underlying immunological mechanisms remain elusive.

**Methods:**

Here, we explore the impact of systemic IFN-γ elevation on cytotoxic T cell responses in female reproduction utilizing a systemic lupus-prone mouse model with impaired IFN-γ degradation.

**Results:**

Our findings reveal that heightened IFN-γ levels triggered the infiltration of CD8^+^T cells in the pituitary gland and female reproductive tract (FRT), resulting in prolactin deficiency and subsequent infertility. Furthermore, we demonstrate that chronic IFN-γ elevation increases effector memory CD8^+^T cells in the murine ovary and uterus.

**Discussion:**

These insights broaden our understanding of the role of elevated IFN-γ in female reproductive dysfunction and suggest CD8^+^T cells as potential immunotherapeutic targets in female reproductive disorders associated with chronic systemic IFN-γ elevation.

## Introduction

Immunological imbalance in the female reproductive tract (FRT) contributes to high rates of implantation failure in women (1) and rodents (2–5). However, there is poor understanding of the contribution of immune activation to upstream signals that affect embryo implantation in systemic elevated interferon-gamma (IFN-γ) signaling, which occurs in autoimmunity and chronic inflammatory diseases.

A successful pregnancy requires tight coordination between immune cells and IFN-γ signaling (6–8). Reports indicate a central role for cytokines in mediating a successful pregnancy from implantation [blastocyst adhesion to uterine epithelium (1)] to delivery (7, 9, 10). Specifically, implantation is one of the critical but vulnerable processes for pregnancy success (11), accompanied by an inflammatory response involving IFN-γ (12, 13). However, conditions that induce inappropriate IFN-γ expression, such as infections (14), chronic metabolic imbalance (15), immune checkpoint inhibitor therapy (16, 17), autoimmunity (18), and chronic inflammatory diseases, cause aberrant immune activation and increase the risk of reproductive failure in women (19, 20). Thus, though IFN-γ participates in physiological reproductive processes, when overexpressed, implantation will be compromised (20, 21). Furthermore, systemic immune contribution and specifically the cytotoxic CD8^+^T cell contribution to peri-implantation defects in the context of elevated systemic IFN-γ remain largely unclear.

Infections can cause a break in immune tolerance, leading to pregnancy failure (22). In mouse models of *Listeria monocytogenes*, infection is sufficient to recruit fetal-specific CD8^+^ T cells to the placenta and cause fetal death (5). Moreover, in mouse models of malaria infection, Toll-like receptor 4 (TLR4) and IFN-γ signaling mediate pregnancy complications and failure (23, 24). In addition, elevated IFN-γ mediates fetal resorption after *Toxoplasma* infection in mice (25). Consistent with these findings, women with malaria have increased circulating IFN-γ (13.63–20.29 pg/mL) (26), which correlates with poor pregnancy outcomes (27). Women with placental malaria also have increased effector memory CD8^+^T cells (T_EM_) *in utero* (28). In addition to infection, metabolic diseases (such as obesity and diabetes) lead to a state of chronic low-grade inflammation and immune activation, which contribute to an increased risk of pregnancy complications (29, 30). Maternal autoimmune diseases also contribute to pregnancy complications (6), and since several autoimmune diseases affect women of childbearing age, the effect of these diseases on pregnancy is of high relevance. One commonly recognized autoimmune disease associated with pregnancy complications is systemic lupus erythematosus (SLE), characterized by elevated IFN-γ and systemic inflammation (6). Women with SLE have an increased risk of preeclampsia, preterm delivery, fetal growth restriction, and fetal loss (31), and the cellular immunological mechanisms contributing to systemic elevated IFN-γ disruption of fertility in these conditions are still largely unknown.

Importantly, endocrine support for fertility is also disrupted during chronic inflammation. Autoimmunity and chronic inflammation often involve an interaction between the endocrine and immune systems (32). Since the hypothalamic–pituitary axis closely regulates most endocrine organs, disruption of pituitary function affects the FRT. Prolactin (Prl), synthesized and released mainly from the anterior pituitary, is a commonly dysregulated hormone during inflammation (33). Prl is critical for reproduction and regulates multiple reproductive functions such as oocyte development, the formation, function, and survival of corpora lutea (CL), embryo implantation, and lactation (though differences exist between women and rodents), and Prl knockout mice are completely infertile (34–36). In young women, hyper- and hypo-prolactinemia are common endocrine pituitary disorders associated with anovulation, amenorrhea, infertility, and disrupted postpartum lactation in women and murine models (37, 38). Therefore, a threshold of circulating Prl [approximately 3.0–15.0 µg/L in women (38) and approximately ≥20 ng/mL in rodents (39)] appears necessary for optimal reproductive outcomes. Prl levels above or below these critical thresholds result in reproductive dysfunction (38). Hyper- and hypo-prolactinemia frequently occur in autoimmune diseases (40, 41), with hypoprolactinemia largely due to hypophysitis (inflammation of the pituitary gland) (42–45) and inflammatory disorders (46–48). In some cases, hypophysitis presents as an enlarged pituitary with elevated Prl and eventually progresses to hypoprolactinemia (49, 50). Although some differences exist with the role of Prl in reproduction between women and rodents, Prl dysregulation in the presence of elevated IFN-γ in both species supports a role for immune activation in pituitary modulation and requires further investigation.

Mouse models that mimic inflammation and infection have been informative in understanding the mechanisms of disease initiation and progression. In this regard, genetically modified rodent models of inflammation and pregnancy are essential in pre-translational investigations to understand underlying factors responsible for inflammation-associated pregnancy disorders. Several mouse strains, such as MRL/*lpr*, (NZB × NZW)F1 (NZB/W), and NZM2410, develop spontaneous lupus-like diseases (51). However, except when induced, the female mice of these lupus models do not develop sufficient clinical reproductive manifestations as seen in human SLE—for instance, MRL-*lpr/lpr* mice display lymphocytic infiltration of the ovarian interstitium, reduced corpora lutea (CL), decreased luteinization, and decreased oocyte pick-up rate (52, 53). However, these mice ovulate and produce an average of seven pups per litter (54), indicating that fertility and pregnancy outcomes are not markedly altered [which is not the case in the majority of SLE patients (55, 56)]. Therefore, these models do not closely reflect the reproductive phenotype in patients with SLE or elevated IFN-γ. Furthermore, to our knowledge, most studies investigating the role of elevated IFN-γ in female reproductive complications (outside infection-induced models) examine the localized effects of varying exogenously administered IFN-γ concentrations, which do not consider the systemic IFN-γ contribution to modulation of cytotoxic T cells upstream of implantation.

Our laboratory developed a mouse model in which the 162 nt region of the AU-rich elements (ARE) sequence in the 3′ untranslated region (3′UTR) of the *Ifng* gene was replaced with random nucleotides (57). Genetic alteration of the *Ifng* gene impaired IFN-γ regulation and resulted in increased stability of *Ifng* mRNA. Consequently, female mice heterozygous (ARE^+/-^) and homozygous (ARE^-/-^) for the ARE replacement showed mild to moderate elevation of circulating IFN-γ levels, respectively, and developed lupus-like symptoms associated with compromised fertility (57). The elevated IFN-γ in the ARE (ARE^+/-^ and ARE^-/-^) mouse model allowed the investigation of the systemic impact of chronically elevated IFN-γ on CD8^+^ T cell modulation in the FRT and pregnancy outcome.

We report here that though ARE^+/-^ and ARE^-/-^ female mice with mild to moderately elevated IFN-γ (respectively) ovulate, develop CL (though reduced), and whose fertilization is not impaired, there is increased CD8^+^T cell infiltration in the ovary and uterus. However, unlike existing lupus mouse models, ARE^-/-^ female mice do not get pregnant or show implantation sites, while ARE^+/-^ female mice have reduced litter sizes, mimicking infertility phenotypes found in SLE and most autoimmune patients (55, 58, 59). Furthermore, we report that aberrant CD8^+^T cells in the ARE^+/-^ and ARE^-/-^ mouse ovary and uterus acquire effector memory signatures capable of propagating self-tissue damage. The persistently elevated IFN-γ during the peri-implantation period of the ARE^-/-^ mice led to breeding difficulties that severely limited the number of animals available for this study. However, our findings support a critical role for elevated IFN-γ in female reproductive abnormalities and highlight cellular mechanisms involving CD8^+^T cells. Thus, data from this study are relevant for clinical findings reporting elevated systemic IFN-γ as a contributor to pregnancy and embryo implantation failure.

## Results

### ARE^-/-^ mice ovulate but do not get pregnant

**Figure 1:**
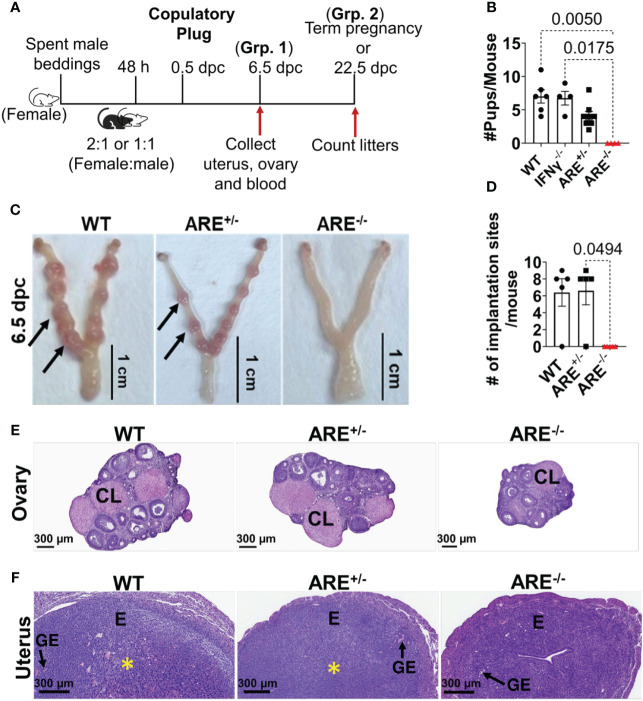
ARE^-/-^ female mice are infertile. **(A)** Schematic depicting the pairing and timing of tissue collection for fertility outcome experiments. dpc, days post-coitum; Grp, Group. **(B)** The average number of pups delivered per litter per group (*n* = 7–8). **(C)** Representative whole-mount 6.5 dpc uteri showing the implantation sites (black arrows) of pregnant wild-type (WT) and ARE^+/-^ female mice. ARE^-/-^ mice that were plugged but not pregnant displayed no implantation sites at 6.5 dpc, demonstrating complete implantation failure. **(D)** ARE^+/-^ female mice show comparable implantation sites at 6.5 dpc with WT controls (*n* = 4–5). Representative WT, ARE^+/-^, and ARE^-/-^ mice histology sections at 6.5 dpc of the **(E)** ovary and **(F)** uterus (*n* = 4–5). CL, corpus luteum; E, endometrium; GE, glandular epithelium. The yellow asterisk (*) indicates decidualization. All experiments were performed two independent times. Statistical significance: one-way ANOVA with Kruskal–Wallis test was performed in all cases. Data represent mean ± SEM, and *n* denotes animals per group.

Female ARE^-/-^ mice are infertile (57). Therefore, to investigate the reproductive pathophysiology in ARE^-/-^ mice, the impact of the chronic IFN-γ environment on ovulation occurrence and fertility was examined. We paired female mice with fertile male mice in a 2:1 or 1:1 female–male ratio (**Figure 1A**). Following pairing, the female mice were examined daily (every morning) for evidence of mating, indicated by copulatory plugs. The day copulatory plugs were seen was noted as 0.5 days post-coitum (dpc). Mice with copulatory plugs were either euthanized at 6.5 dpc (group 1) or allowed to proceed to term (group 2) (**Figure 1A**). ARE^-/-^ mice had a plugging efficiency of 54.55%, which was lower than that of ARE^+/-^, IFN-γ^-/-^, and WT mice, which showed plugging efficiencies of 78.57%, 80.00%, and 85.71%, respectively (**Supplementary Figure S1A**). Despite a lower plugging efficiency, our findings suggest that approximately half of the ARE^-/-^ mice demonstrated normal mating behavior and the ability to ovulate. Among mice in group 2, only the WT, IFN-γ^-/-^, and ARE^+/-^ mice delivered pups, while the ARE^-/-^ mice did not (**Figure 1B**). The ARE^+/-^ litter size (number of pups) was notably smaller on average compared to IFN-γ^-/-^ and WT cohorts (**Figure 1B**), though not statistically different, while the plugged ARE^-/-^ mice showed no signs of pregnancy and did not deliver (**Figure 1B**). Furthermore, at 6.5 dpc (group 1), ARE^+/-^ and WT mice showed comparable implantation sites (visible swellings along each uterine horn) [**Figure 1C** (black arrows) and **Figure 1D**]. However, the ARE^-/-^ mouse uterus showed no implantations (**Figures 1C, D**), which explained the absence of pups and pregnancy.

We then examined the histomorphology of the ovaries and uteri from 6.5 dpc WT, ARE^+/-^, and ARE^-/-^ mice. CL were observed in all ovaries, indicating that ovulation occurred in the ARE^-/-^ mice (**Figure 1E**). The 6.5 dpc ARE^-/-^ mouse uteri appeared normal with the presence of endometrial glands (glandular epithelium) but showed no decidualization (yellow asterisk) (**Figure 1F**). In addition, the expression of the uterine gland-specific marker, Forkhead box protein A2 (Foxa2) (60), appeared normal in all groups (**Supplementary Figure S1B**).

Our findings indicate that ARE^-/-^ mice with moderately elevated IFN-γ ovulate but do not get pregnant.

### CD8^+^T cells infiltrate the ovary and uterus of ARE^-/-^ mice

**Figure 2:**
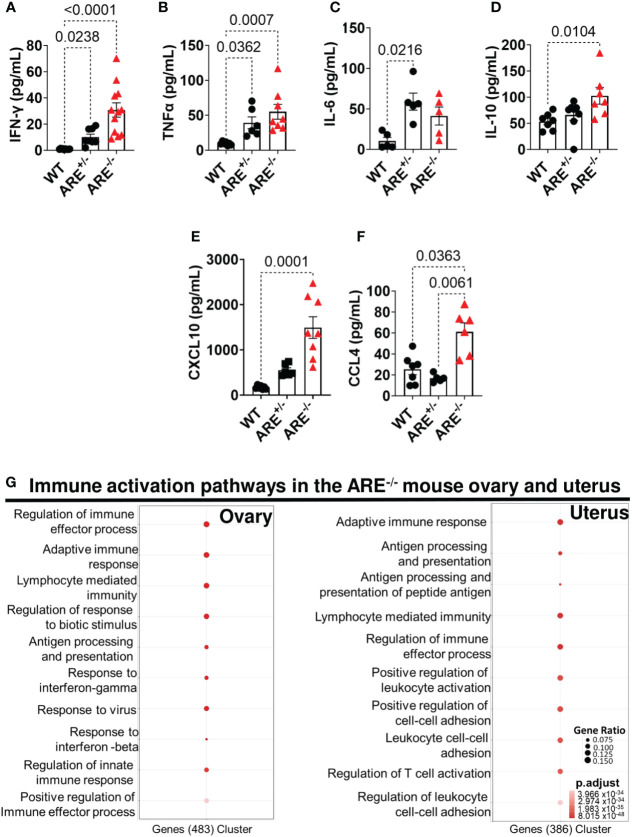
ARE^-/-^ mice displayed elevated immune activation profiles. Circulating plasma levels of **(A)** IFN-γ (*n* = 8–12), **(B)** TNF-α (*n* = 6–8), **(C)** IL-6 (*n* = 5), **(D)** IL-10 (*n* = 7–8), **(E)** CXCL10 (*n* = 6–8), and **(F)** CCL4 (*n* = 5–7) in non-pregnant wild-type (WT) ARE^+/-^ and ARE^-/-^ mice at diestrus. Statistical significance: one-way ANOVA with Kruskal–Wallis test (data from two independent experiments). Data represent mean ± SEM. **(G)** Functional enrichment analysis of the top 10 GO BP terms enriched in genes identified by DESeq2 LRT analysis as being upregulated in non-pregnant ARE^-/-^ mouse ovaries and uteri and downregulated in WT cohorts (*n* = 3, false discovery rate <5%). *n* denotes animals per group in all cases.

Next, we investigated immune profiles across the genotypes. Circulating proinflammatory cytokines, IFN-γ (30.74 ± 5.45 and 10.12 ± 2.16 pg/mL), TNF-α (54.95 ± 10.62 and 39.17 ± 8.37 pg/mL), and IL-6 (41.18 ± 11.09 and 59.00 ± 10.54), were increased in the ARE^-/-^ and ARE^+/-^ mice, respectively, compared to the levels in WT cohorts, including IL-10 (102.40 ± 15.90 and 65.80 ± 10.17 pg/mL) (**Figures 2A–D**). In addition, circulating levels of the proinflammatory chemokines CXCL10 and CCL4 were increased in the ARE^-/-^ mice compared to the ARE^+/-^ and WT cohorts (**Figures 2E, F**) with no difference in IL-1β and CCL2 levels among the groups (**Supplementary Figures S2A, B**). RNA sequencing (RNAseq) identified 3,911 and 2,287 differentially expressed genes (DEGs) (adjusted *p*-value <0.05) between ARE^-/-^ and WT ovarian and uterine tissues, respectively (**Supplementary Figures S2C, D**), with some overlap (**Supplementary Figures S2E, F**). Using the likelihood ratio test (LRT) (false discovery rate (FDR) <1 × 10^-4^), we observed a gene expression pattern of 147 and 37 upregulated genes in WT ovaries and uteri which were downregulated in ARE^-/-^ mice (**Supplementary Figures S3A, B**) and another pattern with 568 and 463 upregulated genes in ARE^-/-^ mouse ovaries and uteri, respectively, which were downregulated in WT mice (**Supplementary Figures S3C, D**). ARE^+/-^ mice showed intermediate expression levels of both patterns (**Supplementary Figures S2**, **S3**), demonstrating a gene dosage effect (61–63) with IFN-γ. Specifically, RNAseq gene ontology (GO) revealed enrichment of immune activation pathways in the ARE^-/-^ mouse ovary and uterus (**Figure 2G**).

**Figure 3:**
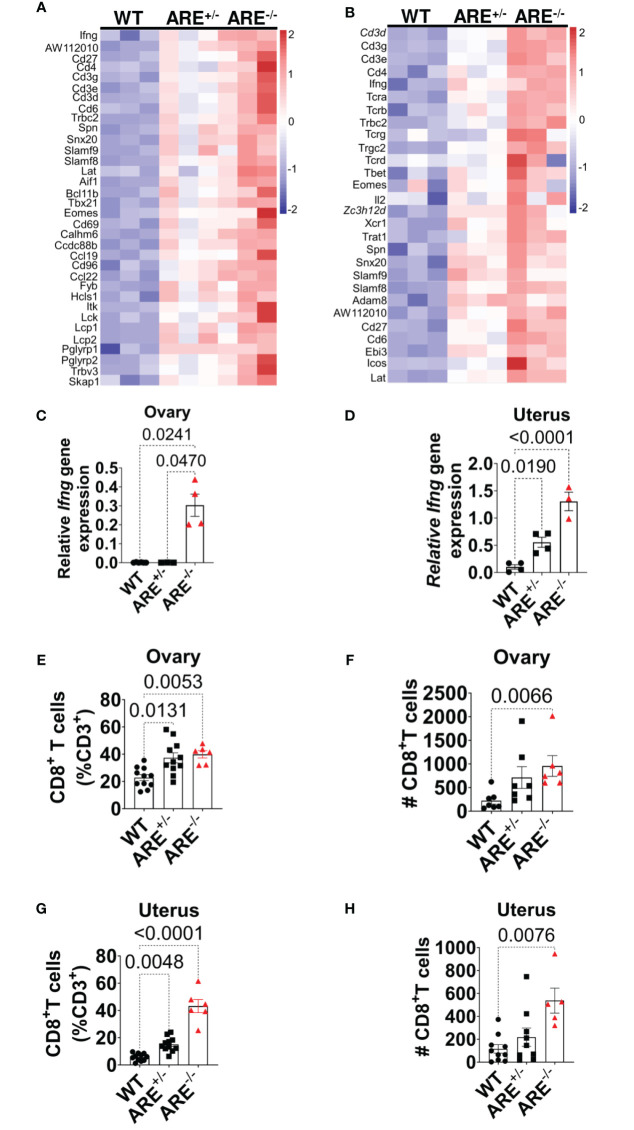
CD8^+^ T cells infiltrated ARE^-/-^ mouse ovary and uterus. **(A, B)** Heat map of normalized gene expression related to T cell activation in non-pregnant ovary and uterus (*n* = 3). **(C, D)** RT-PCR of *Ifng* gene expression relative to *Hprt* in the ovary and uterus (*n* = 4–5). The experiments were performed two independent times (*n* = 3-4). (**E**–**H**) Flow cytometry analysis of the ovary and uterus showing **(E, F)** increased CD8^+^ T cell frequencies and **(G, H)** absolute numbers (*n* = 6–11). The experiments were performed three independent times. Statistical significance: one-way ANOVA with Kruskal–Wallis test was performed in all cases. Data represent mean ± SEM, and *n* denotes animals per group.

**Figure 4:**
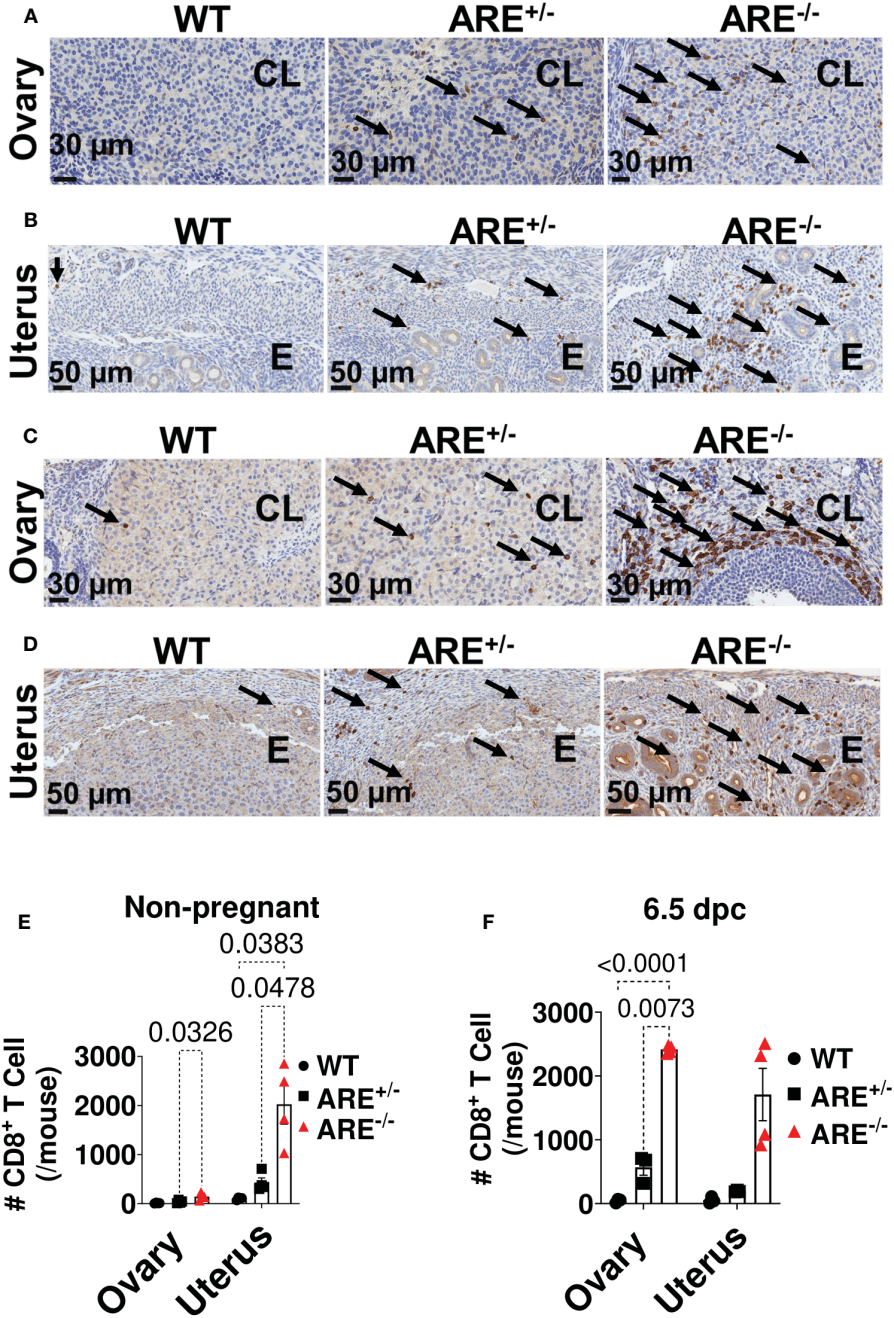
Immunohistochemistry (IHC) supported CD8^+^T cell infiltration of the ARE^-/-^ mouse ovary and uterus. Representative IHC images showing the distribution of CD8a-positive cells (black arrows) in **(A, B)** non-pregnant ovary and uterus and in **(C, D)** 6.5-dpc ovary and uterus. CL, corpus luteum; E, endometrium. Black arrows = CD8a^+^ T cells. **(E, F)** Number of CD8a^+^ T cells within the ovary (two ovary sections per mouse) and uterus (per mm^2^) in **(E)** non-pregnant and **(F)** 6.5-dpc mice (*n* = 4). All experiments were performed two independent times. Statistical significance: two-way ANOVA with Tukey’s multiple-comparison test. Data represent mean ± SEM, and *n* denotes animals per group.

Moreover, genes related to T cell activation were enriched in the ARE^-/-^ mouse ovary and uterus (**Figures 3A, B**, **Supplementary Tables S1**, **S2**), including *Ifng* (**Figures 3A, B**), which was confirmed by RT-PCR (**Figures 3C, D**). Therefore, using flow cytometry analysis and immunohistochemistry (IHC), we investigated the frequency and distribution of cytotoxic cells in the ovary and uterus (the gating strategies are shown in **Supplementary Figure S4**). We found significantly increased CD8^+^ and not CD4^+^ T or NK1.1^+^ cells in the non-pregnant ARE^-/-^ mouse ovary and uterus (**Figures 3E–H** and **Supplementary Figures S5A–D**). In addition, IHC supported the data of increased CD8^+^T cells in the ARE (ARE^+/-^ and ARE^-/-^) mouse ovary and uterus (**Figures 4A–F**).

To determine whether CD8^+^T cell infiltration of the ovary and uterus was an IFN-γ-mediated event, we examined CD8^+^T cell frequencies and infiltration in IFN-γ^-/-^ mouse models. We found no significant difference in CD8^+^T cell frequencies in the IFN-γ^-/-^ mouse ovary and uterus compared to WT cohorts (**Supplementary Figures S6A–F**). These results suggest that the CD8^+^T cell infiltration seen in the ARE^-/-^ mice was an IFN-γ-mediated event. However, given that we did not examine other cytokine null mouse models, further investigations are required to confirm whether IFN-γ mediated these events alone or in association with other pro-inflammatory cytokines.

These findings together suggest a CD8^+^T cell-mediated reproductive dysfunction phenotype in the ARE^-/-^ mouse model.

### CD8^+^T cells in the ARE^-/-^ mouse ovary and uterus do not express hallmark features of exhaustion

In autoimmunity, CD8^+^T cells evade tolerance and exhaustion mechanisms and primarily consist of effector and effector memory such that the capacity to cause damage to self-tissues is retained (64). Therefore, to further investigate the ARE^-/-^ mouse CD8^+^T cell phenotype, we asked whether the CD8^+^T cells within the ARE^-/-^ mouse ovary and uterus displayed effector/effector memory signatures.

First, we evaluated the expression of T cell immunoglobulin and mucin-domain containing-3 (Tim-3) and lymphocyte activation gene 3 (Lag-3), which are expressed on exhausted CD8^+^T cells (65–67). We found that ARE^-/-^ mouse CD8^+^T cells in the ovary or uterus did not express Tim-3 or Lag-3 (**Supplementary Figure S7A**) and also did not express PD-1 or CD107a (data not shown) which, together with the elevated IFN-γ production, indicated that the CD8^+^T cells in the ARE^-/-^ mouse ovary and uterus are not exhausted.

**Figure 5:**
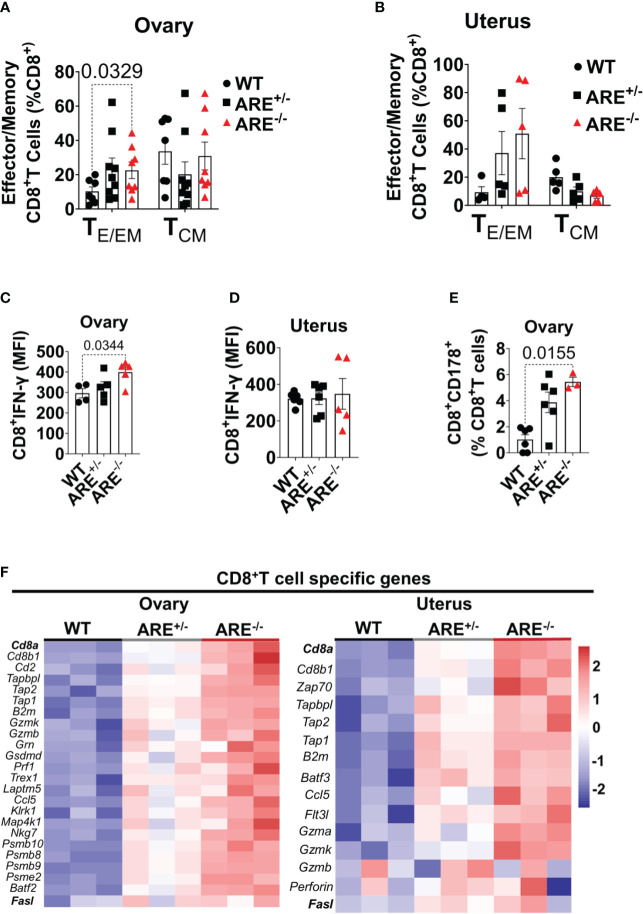
Increased effector CD8^+^ T cells in ARE^-/-^ mouse ovary and uterus. Flow cytometry analysis showing **(A, B)** frequencies of effector/effector memory (T_E/EM_) and central memory (T_CM_) CD8^+^ T cells in the ovary and uterus (*n* = 5–9). **(C, D)** Mean fluorescence intensity of IFN-γ spontaneously released by CD8^+^T cells in the ovary and uterus (*n* = 4–6). **(E)** Frequency of CD8^+^CD178^+^ cells in the ovary (*n* = 3–6). All experiments were performed three independent times. Statistical significance: one- or two-way ANOVA with Kruskal–Wallis or Tukey’s test, respectively. Data represent mean ± SEM. **(F)** Heat maps of the normalized gene expression of CD8^+^T cell-specific genes in the ovary (left) and uterus (right) (*n* = 3). *n* denotes animals per group in all cases.

Next, we asked if ARE^+/-^ and ARE^-/-^ mouse CD8^+^T cells in the ovary and uterus were effector or central memory subsets. Upon activation, CD8^+^ T cells differentiate into effector (or effector memory), (T_E/EM;_ CD44^+^CD62L^-^), and central memory (T_CM;_ CD44^+^CD62L^+^) subsets. The former represents activated cells with rapid effector properties, while the latter represents cells that exert lymph node-homing features with potent proliferative potential (68). CD8^+^T_E/EM_ cells were of higher frequencies in the ARE^-/-^ mouse ovary and uterus compared to the CD8^+^T_CM_ cells (**Figures 5A, B**; **Supplementary Figure S7B**). However, the frequencies of CD8^+^T_CM_ cells were higher in the WT and ARE^-/-^ mouse spleen compared to CD8^+^T_EM_ subsets (**Supplementary Figure S7C**), while both the CD8^+^T_E/EM_ and T_CM_ subsets were lower in ARE^-/-^ mouse lymph nodes (LNs) draining the ovary and uterus compared to the WT (**Supplementary Figure S7C**). These findings indicate increased effector memory cells in the non-lymphoid ARE^-/-^ mouse ovary and uterus. As expected from the ARE^-/-^ mouse phenotype, spontaneous CD8^+^ T cell IFN-γ production increased in the ARE^-/-^ mouse ovary and uterus compared to the ARE^+/-^ and WT mice (**Figures 5C, D** and **Supplementary Figure S7D**), indicative of an effector T cell phenotype (69). In addition, a subset of ARE^-/-^ mouse CD8^+^T cells displayed increased CD178 expression [a protein expressed on activated cytotoxic T lymphocytes (70)] in the ovary (**Figure 5E**), indicative of an activated effector subset. Furthermore, RNAseq confirmed the enrichment of cytotoxic T cell-associated genes in the ARE^-/-^ mouse ovary and uterus, including *Cd8a*, *Cd8b1*, *Gzmk*, *Gzmb*, and *Prf1* or *Perforin* (**Figure 5F** and **Supplementary Tables S3**, **S4**). These data together suggest that effector/effector memory CD8^+^T cells lacking exhaustion markers increased in the ARE^-/-^ mouse ovary and uterus.

### Non-recirculating effector CD62L^-^CD8^+^T cell phenotypes are dominant in the ARE^-/-^ mouse ovary and uterus

**Figure 6:**
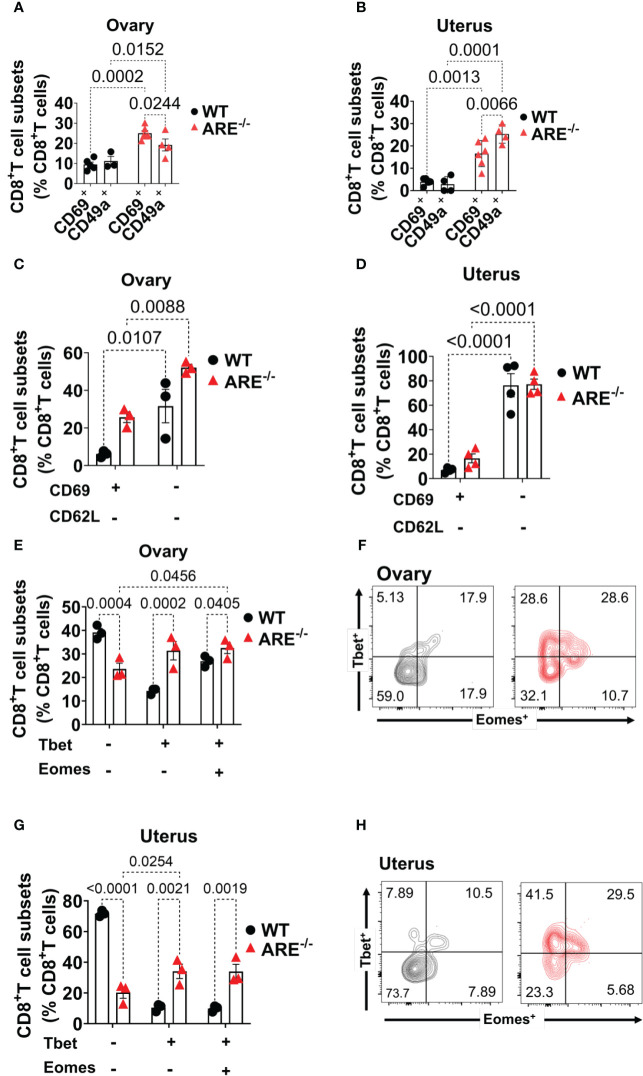
Increased CD8^+^T_EM_ cells in the ARE^-/-^ mouse ovary and uterus. **(A, B)** CD69 and CD49a expression on CD8^+^T cells in the ovary and uterus (*n* = 3–5). **(C, D)** CD8^+^CD62L^+/-^ T cells in the ovary and uterus (*n* = 3–4). **(E–H)** Bar graphs and representative flow cytometry density plots of Tbet and Eomes expression on CD8^+^T cells in the **(E, F)** ovary and **(G, H)** uterus, respectively (*n* = 3). All experiments were performed two independent times. Statistical significance: two-way ANOVA with Tukey’s test. Data represent mean ± SEM, and *n* denotes animals per group.

Next, we asked whether the chronic inflammation in ARE^-/-^ mice favored effector memory CD8^+^T cells (T_EM_) over tissue-resident memory (CD8^+^T cells T_RM_) cells since CD8^+^T cells in the FRT are predominantly tissue-resident (71). CD69 and CD49a are recognized as canonical markers for identifying T_RM_ cells, while the co-expression of CD69 and CD103 is commonly considered a defining feature of conventional T_RM_, specifically in epithelial tissues (72). Therefore, to assess the frequencies of CD8^+^ T_RM_ cells in the ovary and uterus, we investigated the presence and frequencies of CD69^+^CD8^+^T and CD49a^+^CD8^+^T cells. CD69^+^CD8^+^T and CD49a^+^CD8^+^T cell subsets increased in the ARE^-/-^ mouse ovary and uterus compared to those in the WT (**Figures 6A, B**; **Supplementary Figures S8A, B**). In addition, the CD69^+^CD8^+^ T cell subsets increased in the ARE^-/-^ mouse ovary compared to the CD49a^+^ subsets (**Figure 6B**; **Supplementary Figures S8A, B**). Furthermore, there were no significant differences in the frequencies of CD69^+^CD8^+^T cells in ARE^-/-^ mouse spleen and LNs draining the ovary and uterus (**Supplementary Figure S8C**). There were also no differences in CD49a^+^CD8^+^T cells in the WT and ARE^-/-^ mouse spleen and LNs (**Supplementary Figure S7C**). These findings indicate that the local chronic inflammatory environment in the ARE^-/-^ mouse ovary and uterus favored the increase in CD69^+^CD8^+^T cell subsets compared to the WT cohorts. However, though CD69^+^CD8^+^T cells increased in the ARE^-/-^ mouse ovary and uterus, this population represented only ≤30% of CD8^+^T cells (**Figures 6A, B**), with the majority of CD8^+^T cell population lacking CD69 expression.

Next, we asked whether the CD69^+^CD8^+^T cell subsets represented T_RM_ cells, and we investigated this possibility by assessing the co-expression of CD103 and CD69. We found that the WT and ARE^-/-^ mouse ovary had ≤3% of CD69^+^CD103^+^ and ≤10% of CD69^+^CD103^-^ subsets in the WT ovary which decreased in the ARE^-/-^ mouse (**Supplementary Figures S9A, B**). The WT and ARE^-/-^ mouse uterus had ≤2% of CD69^+^CD103^+^ and ≤6% of CD69^+^CD103^-^ subsets in the WT uterus which decreased in the ARE^-/-^ mouse (**Supplementary Figures S9A, C**). In addition, the CD8^+^T cells in the spleen and LNs draining the ovary and uterus did not express CD69 and CD103 (**Supplementary Figure S9D**). Notably, the CD69^+^CD8^+^T subsets in the ARE^-/-^ mouse ovary and uterus comprised approximately ≤30% (**Figures 6A, B** and **Supplementary Figure S9B**), and this suggests that, in this study, the dominant CD8^+^T cell subset in murine ovary and uterus does not express CD69 (CD69^-^CD8^+^T cells).

Further investigation showed that approximately ≥60% of the CD69^-^CD8^+^T cell subset in the ovary and uterus did not express the lymph node (LN) homing receptor L-selectin (CD62L) (73) (**Figures 6C, D**; **Supplementary Figure S10A**). In contrast, a large population (≥60%) of CD8^+^T cells in the spleen and LNs draining the ovary and uterus expressed CD62L (**Supplementary Figure S10B**). Studies indicate that the absence of CD62L and CD69 expression represents non-lymphoid tissue (NLT) recirculating effector memory T cells (T_EM_) (71, 74, 75). Therefore, to determine the phenotype of the dominant CD69^-^CD62L^-^CD8^+^T cell subsets in WT and ARE^-/-^ mouse ovary and uterus, we assessed the expression of Eomes (a T-box transcription factor, TF) and the related homolog T-box TF Tbx21 (Tbet) (76). Eomes and Tbet shape the development of T_RM_ cells such that downregulation of both Tbet and Eomes is required for CD8^+^ T_RM_ cell development in the skin (76, 77). Based on the coordinate expression of Tbet and Eomes, we delineated the CD69^-^CD62L^-^CD8^+^T cells in the ovary and uterus into three different subsets: (i) Tbet^-^Eomes^-^, (ii) Tbet^+^Eomes^-^, and (iii) Tbet^+^Eomes^+^ (**Figures 6E–H**). Approximately 40%–70% of CD69^-^CD62L^-^CD8^+^ T cells were Tbet^-^Eomes^-^ in the WT ovary and uterus, respectively, and represented the most abundant population of CD8^+^T cells (**Figures 6E–H**). However, the Tbet^+^Eomes^-^ and Tbet^+^Eomes^+^ CD8^+^T cell subsets increased in the ARE^-/-^ mouse ovary and uterus and were the prevailing subsets (**Figures 6E–H**), while the Tbet^-^Eomes^-^ subsets were dominant in the spleen and LNs draining the ovary and uterus (**Supplementary Figure S10C**). These findings indicate that the ARE^-/-^ mouse ovary and uterus display a higher proportion of non-recirculating CD8^+^T cells (CD62L^-^ CD69^+^, CD49a^+^) with an effector phenotype (CD62L^-^, T-bet^+^ Eomes^+^) that is further confirmed by increased CD44 and IFN-γ expression.

### CD8^+^T cells in the ARE^-/-^ mouse ovary and uterus do not display conventional resident memory signatures

**Figure 7:**
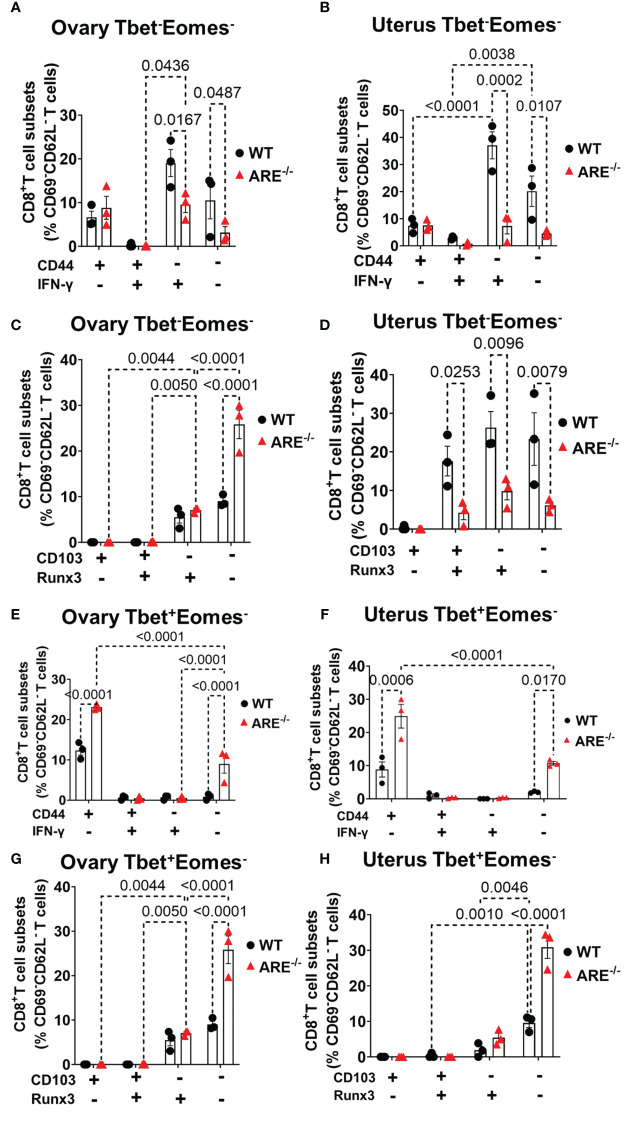
Effector and resident memory status of the dominant CD8^+^CD69^-^CD62L^-^ in the ovary and uterus based on Tbet, Eomes, CD44, IFN-γ, CD103, and Runx3 expression. **(A, B)** Frequencies of CD44 and IFN-γ expression and **(C, D)** CD103 and Runx3 expression on CD8^+^CD69^-^CD62L^-^Tbet^-^Eomes^-^ T cells in the ovary and uterus (*n* = 3). **(E, F)** Frequencies of CD44 and IFN-γ expression and **(G, H)** CD103 and Runx3 expression on CD8^+^CD69^-^CD62L^-^Tbet^+^Eomes^-^ T cells in the ovary and uterus (*n* = 3). All experiments were performed three independent times. Statistical significance: two-way ANOVA with Tukey’s test. Data represent mean ± SEM, and *n* denotes animals per group.

**Figure 8:**
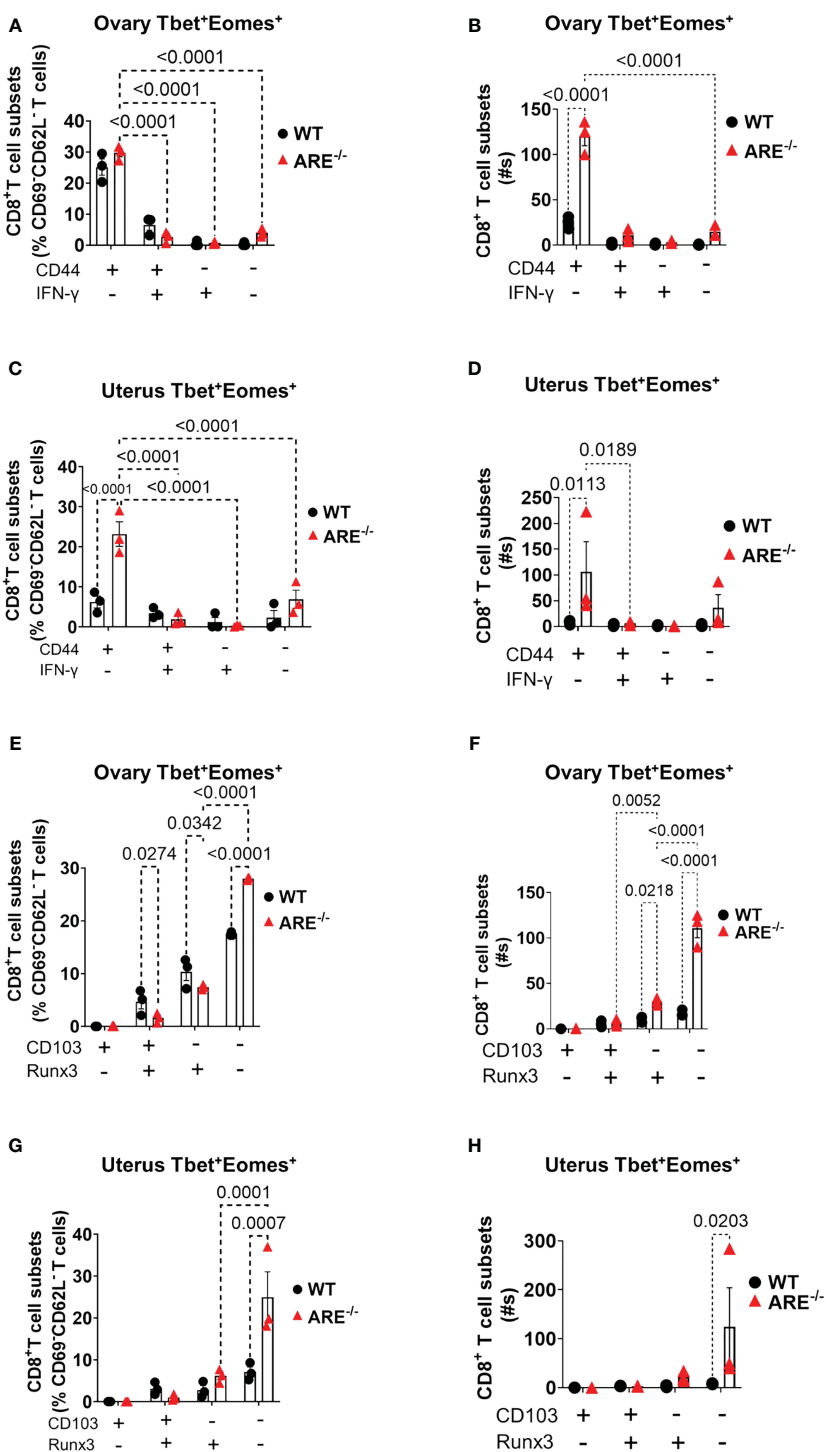
Analysis of T_EM_ and T_RM_ subsets of CD8^+^T cells in the ovary and uterus based on Tbet^+^Eomes^+^ expression. **(A–D)** CD44 expression increased in Tbet^+^Eomes^+^CD8^+^T cells in the ARE^-/-^ mouse ovary and uterus (*n* = 3). **(E–H)** CD103 and Runx3 expression increased in Tbet^+^Eomes^+^CD8^+^T cells in the ARE^-/-^ mouse ovary and uterus (*n* = 3). All experiments were performed two independent times. Statistical significance: two-way ANOVA with Tukey’s test. Data represent mean ± SEM, and *n* denotes animals per group.

Reports indicate that CD103 expression on CD8^+^T cells is regulated by Runx3 (78, 79); therefore, since the dominant CD8^+^T cell subsets in the ovary and uterus in this study did not express CD69, we next investigated the expressions of CD103 and Runx3, including CD44 and IFN-γ, on the CD69^-^CD62L^-^CD8^+^T cells. Phenotypic characterization showed that the CD69^-^CD62L^-^CD8^+^T cells that are Tbet^-^Eomes^-^ primarily expressed CD44^lo^ IFN-γ^+^ and CD103^-^ Runx3^lo^ in both the WT and ARE^-/-^ mice (**Figures 7A–D**; **Supplementary Figures S11–S15**). The expression of Runx3 by WT CD8^+^T cells supports a tissue-resident phenotype (80). However, the Tbet^+^Eomes^-^ CD8^+^T cells were CD44^hi^ IFN-γ^lo^ in the WT and ARE^-/-^ mouse ovary and uterus (**Figures 7E–H**; **Supplementary Figures S11–S15**), suggesting an intermediate T_RM_/T_EM_ or T_RM_/T_CM_ subset (81–84). Conversely, the Tbet^+^Eomes^+^ CD8^+^T cells that are CD69^-^CD62L^-^ subsets were CD44^hi^ and IFN-γ^+^ and did not express Runx3 or CD103 (**Supplementary Figures S11–S15**; **Figure 8**). The data on the phenotypes of Tbet^+/-^/Eomes^+/-^ CD8^+^T cells are summarized in **Tables 1**, **2**.

**Table 1:** Summary of CD69^-^CD62L^-^ CD8^+^T cells in the ovary of WT and ARE^-/-^ mice.

	CD44		IFN-γ		CD103		Runx3	
	WT	ARE^-/-^	WT	ARE^-/-^	WT	ARE^-/-^	WT	ARE^-/-^
**Tbet^-^Eomes^-^**	lo	lo	lo	lo	–	–	lo	lo
**Tbet^+^Eomes^-^**	+	+*	–	–	–	–	lo	lo
**Tbet^+^Eomes^+^**	+	+	+	+	–	–	–	–

a Significantly increased in the ARE^-/-^ mouse (n = 3 mice per group; statistical significance, two-way ANOVA with Tukey’s test; the experiments were performed two independent times).

**Table 2:** Summary of CD69^-^CD62L^-^ CD8^+^T cells in the uterus of wild-type (WT) and ARE^-/-^ mice.

	CD44		IFN-γ		CD103		Runx3	
	WT	ARE^-/-^	WT	ARE^-/-^	WT	ARE^-/-^	WT	ARE^-/-^
**Tbet^-^Eomes^-^**	lo	lo	lo	lo	+	lo	Lo	lo
**Tbet^+^Eomes^-^**	+	+^a^	–	–	+	lo	+	lo
**Tbet^+^Eomes^+^**	+	+	+	+	–	–	–	–

a Significantly increased in the ARE^-/-^ mouse (n = 3 mice per group; statistical significance, two-way ANOVA with Tukey’s test; the experiments were performed two independent times).

A phenotypic investigation on the CD69^+^C62L^-^ CD8^+^T cell subsets indicated that these cells were primarily CD44^+^CD103^+^Runx3^+^Tbet^lo^ Eomes^lo^ IFNγ^+^ (**Supplementary Figures S16–S18**). A further assessment of the CD69^+^C62L^-^ CD8^+^T cells that were positive for Tbet and Eomes (Tbet^+^Eomes^+^) revealed an increase in CD44^+^IFN-γ^-^ and CD44^+^IFN-γ^+^ subsets in the ARE^-/-^ mouse ovary and uterus (**Supplementary Figures S16C, D**). In addition, the expression of CD103 and Runx3 decreased in the ARE^-/-^ mouse ovary and uterus (**Supplementary Figures S16E, F**). These findings indicate that the CD69^+^ CD8^+^T cells produced IFN-γ, though not the dominant subsets, and suggest that these were primarily T_E/EM_ cells. Gating strategies are shown in **Supplementary Figure S19**.

RNAseq revealed the upregulation of the integrin gene, *Itgb7*, in the ARE^-/-^ mouse ovary and uterus (**Table 3**; **Supplementary Tables S5**, **S6**). In addition, *Itga4*, *ItgaD*, *Itgal*, and *Itgb2* were exclusively upregulated in the ovary, while *Itga11* was upregulated solely in the uterus (**Table 3**; **Supplementary Tables S5**, **S6**). We speculate that since a larger population of CD8^+^T cells lacked conventional T_RM_ markers, one or more of the integrins identified by RNAseq in this study may be expressed by the CD69^-^CD49a^-^CD62L^-^ CD8^+^T cells in the ovary and uterus. However, since this was a bulk RNAseq study, further investigations are necessary to confirm if these integrins define CD8^+^T cell phenotypes in the ovary and uterus.

**Table 3:** Significantly upregulated genes encoding integrins identified by RNASeq in ARE^-/-^ mouse ovary and uterus.

Integrin gene	ARE^-/-^ ovary	ARE^-/-^ uterus
***Itgb7* (Ly69)**	+	+
***Itga4* (CD49d)**	+	–
***ItgaD* (CD11d)**	+	–
***Itgal* (CD11a)**	+	–
***Itgb2* (CD18)**	+	–
***Itga11***	–	+

+, present; -, absent (n = 3).

Taken together, these findings suggest that the chronic inflammatory environment in the ARE^-/-^ mouse ovary and uterus reduced the frequency of CD8^+^T_RM_ cells (CD103^+^ and Runx3^+^ cells) and induced an increased infiltration of CD8^+^T_EM_ cells CD44^+^CD62L^-^ cells) expressing T-bet and/or Eomes. A schematic summarizing the altered T_RM_ and T_EM_ CD8^+^T cell subtypes in WT and ARE^-/-^ mouse ovary and uterus is shown in **Supplementary Figure S20**.

### Increased effector memory CD8^+^T cells did not impact oocyte release in ARE^-/-^ mice

To examine whether the infiltration of effector memory CD8^+^T cell within the ovary of mice with elevated IFN-γ impacted oocyte release and egg quality, we subjected WT and ARE (ARE^+/-^ and ARE^-/-^) mice to superovulation [hormone-induced hyperstimulation of the ovaries (85)]. Superovulation allowed the evaluation of ovarian function independent of uterine function (86). ARE^-/-^ mice released eggs after superovulation, which confirmed their ability to ovulate (**Table 4**). Furthermore, we found no difference in fertilization percentage (**Table 4**), indicating that the ARE^-/-^ mouse eggs can be fertilized and undergo the first cleavage division.

**Table 4:** Superovulation, *in vitro* fertilization, and embryo transfer outcome.

Sperm strain	Oocyte strain	No. of female oocyte donors	No. of oocytes harvested	No. of fertilized^a^ embryos	% fertilization	% live birth
**WT**	WT	6	76	45	59.21%	26.67%
**WT**	ARE^+/-^	6	91	51	56.04%	25.49%
**WT**	ARE^-/-^	3	28	17	60.71%	11.80%

a Fertilization was determined based on cleavage to two cells.

To determine the contribution of the uterine environment to the ARE^-/-^ mouse infertility phenotype, fertilized embryos from WT, ARE^+/-^, and ARE^-/-^ female mice were transferred to pseudopregnant B6D2F1 female mice (C57BL/6J × DBA2/J F1), which are known for their improved reproductive performance compared to C57BL/6J female mice (87). The transferred ARE^-/-^ mouse embryos resulted in 11.8% live births, while ARE^+/-^ and WT embryo transfer resulted in 26.67% and 25.49%, respectively (**Table 4**). This finding demonstrated that some ARE^-/-^ embryos can undergo implantation (about half of the occurrence in WT and ARE^+/-^ mice) and be sustained to term under favorable conditions.

Collectively, our data showed that the infiltration of CD8^+^T cells in the ARE^-/-^ mouse ovary and uterus created an unfavorable environment for embryo implantation after oocyte release and fertilization.

### ARE^-/-^ mouse display decreased luteinization and circulating progesterone

**Figure 9:**
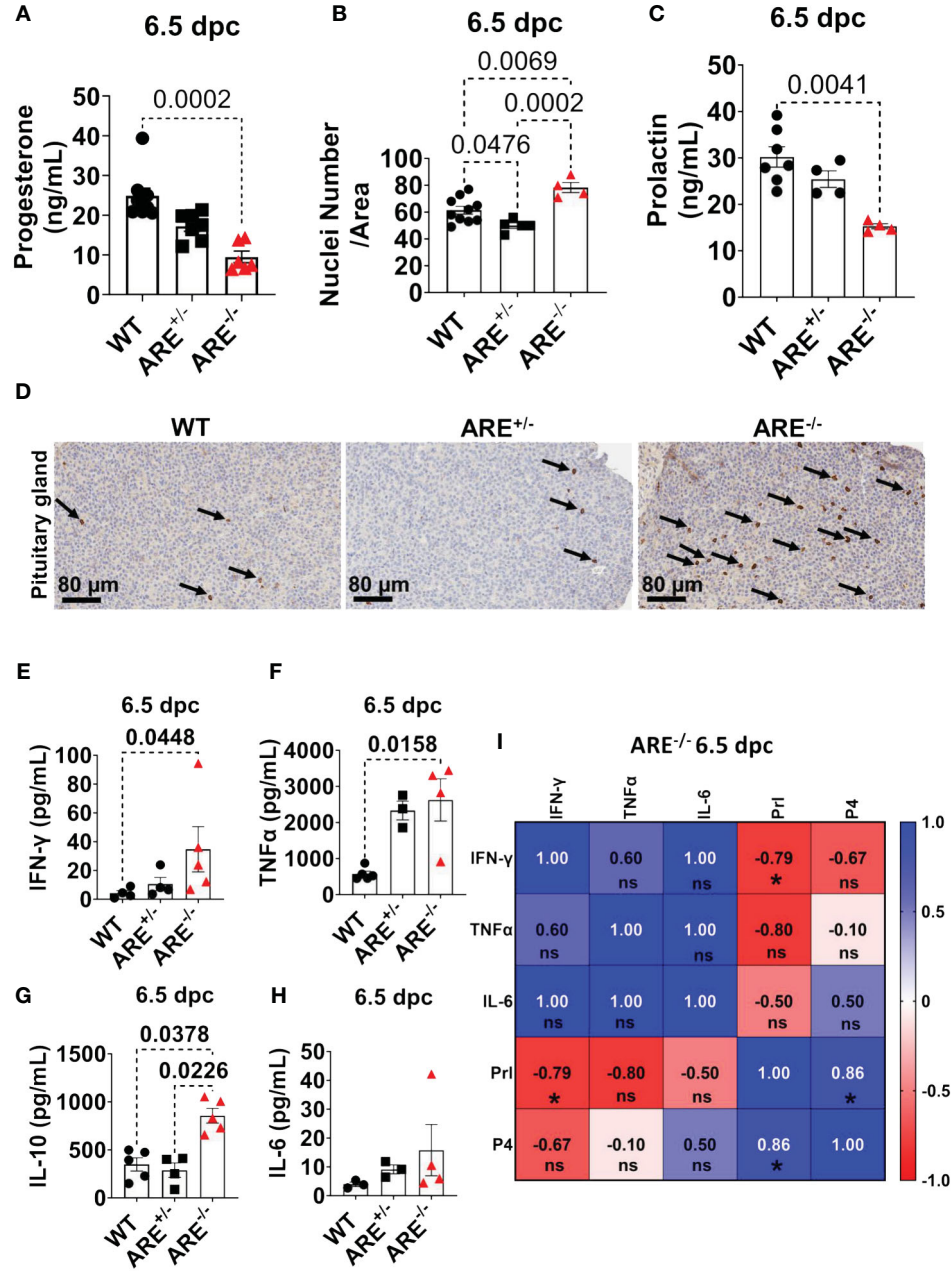
ARE^-/-^ mice exhibited prolactin deficiency in the presence of hypophysitis. **(A)** Circulating progesterone at 6.5 dpc (*n* = 6–8). **(B)** Average nuclei count per defined corpora lutea (CL) area at 6.5 dpc (*n* = 4–5). Each data point corresponds to one CL. **(C)** Circulating prolactin at 6.5 dpc (*n* = 4–7). **(D)** Representative immunohistochemistry of pituitary gland sections showing qualitative CD8^+^T cell infiltration (black arrows) of the anterior lobe (*n* = 4). Plasma levels of **(E)** IFN-γ (*n* = 4–5), **(F)** TNF-α (*n* = 3–5), (**G**), IL-10 (*n* = 4–5), and **(H)** IL-6 (*n* = 3–4). **(I)** Spearman correlation analysis of IFN-γ, TNF-α, IL-6, Prl, and P4 in ARE^-/-^ mice at 6.5 dpc (*n* = 4), * = p < 0.05; ns, not significant. All experiments were performed two independent times. Statistical significance: one-way ANOVA with Kruskal–Wallis test was used except where otherwise indicated. Data represent mean ± SEM, and *n* denotes animals per group.

Since ARE^-/-^ mice ovulate and eggs can be fertilized, we asked whether disruptions in post-ovulation ovarian steroidogenesis impacted ARE^-/-^ mice fertility. To address this question, we measured the circulating progesterone (P4) levels at 6.5 dpc. Following ovulation and in response to gonadotropins, granulosa and theca cells differentiate from estrogen-producing cells to cells that primarily synthesize P4, a process known as luteinization. Steroidogenic CL cells synthesize P4 to initiate uterine quiescence and glandularization in preparation for pregnancy (88, 89). We found decreased circulating P4 concentrations in the ARE^-/-^ mice compared to WT cohorts (**Figure 9A**). Next, we examined cytoplasmic hypertrophy, a hallmark of luteinization where fewer nuclei per area specify a greater cytoplasmic volume (90). To determine whether the degree of hypertrophy differed between ARE and WT mice, we counted the number of nuclei per defined CL area at 6.5 dpc. The CL from ARE^-/-^ mice had more nuclei per area than the WT and ARE^+/-^ cohorts (**Figure 9B**; **Supplementary Figure S21A**). The decreased CL hypertrophy in the ARE^-/-^ mouse ovaries represented reduced CL luteinization (90), which supported the decreased P4 in ARE^-/-^ mice (**Figure 9A**) and corresponded to the CD8^+^T cell infiltration of the ARE^-/-^ mouse ovary and CL (**Figures 3E–H**).

These findings together indicate that the ARE^-/-^ mice have reduced CL function in the presence of increased CD8^+^T cell infiltration.

### Prolactin deficiency is associated with reproductive dysfunction in ARE^-/-^ mice

In murine models, prolactin (Prl) is a critical hormone for CL function and maintenance, including post-ovulation requirements for a successful pregnancy (34, 35, 91–94). The survival of murine CL during pregnancy (post-mating) depends on a critical Prl threshold initiated by the mating stimulus. A deficient Prl post-mating impedes CL of the cycle conversion to CL of pregnancy and, thus, infertility (35, 92). Therefore, since CD8^+^T cells infiltrate the dysfunctional ARE^-/-^ mouse CL, we asked whether Prl production was altered in the ARE^-/-^ mice. To investigate this line of inquiry, we examined circulating Prl concentrations. We observed decreased circulating Prl in the ARE^-/-^ mice at 6.5 dpc compared to the WT cohorts (**Figure 9C**), indicating Prl deficiency (95). We found no statistically significant difference between the Prl levels in WT and ARE^+/-^ mice (**Figure 9C**). Furthermore, we observed CD8^+^T cell infiltration in the anterior lobe of the ARE^-/-^ mouse pituitary gland (**Figure 9D**; black arrows), which is indicative of lymphocytic hypophysitis (lymphocytic infiltration of the pituitary gland) (40, 96).

At 6.5 dpc, pro-inflammatory cytokines and IL-10 in the ARE^-/-^ mice remained persistently elevated (**Figures 9E–H**) and significantly correlated with the decreased Prl and P4 (**Figure 9I**).

Collectively, our findings reveal that the ARE^-/-^ mice experience pituitary inflammation and Prl deficiency, in addition to ovarian inflammation, decreased luteinization, and P4 deficiency, which suggest that these events contributed to the ARE^-/-^ mouse infertility.

### ARE^-/-^ mouse CD8^+^T cells target the ovaries and the anterior pituitary

To establish the direct contribution of elevated IFN-γ on CD8^+^T cell infiltration into the ovaries and pituitary glands, we administered murine recombinant IFN-γ (rmIFN-γ) (240 pg/mL i.p.) bi-daily to non-pregnant, cycle-synchronized WT female mice (4–6 months old) for 3 weeks (**Supplementary Figure S21B**). The mice were euthanized at estrus. Notably, we observed a significant infiltration of CD8^+^T cells in the ovaries of WT mice treated with rmIFN-γ compared to the PBS control group (**Supplementary Figures S21C, D**). This finding demonstrates the potential of elevated systemic IFN-γ to disrupt immune tolerance in the ovaries.

**Figure 10:**
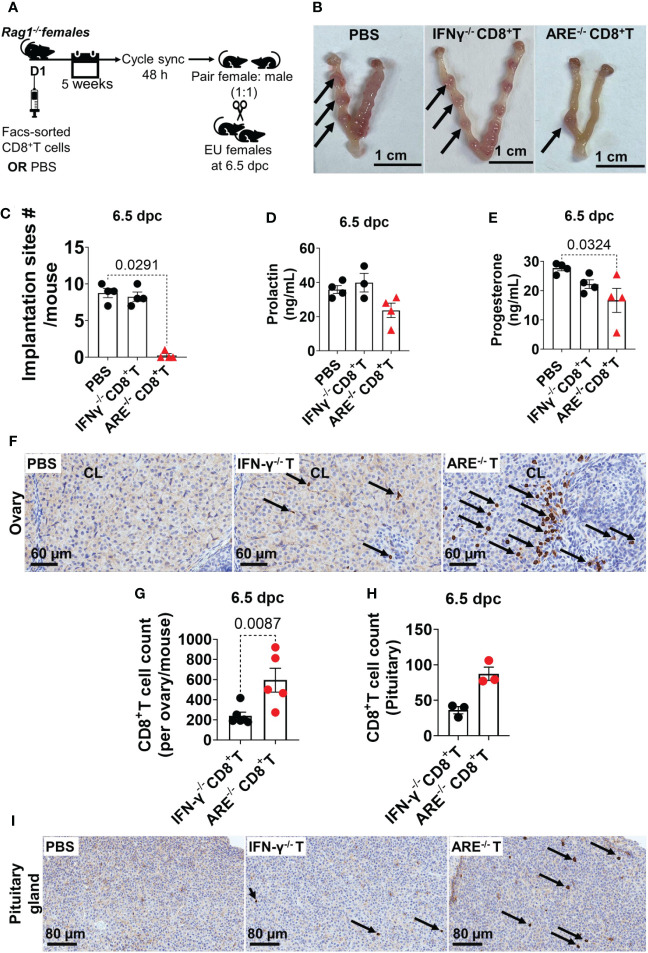
ARE^-/-^ mouse CD8^+^ T cells targeted the ovary and pituitary. **(A)** Protocol illustration for the adoptive transfer and mating of CD8^+^T cell recipient and PBS-control *Rag1* ^-/-^ female mice. sync, synchronize; EU, euthanize. **(B)** Representative whole-mount 6.5-dpc uteri showed several implantation sites (black arrows) in pregnant PBS-control and IFN-γ^-/-^ CD8^+^T cell recipient *Rag1* ^-/-^ mice, while the ARE^-/-^ CD8^+^T cell recipient group showed decreased implantation sites (*n* = 4). **(C)** ARE^-/-^ CD8^+^T cell recipient *Rag1^-/-^* female mice had decreased implantation sites at 6.5 dpc compared to IFN-γ^-/-^ CD8^+^T cell recipients and PBS-controls (*n* = 4). **(D)** Circulating prolactin (*n* = 4) and **(E)** progesterone (*n* = 4) in CD8^+^T cell recipient and PBS-control *Rag1* ^-/-^ female mice. **(F)** Representative immunohistochemistry (IHC) images showing the distribution of CD8a-positive cells (black arrows) in the ovary of *Rag1* ^-/-^ female mice (*n* = 4). **(G)** IHC CD8a^+^T cell counts in the ovary and **(H)** pituitary glands (*n* = 3). **(I)** Representative IHC images showing the distribution of CD8a-positive cells (black arrows) in the pituitary of *Rag1* ^-/-^ female mice (*n* = 4). IFN-γ^-/-^ CD8^+^T or IFN-γ^-/-^ T, recipient of IFN-γ^-/-^ CD8^+^T cells; ARE^-/-^ CD8^+^T or ARE^-/-^ T, recipient of ARE^-/-^ CD8^+^T cells. All experiments were performed two independent times. Statistical significance: one-way ANOVA with Kruskal–Wallis test or two-tailed Student’s *t*-test with Mann–Whitney analysis was used. Data represent mean ± SEM, and *n* denotes animals per group.

Next, we investigated whether ARE^-/-^ mouse CD8^+^T cells were the primary immune cells driving ARE^-/-^ mouse infertility. Lymphoid CD8^+^T cells were FACS-sorted from ARE^-/-^or IFN-γ^-/-^ mice and adoptively transferred to cycle-synchronized lymphopenic 4-month-old *Rag1^-/-^* female mice. A control group received PBS (**Figure 10A**). After 5 weeks, the recipient mice were mated with WT male mice (1:1), monitored for copulatory plugs, and euthanized at 6.5 dpc (**Figure 10A**). The mice that received ARE^-/-^ mouse CD8^+^T cells showed significantly decreased implantation sites (**Figures 10B, C**), including decreased P4 and Prl levels (**Figures 10D, E**), compared to the PBS and IFN-γ^-/-^ CD8^+^T cell recipient groups. IHC revealed the infiltration of ARE^-/-^ mouse CD8^+^T cells in the ovaries (including CL localization) and pituitaries of *Rag1* ^-/-^ hosts (**Figures 10F–I**), though not significantly to the uterus (**Supplementary Figures S21E, F**). These findings indicate that the aberrant ARE^-/-^ mouse CD8^+^T cells targeted tissues modulated by Prl (anterior pituitary and CL (97)) and caused infertility in the ARE ^-/-^ mice.

Collectively, our findings effectively replicate the ARE^-/-^ mouse infertility phenotype and indicate that the ARE^-/-^ mouse reproductive dysfunction is driven by CD8^+^T cells.

A comparison of the reproductive phenotypes in SLE patients, other lupus mouse models, and ARE^+/-^/ARE^-/-^ mice is listed in **Table 5**.

**Table 5:** Comparison of reproductive phenotypes in systemic lupus erythematosus patients, ARE mice, and other lupus mouse models.

	Patients	ARE^-/-^ mice	ARE^+/-^ mice	Other^a^ lupus mouse models
**Irregular cycles**	+	+	+	+ (52)
**Fertilizable eggs**	+ (59)	+	+	+ (52, 53)
**Decreased implantation**	+ (59)	–	+	(54)
**Implantation failure**	+ (59)	+	–	- (54)
**Decreased offspring** number	+ (58)	–	+	(54)
**Infertility**	+ (58, 59)	+	–	- (54)
**Elevated IFN-γ**	+ (98, 99)	+	+	(51)

+, present; -, absent.

a Lupus mouse models indicated in this article.

## Discussion

Patients with SLE (and similar autoimmune diseases) experience elevated IFN-γ levels and altered circulating Prl associated with disrupted pregnancy and fertility outcomes (55, 56)) as seen in this study. Here we report for the first time that chronic elevated IFN-γ levels in lupus-prone ARE^-/-^ mice disrupt fertility marked by lymphocytic hypophysitis, prolactin deficiency, and infiltration of CD8^+^T_EM_ cells in the ovary and uterus. Importantly, we highlight a CD8^+^T cell-mediated mechanism of reproductive dysfunction due to elevated systemic and local IFN-γ in ARE^-/-^ mice. Specifically, the increased T_EM_ CD8^+^T cells in the ARE^-/-^ mouse ovary and uterus support an activated effector phenotype and provide a cellular mechanism for reproductive dysfunction in SLE associated with Prl dysregulation. Our findings suggest that increased CD8^+^T_EM_ subsets in the ARE^-/-^ mouse ovary and uterus contributed to reproductive dysfunction in an environment of systemic autoimmunity and chronic inflammation and are consistent with reports of increased CD8^+^T cell subsets in women with recurring miscarriage, including the presence of CD8^+^CD69^-^CD103^-^ subsets that retain the capacity to produce IFN-γ (100). In this study, the increased infiltration of CD8^+^T cells in the ARE^-/-^ mouse ovary and uterus post-mating suggests a loss of tolerance to the CL of pregnancy or the fetus. In addition, a possible explanation for the CD8^+^T cells targeting the reproductive tract and pituitary in this study is molecular or antigenic mimicry, which is the similarity of different antigens or the existence of the same antigen in different tissues (101, 102).

Studies indicate that the systemic administration of exogenous IFN-γ in the peri-implantation period prevents implantation and inhibits pregnancy maintenance (4, 103). In addition, women with elevated IFN-γ, while undergoing a single day-5 blastocyst transfer, failed to conceive (as modeled in the ARE^-/-^ mice). However, women with a moderate but transient increase in IFN-γ successfully conceived (104), supporting the relevance of IFN-γ threshold levels during the peri-implantation period. Furthermore, patients with recurrent spontaneous abortion and repeated implantation failure had increased IFN-γ and T cells (105). Thus, women with autoimmune or chronic inflammatory diseases where IFN-γ is elevated, as seen in our ARE^-/-^ mice, have difficulty conceiving or maintaining pregnancies (106, 107). Our study showed that fertility disruption and outcome are exacerbated by increasing IFN-γ levels and driven by CD8^+^T cells as observed by different reproductive phenotypes in the IFN-γ^-/-^, ARE^+/-^, and ARE^-/-^ mouse models.

From an endocrine standpoint, Prl deficiency was another critical finding in this study, and Prl is necessary for a successful murine pregnancy (34, 91). In mice, CL is formed following ovulation; however, a mating stimulus and consequent diurnal Prl secretion are required to differentiate the CL of the cycle into the CL of pregnancy (108, 109). Therefore, Prl in murine reproduction is critical during the peri-implantation period to promote and maintain CL function (108). However, IFN-γ inhibits anterior pituitary Prl secretion (110), and our findings showed that a primary disruptive event caused by elevated IFN-γ in the pituitary–ovarian axis is Prl deficiency. Notably, the ARE^-/-^ mouse reproductive phenotype mimics clinical reports where Prl is deficient or altered in women due to chronic inflammatory diseases (111) [attributable to autoimmunity, infections, or use of therapeutic checkpoint inhibitors (112)]. Our findings highlight that IFN-γ and Prl levels are significantly correlated. Furthermore, women with altered Prl levels, such as hypoprolactinemia, have reduced fertilization and cleavage rates (113). These findings together support a correlation between IFN-γ and altered Prl. Though the specific cell type targeted by ARE^-/-^ mouse CD8^+^T cells in the pituitary, ovary, and uterus could not be extensively examined in this study due to the severe ARE^-/-^ mouse infertility, the differences in fertility outcome between the ARE^+/-^ and ARE^-/-^ mice include the observation that ARE^+/-^ mice had higher Prl and lower IFN-γ levels compared to the ARE^-/-^ mice and that acute elevation of IFN-γ is sufficient to break tolerance mechanisms in the ovaries of healthy mice. Further support for our findings with Prl comes from studies where mice deficient in Prl or Prl receptor genes are infertile specifically due to luteal defect and implantation failure (35, 92, 93, 114), as what occurred in the ARE^-/-^ mice. Specifically, Prl^-/-^ mice have irregular reproductive cycles but show all cycle phases, ovulate normally, and mate successfully with males, but they do not get pregnant due to implantation failure secondary to CL defect (92), a phenotype observed in the ARE^-/-^ mice. In addition, ARE^-/-^ mice share similar reproductive and hormonal phenotypes with female Prlr^-/-^ and STAT5^-/-^ mice that exhibit prolactin deficits (115). Though Prl is not essential for implantation and pregnancy success in humans as in murine models (116), it is important to note that Prl dysregulation consistently occurs in women with autoimmunity or chronic inflammatory disorders where IFN-γ is elevated (117–119), which supports a connection between IFN-γ, Prl signaling, and fertility. In particular, our findings highlight that IFN-γ levels significantly correlated with Prl and suggest a correlation between threshold IFN-γ levels and fertility outcome.

Under normal conditions, transient *Ifng* transcription occurs post-fertilization in mouse and human embryos at the two-cell stage (120, 121), which must be downregulated or decreased sufficiently at the blastocyst stage (7, 120). Therefore, while IFN-γ promotes fertilization and preimplantation embryonic development (7), its reduction in the peri-implantation stage is critical for successful pregnancy. The contribution of embryos from homozygous male and female pairing to infertility in this study is unclear, and the elevated IFN-γ level led to breeding difficulties that severely limited the number of animals available for further study. Nonetheless, we propose that the resulting embryos from WT male mice vs. ARE^-/-^ female mice in this study produced less IFN-γ than would occur in homozygous pairing and that the IFN-γ produced by the embryos in this study would be comparable to the levels in embryos from heterozygous pairing (ARE^+/-^ vs. ARE^+/-^) which have successful pregnancies [we repopulate ARE^+/-^ and ARE^-/-^ colonies from heterozygous pairings (57)]. Therefore, since the gestational anomalies of ARE^-/-^ female mice were not reversed with WT vs. ARE^-/-^ embryos, our findings suggest that the maternal FRT environment largely contributed to the ARE^-/-^ female infertility. Moreover, mildly elevated IFN-γ in this study (represented by the ARE^+/-^ mice) was not critical to the progression of implantation. However, studies on blastocysts from the WT vs. ARE^-/-^ pairing would be needed to confirm whether embryo competency contributed to infertility in an elevated IFN-γ environment.

To summarize, findings from the current study provide additional evidence to current knowledge that systemic elevation or pathological concentrations of IFN-γ in autoimmunity or other chronic inflammatory diseases are detrimental to successful pregnancy and that infertility can occur as a secondary response to systemically elevated IFN-γ. Ashkar and Croy propose that IFN-γ at <10 U/mouse is essential for the normalcy of implantation sites and maintenance of decidual cell viability (8). However, Raghupathy suggested that Th1 cytokines, particularly IFN-γ, are incompatible with successful pregnancy (122), leading to the conundrum of whether the fertility problems are generated by IFN-γ or secondary to failure of other IFN-γ-mediated mechanisms (8). Whether an optimal level of IFN-γ must be maintained by the preimplantation human embryo to increase pregnancy success rates, particularly in women with SLE or chronic inflammatory diseases, merits further investigation. Nonetheless, the current study contributes to the growing awareness of how we can use our knowledge of adverse events in the FRT associated with inflammatory cytokines and IFNs to develop therapies that prevent or reduce pregnancy complications. Importantly, this study demonstrated a clear role for elevated IFN-γ-mediated CD8^+^T cell-driven infertility due to CL dysfunction. If we can understand the subsets of CD8^+^ T cells or interferon-stimulated genes (ISGs) specific to CD8^+^ T cells in the reproductive tract that contribute to reproductive pathology differently from those that control pathogens, we may be able to develop immunotherapies that target the reproductive-pathology-specific ISGs or CD8^+^T cell subsets while sparing those that are necessary to control pathogens.

In conclusion, CD8^+^T cells promote pituitary and CL dysfunction in ARE^-/-^ mice with SLE and elevated systemic IFN-γ, resulting in implantation defects and infertility. Although multiple factors potentially determine the type of fertility defect in chronic inflammation, this study provides new insights into critical upstream events and cellular immunological mechanisms in the context of elevated systemic IFN-γ and fertility disruption in SLE.

## Materials and methods

### Mice

C57BL/6J mice were utilized in the current study and included wild type (WT), *Rag1* ^-/-^ [JAX stock #002096 (123)], IFN-γ^-/-^ [JAX stock #002287 (124)], and mice heterozygous and homozygous for the ARE replacement (ARE^+/-^ and ARE^-/-^, respectively) aged 4–6 months. The generation of ARE mice has been described (57). Briefly, a 162-nt AU-rich element (ARE) sequence in the 3′ untranslated region (3′UTR) of the *Ifng* gene was deleted *via* the Cre/*lox* system and replaced by electroporation with a random sequence. The recombined locus was retrieved, cloned, and introduced into a C57BL/6-129 hybrid embryonic stem cell line to generate chimeric mice. Subsequently, ARE lines, derived from individual embryonic stem cell clones, were selected and backcrossed over 100 generations into C57BL/6 mice. The details of the genotyping protocol are provided in the **Supplementary File**. The mice were housed and bred at the National Cancer Institute—Frederick animal facility. On the day of the experiments, we measured the body weights and performed vaginal lavage (as described in the estrous cycle staging method section). The mice were euthanized under isoflurane anesthesia, and the ovaries, uteri, secondary lymphoid tissues (spleen and lymph nodes) including lymph nodes draining the ovary and uterus (medial and iliac lymph nodes), and pituitary were harvested aseptically, weighed, and placed in Roswell Park Memorial Institute (RPMI) 1640 medium (Corning) supplemented with 10%–20% fetal bovine serum (FBS), 100 units/mL penicillin, 10 µg/mL streptomycin, and 2 mM L-glutamine or in fixative [4% paraformaldehyde (PFA) or 10% neutral buffered formalin (NBF)]. Whole blood was collected and placed in plasma separator tubes. Care and experimentation of animals were performed according to the protocols approved by the National Cancer Institute (NCI) Animal Care and Use Committee (Frederick, MD, USA).

### Estrous cycle staging

Vaginal lavage (VL) was collected to monitor estrous stages (125, 126). A description of the estrous cycle phases is provided in the **Supplementary File**. VL (50–100 µL) was placed on clean microscope slides (Corning, USA) and allowed to air-dry at room temperature. The dried slides were fixed with cold methanol and stained with 0.01% methylene blue (Sigma Aldrich, St. Louis, MO, USA) (125, 126) before viewing under a microscope (Nikon Eclipse E600, USA) to determine the cycle stages. The female mice were placed in urine-infused spent male beddings 48 h before the experiments to harmonize the estrus cycle stages (127), and VL was collected at the end of the experiments to determine the cycle stage.

### Fertility and pregnancy investigation

Female mice were mated with fertile male mice of the same C57BL/6J background at a ratio of 1:1 (female/male) (**Figure 1A**). We examined the female mice for copulatory plugs the day after pairing and every morning after that. Copulatory plugs are produced from coagulatory proteins in male semen, which harden and adhere tightly to the female vaginal epithelium after mating (128). Therefore, copulatory plugs confirm that mating occurred. The day copulatory plugs were seen was noted as 0.5 days post-coitum (dpc). Mice with copulatory plugs were allowed to litter or euthanized at 6.5 dpc (**Figure 1A**).

Ovaries, uteri, pituitary glands, spleen, and lymph nodes were harvested from euthanized mice and utilized for either flow cytometry, histological examination, or fluorescence-activated cell sorting (FACS). Blood was additionally collected and placed in plasma separator tubes for hormone or cytokine analysis.

### Single-cell dissociation and isolation

The ovaries, uteri (reproductive tissues), lymph nodes draining the ovary and uterus, and spleen (lymphoid tissues) were dissociated for single-cell preparations. Briefly, the ovary and uterus were dissected and placed in cold RPMI. Fat and connective tissues were removed from the ovaries and uteri before transfer into a digestion mix (0.01 mg/mL collagenase type IV; Sigma Aldrich, USA), and in some cases, four units of DNase I (Sigma Aldrich, USA) were added and made up to 10 mL with supplemented RPMI and equilibrated to room temperature. Next, the ovary and uterus were cut into small pieces using McPherson–Vannas scissors before digestion (custom program 37°C_ 20 min_rpr 400) on the gentleMACS Octo Dissociator with heaters (Miltenyi Biotec, USA). The cell suspension was placed immediately on ice at the end of the program, and 10 mL RPMI was added to quench the enzyme reaction. Next, the suspension was filtered through a 40-µM cell strainer followed by 2 × 2.5 mL washes with RPMI. Finally, we centrifuged the resultant cell suspension at 1,200 rpm for 5 mins in a centrifuge at 4°C. The resulting pellet was resuspended in cold phosphate-buffered saline (PBS; Quality Biological, Gaithersburg, MD, USA) and counted for subsequent staining with selected monoclonal antibodies (**Supplementary Table S1**) as required (125).

We obtained leukocyte cell counts using a Sysmex KX-21N Hematology Analyzer cell counter (Roche) and total cell counts using a Cellometer Auto T4 (Nexcelom Bioscience, USA).

### Flow cytometry

The ovary, uterus, and spleen tissue cell pellets (2–5 × 10^6^ cells, respectively) were placed in tubes for flow cytometry processing. Next, dead cells were stained for exclusion using Zombie Aqua viability stain (Biolegend, San Diego, CA, USA) (1:50) by incubating the cells for 30 min at room temperature in the dark and washing once with FACS buffer. The resulting cell pellets were resuspended in 20 µL of 1:10 dilution of Fc Block CD16/CD32 (clone 2.4G2, BD Biosciences, San Jose, CA, USA) and incubated for 10 min at room temperature (RT). Next, cells were washed with FACS buffer (1 mL) and resuspended in endotoxin-free PBS for flow cytometry assessment. The conjugated antibodies used are indicated in **Supplementary Table S7**. For surface stains, fluorophore-conjugated antibodies were added to the tubes and incubated for 30 min in the dark at 4°C in the presence of Fc blocking antibody. The cells were subsequently washed with FACS buffer, centrifuged at 1,500 rpm for 5 min at 4°C, and resuspended in FACS buffer for flow cytometry acquisition. For the detection of IFN-γ without stimulation and other intracellular markers, the cells were fixed and permeabilized in a fixation/permeabilization buffer solution with monensin (BD Biosciences, USA) according to the manufacturer’s instructions. Unstained samples, stained compensation beads, and single-stained samples were used to set appropriate the PMT voltages and calculate the compensation. Florescence-minus-one-stained cells (FMOs) were also included as additional controls during gating to ensure the accurate identification of cell types. All flow cytometry experiments were performed using a BD Fortessa or BD FACS Symphony A5 and BD FACSymphony S6 Cell sorter (BD Biosciences) for sorted cells with FACS Diva software (BD Biosciences). The results were analyzed with FlowJo software (v. 10.9 BD Biosciences, USA). Debris and unlysed erythrocytes were excluded from all populations with the FSC-A by the SSC-A scatter gate, while the target cells of interest were gated on viable cells.

### Histopathology and immunohistochemistry

The ovary and uterus were fixed in 4% PFA and the pituitary tissues in NBF and paraffin-embedded. We generated slides from paraffin blocks and sectioned the ovary, uterus, and pituitary at 5 μm and stained the slides using the Sakura® Tissue-Tek® Prisma™ automated stainer. The slides were dewaxed using xylene and hydrated using a series of graded ethyl alcohols.

Commercial hematoxylin, clarifier, bluing reagent, and eosin-Y were used to stain for hematoxylin and eosin evaluation (H&E). In addition, a regressive staining method was used. This method intentionally overstains tissues and uses a differentiation step (Clarifier/Bluing reagents) to remove excess stains. Upon completion of staining, the slides were coverslipped using the Sakura® Tissue-Tek™Glass® automatic coverslips, dried, and viewed under a microscope.

Antibodies specific for CD8a were processed on the BondRX autostainer (LeicaBiosystems). Following antigen retrieval with citrate buffer (Bond Epitope Retrieval 1), sections were incubated for 30 min with CD8a (1:50, eBioscience, USA). Staining was completed with rabbit anti-rat IgG secondary antibody and Bond Polymer Refine Detection Kit (Leica Biosystems), with the omission of the post-primary reagent. An isotype control reagent was used in place of the primary antibody for the negative control.

We captured the images using the Aperio Scanscope AT2 whole-slide scanner and digitized slides containing the FFPE tissue sections. Image staining intensity and quantification of IHC-positive cells were evaluated using Image J (NIH) automated algorithms to assess the total number of positive cells.

CL and antral follicles were identified and counted in the H&E-stained ovarian sections. The CL or antral follicle was marked and tracked in each section throughout which it appeared in the ovary, and the total number of sections in which the CL or antral follicle was present was recorded. This procedure was repeated for every CL and antral follicle. CL’s nuclear/cytoplasmic ratio was measured by taking individual images of the total CL per ovary at a scale of 30-µm magnification. The Aperio Scanscope AT2 whole-slide scanner was then used to mark, track, and count the number of nuclei present within approximately 61 × 61-µm sections in the imaged areas.

### Hormone and cytokine measurements

Blood was collected in plasma separator tubes, and plasma was separated by centrifuging at 10 g for 15 min at 4°C. The processed plasma samples were stored at -80°C until use.

For hormone assays, validated mouse ELISA kits for prolactin and progesterone (MyBioSource: catalog # MBS722668 and MBS3806328, respectively) were used according to the manufacturer’s instructions. Mouse prolactin recombinant protein (PeproTech, catalog # 315-16-50UG) and progesterone (Sigma-Aldrich, catalog # P7556-100MG) were used for validation. The samples were tested in duplicates, and the reaction was read at 450 nm as the primary wavelength.

For cytokine measurements, plasma samples were analyzed by combining electrochemiluminescence and arrays in multi-spot plates using the Meso-Scale Discovery custom mouse Multiplex kit for 10 factors (Mesoscale Diagnostic, MSD). All samples were tested in duplicate and read on MSD QuickPlex SQ 120 imager/reader. All cytokine values were reported in units of pg/mL [see **Supplementary Methods** (under cytokine measurements) for further details].

### Adoptive CD8^+^T cell transfer

*Rag1* ^–/–^ female mice (4 months old) were administered with intraperitoneal (i.p.) injections of FACS-sorted CD8^+^T cells (3 × 10^5^ cells/mouse) from lymphoid tissues of age-matched ARE^-/-^ or IFN-γ^-/-^ female mice. *Rag1* ^–/–^ mice injected with PBS i.p. were included as controls. After 5 weeks, blood was collected, and plasma was isolated for hormone measurements as described in the hormone assay section. Ovarian, uterine, and pituitary tissues were harvested for histopathological evaluation. In some cases, recipient *Rag1^-/-^* mice were mated with fertile males and euthanized at 6.5 dpc. In all cases, the estrous cycles were synchronized by placing in urine-infused spent male beddings 48 h before experiments, and estrous cycle stages were assessed at the end of experiments.

### RNA sequencing and analysis

Ovarian and uterine tissues from non-pregnant WT, ARE^+/-^, and ARE^-/-^ mice were placed in RNAlater (Invitrogen) and stored at -80°C until needed. RNA was extracted from the ovary and uterus using the Direct-zol RNA extraction kit (Zymo) according to the manufacturer’s instructions. Quality control of the samples was determined by assessing the RNA integrity number and then used for library preparation and sequencing. Sequencing was performed by Novogene (Sacramento, CA) on the NovaSeq 6000 platform (PE150). RNA-sequencing data are available in the GEO database under accession number GSE237306.

FASTQ reads from bulk mRNA sequencing libraries were trimmed and paired using Trimmomatic (v0.39). STAR (v2.7.10b) was used to index the soft-masked genome. The files used for the soft-genome index (“GRCm39.primary_assembly.genome.fa” and “gencode.vM32.primary_assembly.basic.annotation.gtf”) were downloaded from “gencodegenes.org/mouse/”. Next, the trimmed and paired mRNA FASTQ files were mapped to the soft-masked genome using STAR (v2.7.10b). Once the mRNAs were mapped to the genome, featurecounts in the Subread package (v2.0.4) were utilized to count the mRNAs that mapped to annotated genes. The count matrix files were then subjected to differential gene expression analysis in R (v4.2.2) using DEseq2 (v1.38.3). For pairwise comparisons, significant genes were defined as those with a false discovery rate <0.05. A more stringent cutoff for genes defined as significant was used for LRT analysis (false discovery rate < 1 × 10^-4^) due to known leniencies of this analysis. Once significant genes were identified using LRT, differentially expressed clusters were unbiasedly identified using “degPatterns” from “DEGreport” (v1.34.) The results of differential gene expression analysis were used as input for ClusterProfiler (v4.6.2) to run Gene Ontology (GO) enrichment using gene set enrichment analysis (GSEA). The mouse GO terms were obtained from the annotation package org.Mm.eg.db (v3.16.0). The DESeq2-derived t-statistic was used to rank genes. Only “biological process” (BP) terms were considered for this analysis. We used 10,000 permutations, a minimum gene set size of 3, and a maximum gene set size of 800 to compute enrichment. Terms with a false discovery rate <0.05 were considered significantly enriched in the gene list. ClusterProfiler (v4.6.2) was also used for the over-representation analysis of LRT-derived gene lists. Only “biological process” (BP) terms were considered for this analysis. We utilized a minimum gene set size of 15 and a maximum gene set size of 500 to compute the enrichment. Terms with a false discovery rate <0.05 were considered significantly over-enriched in the gene list compared to the “universe” (all genes with expression detected in the dataset). Venn diagrams were created using the Eulerr package (v7.0.0). All heat maps were obtained using the heatmap package (v1.0.12).

### Quantitative RT-PCR

RNA was isolated from the ovaries and uteri using a Directzol RNA MiniPrep kit (Zymo Research, USA) and reversed-transcribed to synthesize cDNA with a Superscript III cDNA kit (Invitrogen) according to the manufacturer’s instructions. RNA quality and concentration were determined using Nanodrop 2000c, and 1 µg RNA was used for cDNA synthesis with the Superscript II cDNA synthesis system (Invitrogen Corp., Carlsbad, CA, USA). Quantitative real-time PCR (qRT-PCR) was performed on a Roche LightCycler 480 PCR machine with gene-specific Taqman probes (Thermo Fisher) for murine *Ifng*. The target gene expression level was quantified after normalization to *Hprt* expression. The primers and IDs are listed in **Supplementary Table S8**.

### Exogenous acute rmIFN-γ administration to WT female mice

In some experiments, we administered murine recombinant IFN-γ (rmIFN-γ; Shenandoah Biotechnology Inc.) (240 pg/mL i.p.) every other day to WT female mice at 4 to 5 months of age for 3 weeks (the cycles were synchronized by placing in urine-infused spent male beddings 48 h before the experiments). After 3 weeks, the mice were euthanized, and the ovaries, uteri, and pituitary glands were harvested for histopathological examination.

### Statistical analysis

All data are presented as mean ± standard error of mean (SEM) unless otherwise noted. Statistical significance was calculated with either two-tailed Student’s *t*-test or ANOVA (using Mann–Whitney, Kruskal–Wallis, or Tukey’s comparison test) except where otherwise stated. GraphPad Prism software version 9 (California, USA) was utilized for statistical computations. Significance was taken as *p ≤*0.05 in all cases.

## Data availability statement

The RNAseq datasets presented in this study can be found in online repositories. The name of the repository/repositories and accession number(s) can be found below: GSE237306 (GEO). The corresponding author can be contacted directly for other datasets.

## Ethics statement

The animal study was approved by National Cancer Institute (NCI) Animal Care and Use Committee (Frederick, MD). The study was conducted in accordance with the local legislation and institutional requirements.

## Author contributions

EB: Writing – review & editing, Writing – original draft, Resources, Methodology, Investigation, Formal analysis, Conceptualization. RE-C: Writing – review & editing, Methodology, Investigation. TM: Writing – review & editing, Investigation, Formal analysis. CB: Writing – review & editing, Formal analysis. AK: Writing – review & editing, Methodology, Investigation. OD: Writing – review & editing, Methodology, Investigation. JF: Writing – review & editing, Investigation. MR: Writing – review & editing, Investigation. TS: Writing – review & editing, Investigation. MH: Writing – review & editing, Investigation. MS: Writing – review & editing, Resources. VL: Writing – review & editing, Supervision, Methodology, Investigation, Formal analysis. BB: Writing – review & editing, Supervision, Formal analysis. HY: Writing – review & editing, Supervision, Funding acquisition. JV: Investigation, Writing – review & editing.
