# Supplementary material for: Aberrant CD8+T cells drive reproductive dysfunction in female mice with elevated IFN-γ levels
Source: Front Immunol. 2024 Apr 18;15:1368572. doi: 10.3389/fimmu.2024.1368572 (PMC11064017; doi:10.3389/fimmu.2024.1368572)
Supplement: Supplementary file 1 [file DataSheet_1.doc]

Supplementary Material

**Aberrant CD8^+^T cells drive reproductive dysfunction in female mice with elevated IFN-γ levels**

**Enitome E. Bafor^1*^,** **Rebecca A. Erwin-Cohen^1^, Toni Martin^1^, Clayton Baker^2,3^, Adrienne E. Kimmel^1^, Olivier Duverger^4^, John M. Fenimore^1^, Meredith Ramba^1^, Thea Spindel^1^, Megan M. Hess^1^, Baktiar Karim****^5^, Ksenia Vulikh^5^, Donna Butcher^5^, Brad Gouker^5^, Jennifer Matta^5^, Janis L. Krolus^5^**, **Michael Sanford^1^, Vanja Lazarevic^6^, Bérénice A. Benayoun^2,3^, Howard A. Young^1^, Julio C. Valencia^1^**

^1^Cancer Innovation Laboratory, Center for Cancer Research, National Cancer Institute, National Institutes of Health, Frederick, MD 21702, USA

^2^Leonard Davis School of Gerontology, University of Southern California, Los Angeles, CA 90089, USA

^3^Molecular and Computational Biology Department, University of Southern California, Dornsife College of Letters, Arts and Sciences, Los Angeles, CA 90089, USA

^4^Craniofacial Anomalies and Regeneration Section National Institute of Dental and Craniofacial Research, National Institutes of Health, Bethesda, MD 20892, USA

^5^Frederick National Laboratory for Cancer Research, National Cancer Institute, Frederick, MD 21702, USA

^6^Experimental Immunology Branch, Center for Cancer Research, National Cancer Institute, National Institutes of Health, Bethesda, MD 20892 USA

***Correspondence:**

Enitome E. Bafor

enitome.bafor@nih.gov

**Keywords: interferon-gamma (IFN-γ), CD8^+^ T cells, hypophysitis, tissue-resident memory, luteinization defect, prolactin deficiency, implantation failure, pregnancy.**

**Supplementary Results**

**
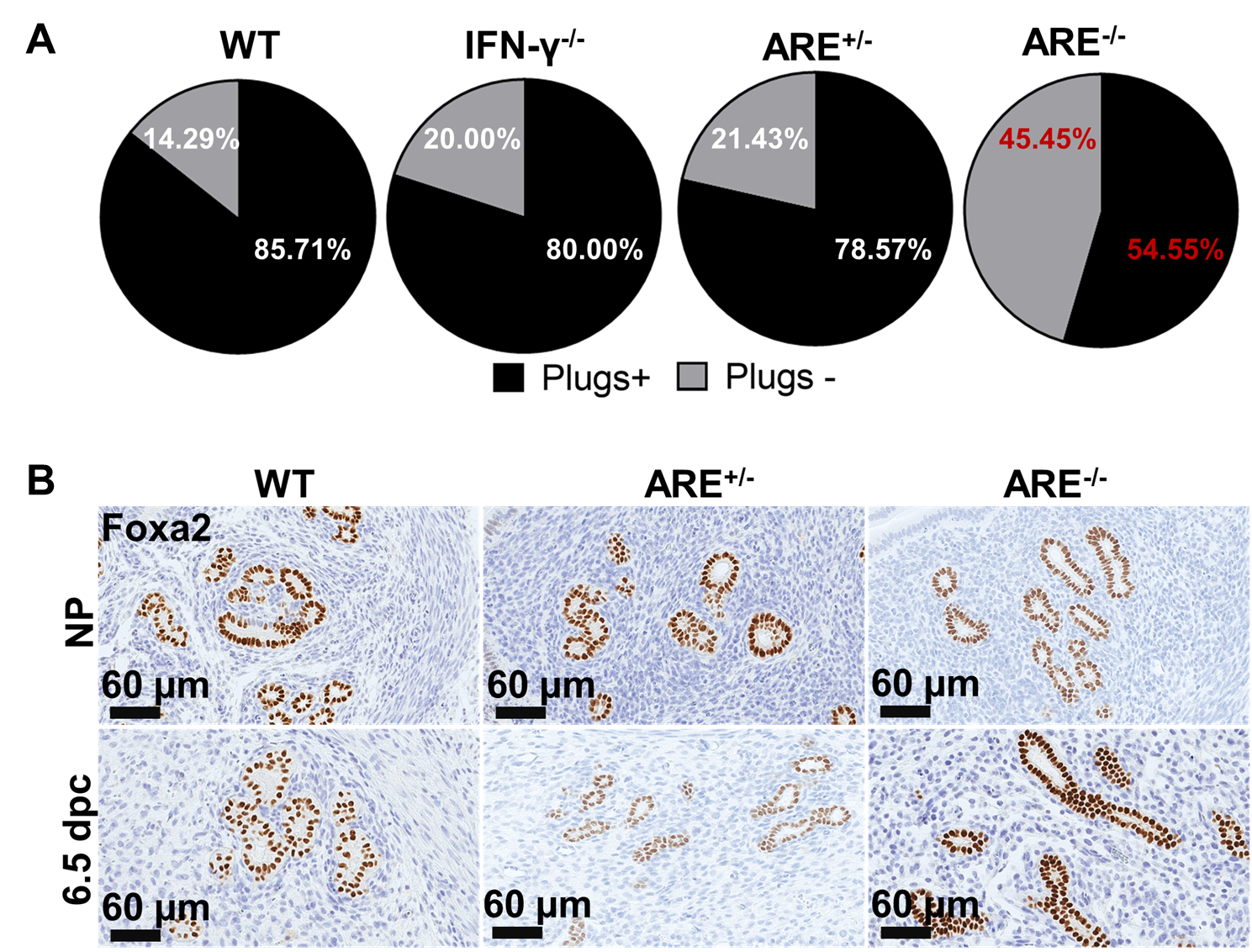
**

**Supplementary Figure 1. ARE^-/-^ mice showed normal mating behavior and similar Foxa2 expression pattern in the uterus. (A**) Percentage of WT, IFN-γ^-/-^, ARE^+/-^, and ARE^-/-^ females with copulatory plugs after pairing with fertile WT males (n = 11-15 mice) (pooled data from two independent experiments). (**B**) Representative IHC images showing qualitative Foxa2 expression (black arrows) in the non-pregnant (NP) and 6.5 dpc uteri (n = 3-4 mice/group). All experiments were performed two independent times, data represent mean ± SEM, and ‘n’ denotes animals per group.

**
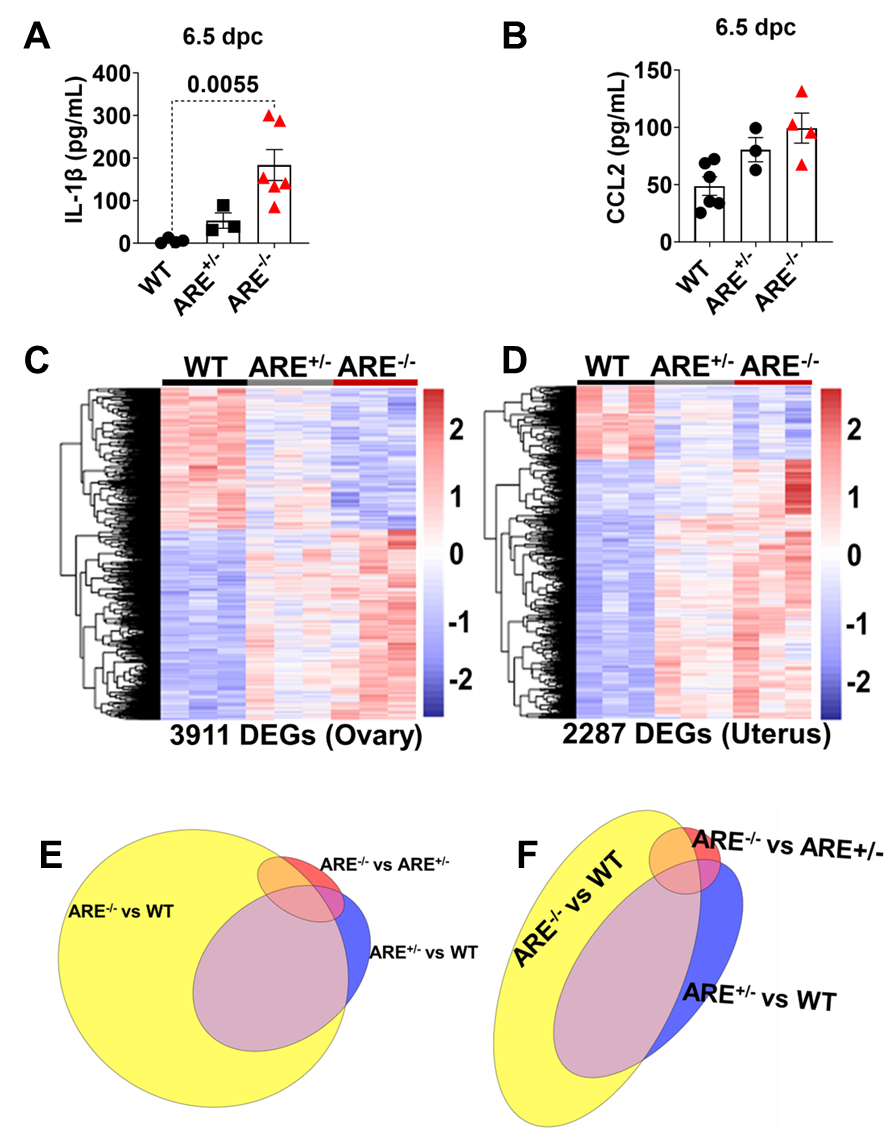
**

**Supplementary Figure 2. Elevated immune activation profiles in ARE^-/-^ mice.** Plasma levels of **(A)** IL-1β and **(B)** CCL2 in WT, ARE^+/-^ and ARE^-/-^ mice (n = 3-6); Data represent mean ± SEM from two independent experiments); Statistical significance, one-way ANOVA with Kruskal-Wallis test; (**C-D**) Heatmaps of normalized significant genes differentially expressed between WT and ARE^-/-^ mouse ovary and uterus (n = 3, FDR <5%). (**E-F**) Venn diagrams displaying overlap of genes differentially expressed (FDR <5%) between (**E**) ARE^-/-^ and WT ovaries (3911 genes), ARE^-/-^ and ARE^+/-^ ovaries (185 genes), and ARE^+/-^ and WT ovaries (1339 genes) (n = 3). (**F**) ARE^-/-^ and ARE^+/-^ uteri (179 genes), ARE^+/-^ and WT uteri (1298 genes) and ARE^-/-^ and WT uteri (2287 genes) (n = 3 mice). ‘n’ denotes number of animals per group in all cases.


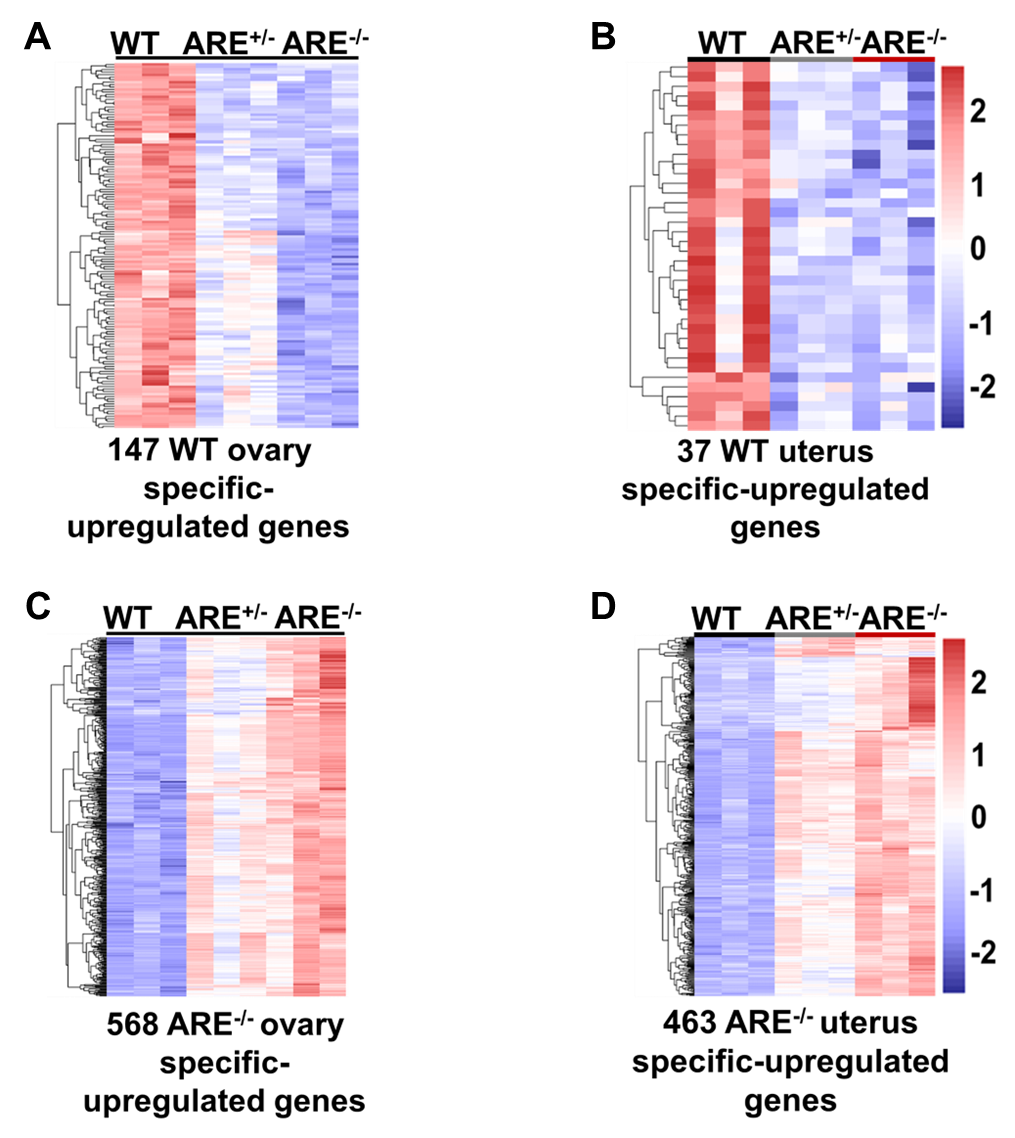


**Supplementary Figure 3**. **Differentially expressed genes identified by RNAseq in WT, ARE^+/-^ and ARE^-/-^ mouse ovary and uterus**. (**A**) Heatmaps of normalized expression of genes identified through the likelihood-ratio test (LRT) analysis as upregulated in (**A**) WT ovary, (**B**) WT uterus, (**C**) ARE^-/-^ ovary, and (**D**) ARE^-/-^ uterus (n = 3 mice/genotype, FDR <5%). ‘n’ denotes number of animals per group in all cases.

**Supplementary Table 1. Genes related to T-cell activation in the ovary.**

| Gene Name | ENSEMBL ID | LRT adj  p-value | ARE^+/-^ vs WT  pairwise log2fc | ARE^-/-^ vs WT pairwise log2fc |
| --- | --- | --- | --- | --- |
| *Ifng* | ENSMUSG00000055170 | 2.77E-05 | 2.767301794 | 4.090394544 |
| *Aw112010* | ENSMUSG00000075010 | 2.12E-24 | 2.681753584 | 3.784109191 |
| *Cd27* | ENSMUSG00000030336 | 1.32E-05 | 1.619331436 | 2.697894178 |
| *Cd4* | ENSMUSG00000023274 | 1.33E-05 | 0.795399989 | 1.99076531 |
| *Cd3g* | ENSMUSG00000002033 | 7.95E-09 | 1.96674671 | 3.072393806 |
| *Cd3e* | ENSMUSG00000032093 | 6.42E-09 | 1.78247636 | 2.990406432 |
| *Cd3d* | ENSMUSG00000032094 | 4.99E-10 | 2.011258307 | 3.425116584 |
| *Cd6* | ENSMUSG00000024670 | 1.25E-07 | 1.945395417 | 3.379234257 |
| *Trbc2* | ENSMUSG00000076498 | 9.51E-16 | 2.409838221 | 3.377470671 |
| *Spn* | ENSMUSG00000051457 | 7.92E-11 | 2.076681986 | 2.619136898 |
| *Snx20* | ENSMUSG00000031662 | 4.55E-10 | 2.018371015 | 2.56969341 |
| *Slamf9* | ENSMUSG00000026548 | 7.96E-06 | 2.388046844 | 2.571362642 |
| *Slamf8* | ENSMUSG00000053318 | 2.53E-20 | 2.376206602 | 3.384001009 |
| *Lat* | ENSMUSG00000030742 | 4.14E-05 | 0.608235204 | 1.690757245 |
| *Aif1* | ENSMUSG00000024397 | 7.27E-13 | 1.722616795 | 2.500676871 |
| *Bcl11b* | ENSMUSG00000048251 | 7.20E-05 | 1.566581611 | 2.153782549 |
| *Tbx21* | ENSMUSG00000001444 | 1.63E-08 | 2.261158569 | 3.058666436 |
| *Eomes* | ENSMUSG00000032446 | 0.001E-06 | 1.764330547 | 2.49123928 |
| *Cd69* | ENSMUSG00000030156 | 2.22E-05 | 2.864734453 | 3.78087064 |
| *Calhm6* | ENSMUSG00000046031 | 1.83E-19 | 2.330020053 | 3.270317166 |
| *Ccdc88b* | ENSMUSG00000047810 | 4.53E-16 | 1.801674043 | 2.292429747 |
| *Ccl19* | ENSMUSG00000071005 | 2.63E-06 | 2.310569223 | 3.37415431 |
| *Cd96* | ENSMUSG00000022657 | 3.63E-06 | 1.872200696 | 2.778186714 |
| *Ccl22* | ENSMUSG00000031779 | 4.08E-08 | 1.488271048 | 2.219149811 |
| *Fyb* | ENSMUSG00000022148 | 3.92E-08 | 1.918724694 | 2.191354087 |
| *Hcls1* | ENSMUSG00000022831 | 5.27E-08 | 1.801125065 | 2.152323819 |
| *Itk* | ENSMUSG00000020395 | 5.24E-06 | 1.039116765 | 2.034769329 |
| *Lck* | ENSMUSG00000000409 | 2.77E-08 | 0.973182165 | 2.010541477 |
| *Lcp1* | ENSMUSG00000021998 | 7.99E-06 | 1.26228228 | 1.597957758 |
| *Lcp2* | ENSMUSG00000002699 | 3.20E-07 | 2.073863298 | 2.202455119 |
| *Pglyrp1* | ENSMUSG00000030413 | 3.23E-07 | 1.556549402 | 1.557579018 |
| *Pglyrp2* | ENSMUSG00000079563 | 4.91E-07 | 1.629936239 | 2.922914821 |
| *Trbv3* | ENSMUSG00000076463 | 5.85E-05 | 3.40645348 | 5.707898314 |
| *Skap1* | ENSMUSG00000057058 | 2.82E-05 | 1.249018362 | 2.022336684 |

**Supplementary Table 2. Genes related to T-cell activation in the uterus.**

| Gene Name | ENSEMBL ID | LRT adj  p-value | ARE^+/-^ vs WT  pairwise log2fc | ARE^-/-^ vs WT pairwise log2fc |
| --- | --- | --- | --- | --- |
| *Cd3d* | ENSMUSG00000032094 | 5.22E-23 | 2.394768695 | 4.117358796 |
| *Cd3g* | ENSMUSG00000002033 | 1.47E-19 | 0.825129883 | 2.347788641 |
| *Cd3e* | ENSMUSG00000032093 | 2.49E-16 | 1.313163294 | 2.865161452 |
| *Cd4* | ENSMUSG00000023274 | 1.25E-06 | 1.239690438 | 2.38646043 |
| *Ifng* | ENSMUSG00000055170 | 1.99E-09 | 4.049644156 | 4.97848316 |
| *Tcra* | ENSMUSG00000076928 | 2.29E-20 | 2.255550956 | 3.832641485 |
| *Tcrb* | ENSMUSG00000076490 | 0.000119212 | 0.788793238 | 1.411413306 |
| *Trbc2* | ENSMUSG00000076498 | 3.15E-16 | 0.988195237 | 2.421731239 |
| *Tcrg* | ENSMUSG00000076754 | 0.140538973 | 1.243075704 | 1.769611945 |
| *Trgc2* | ENSMUSG00000076752 | 5.01E-07 | 1.010980483 | 1.82085279 |
| *Tcrd* | ENSMUSG00000104876 | 0.40552542 | 0.131144558 | 1.113017147 |
| *Tbet* | ENSMUSG00000001444 | 0.008105284 | 0.866975401 | 1.497016318 |
| *Eomes* | ENSMUSG00000032446 | 0.788314205 | 0.36479053 | 0.540487313 |
| *Il2* | ENSMUSG00000027720 | 0.356174445 | 1.128653515 | 2.409807946 |
| *Zc3h12d* | ENSMUSG00000039981 | 3.76E-05 | 1.885741084 | 2.409403616 |
| *Xcr1* | ENSMUSG00000060509 | 8.12E-05 | 1.63127381 | 2.145768106 |
| *Trat1* | ENSMUSG00000030775 | 6.11E-05 | 1.992283771 | 3.388003213 |
| *Spn* | ENSMUSG00000051457 | 2.68E-05 | 1.4025929 | 1.867005882 |
| *Snx20* | ENSMUSG00000031662 | 3.35E-06 | 1.81451916 | 2.130064884 |
| *Slamf9* | ENSMUSG00000026548 | 5.77E-06 | 2.542907862 | 2.347808284 |
| *Slamf8* | ENSMUSG00000053318 | 2.49E-16 | 1.919914086 | 2.739750372 |
| *Adam8* | ENSMUSG00000025473 | 1.58E-05 | 1.246771928 | 1.110211521 |
| *Aw112010* | ENSMUSG00000075010 | 9.41E-13 | 1.921353959 | 3.085707512 |
| *Cd27* | ENSMUSG00000030336 | 1.26E-09 | 1.843912987 | 2.981144397 |
| *Cd6* | ENSMUSG00000024670 | 2.26E-21 | 2.640290994 | 4.440288235 |
| *Ebi3* | ENSMUSG00000003206 | 8.25E-12 | 1.902862465 | 2.387534245 |
| *Icos* | ENSMUSG00000026009 | 3.91E-06 | 0.842282428 | 2.482547557 |
| *Lat* | ENSMUSG00000030742 | 2.86E-18 | 1.457455347 | 2.44157366 |


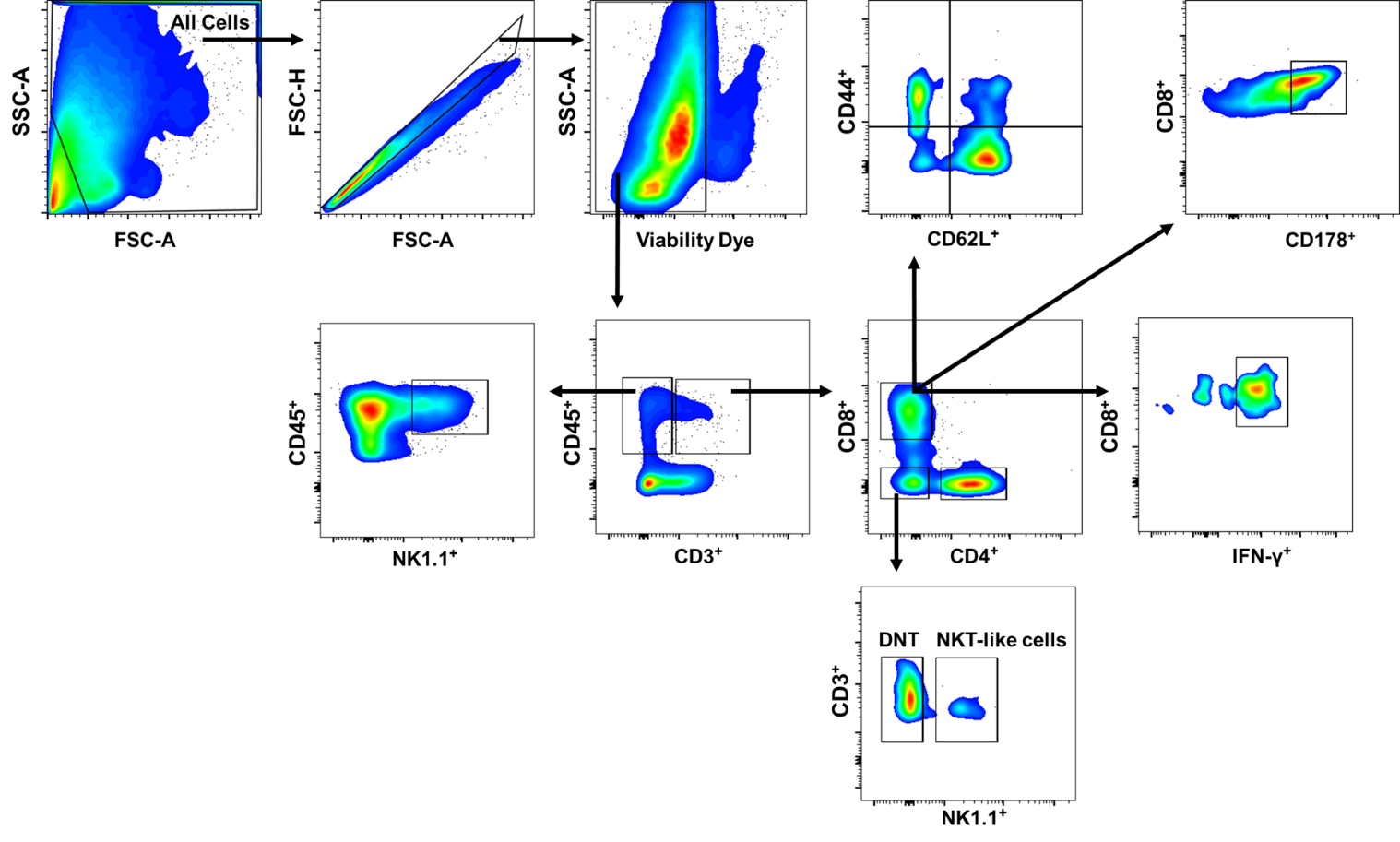


**Supplementary Figure 4**. Representative plots showing the gating strategy for immune cells in the ovary and uterus; Lysed cells (≤ 50K on FSC-A) and doublets (FSC-H vs FSC-A) were excluded from cells; Live cells were gated based on exclusion of positive staining Zombie Aqua cells; CD3^+^T cells were selected from live CD45^+^ cells; CD8^+^ and CD4^+^ T cells were gated from the CD3^+^ population and NK cells were gated from CD3^-^ cells.

**
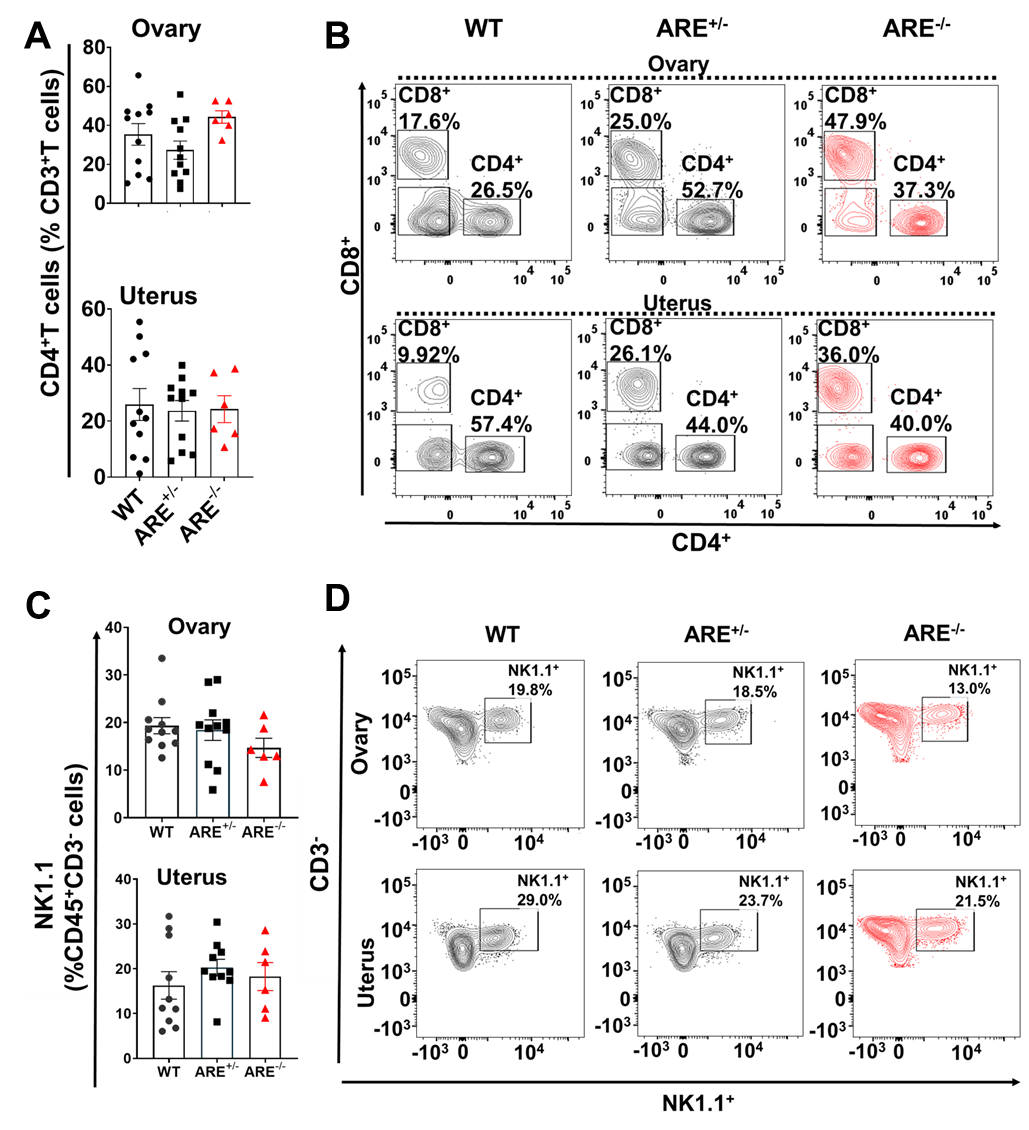
**

**Supplementary Figure 5. CD8^+^T cells increased in ARE^-/-^ mouse ovary and uterus. (A)** Flow cytometry analysis of CD4^+^T cells in WT, ARE^+/-^ and ARE^-/-^ mice (n = 6-11). (**B**) Representative flow cytometry density plots of CD8^+^ and CD4^+^T cell frequencies in ARE^-/-^ mouse ovary and uterus. (**C**) Flow cytometry analysis of NK1.1^+^ cells in WT, ARE^+/-^ and ARE^-/-^ mice (n = 6-10). (**D**) Representative flow cytometry plots of NK1.1^+^ cell frequencies in ARE^-/-^ mouse ovary and uterus. All experiments were performed three independent times. Statistical significance, one-way ANOVA with Kruskal-Wallis test; data represent mean ± SEM, and ‘n’ denotes animals per group.


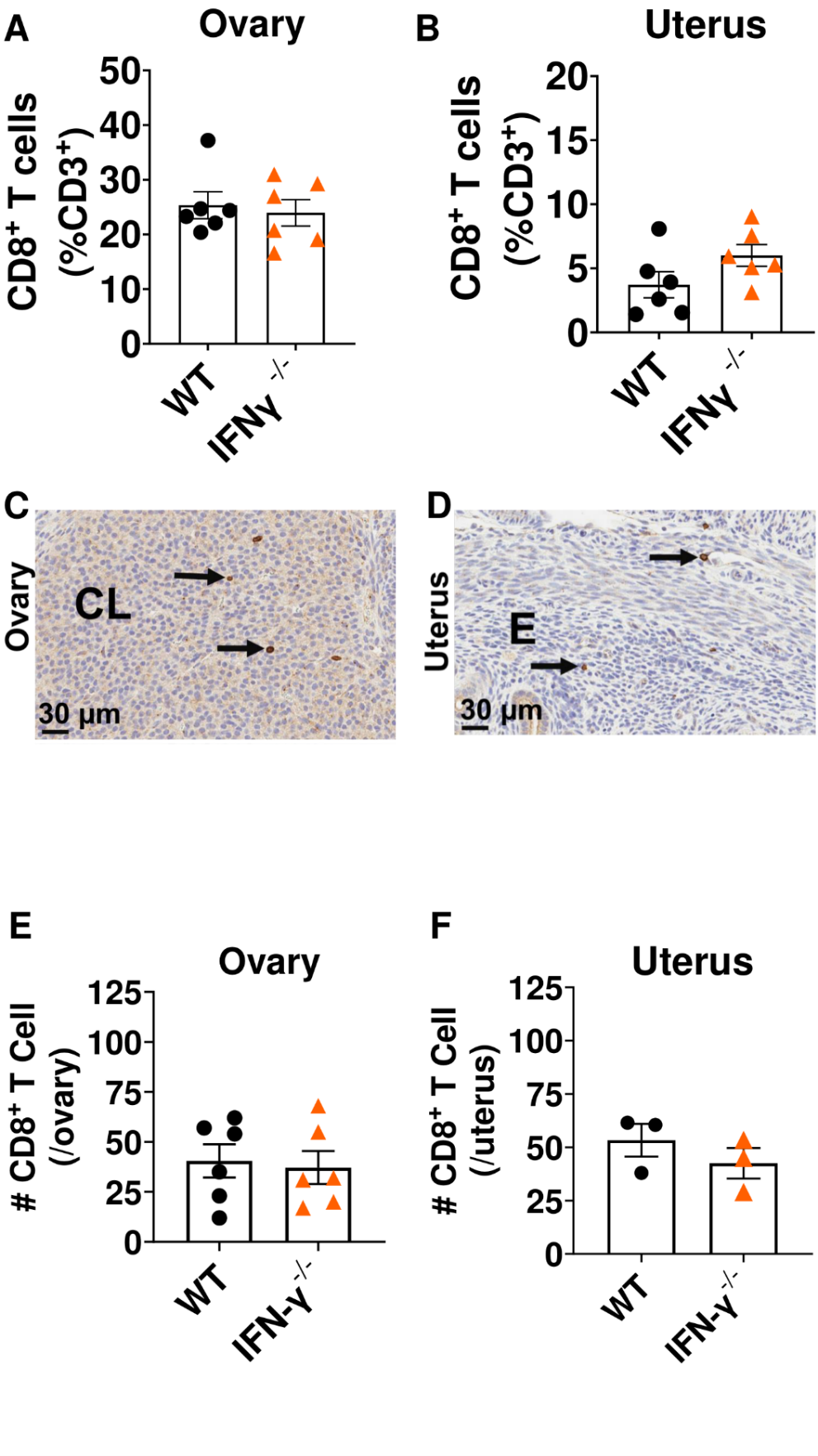


**Supplementary Figure 6. No significant difference in CD8^+^T cells in IFN-γ^-^/^-^ mouse ovary and uterus. (A-B)** Flow cytometry analysis of CD8^+^T cells in non-pregnant IFN-γ^-/-^ mouse ovary and uterus (n = 6) compared to WT cohorts. (**C-D**) Representative IHC images showing the distribution of CD8a positive cells (black arrows) in non-pregnant IFN-γ^-/-^ mouse ovary and uterus, CL = corpus luteum; E = endometrium. Black arrows = CD8a^+^ T cells. (**E-F**) Number of CD8a^+^ T cells within the ovary (two ovary sections per mouse) and uterus (per mm^2^) in non-pregnant IFN-γ^-/-^ mice (n= 3). All experiments were performed two independent times. Statistical significance, two-tailed student t-test with Mann-Whitney analysis; data represent mean ± SEM, and ‘n’ denotes animals per group.

**
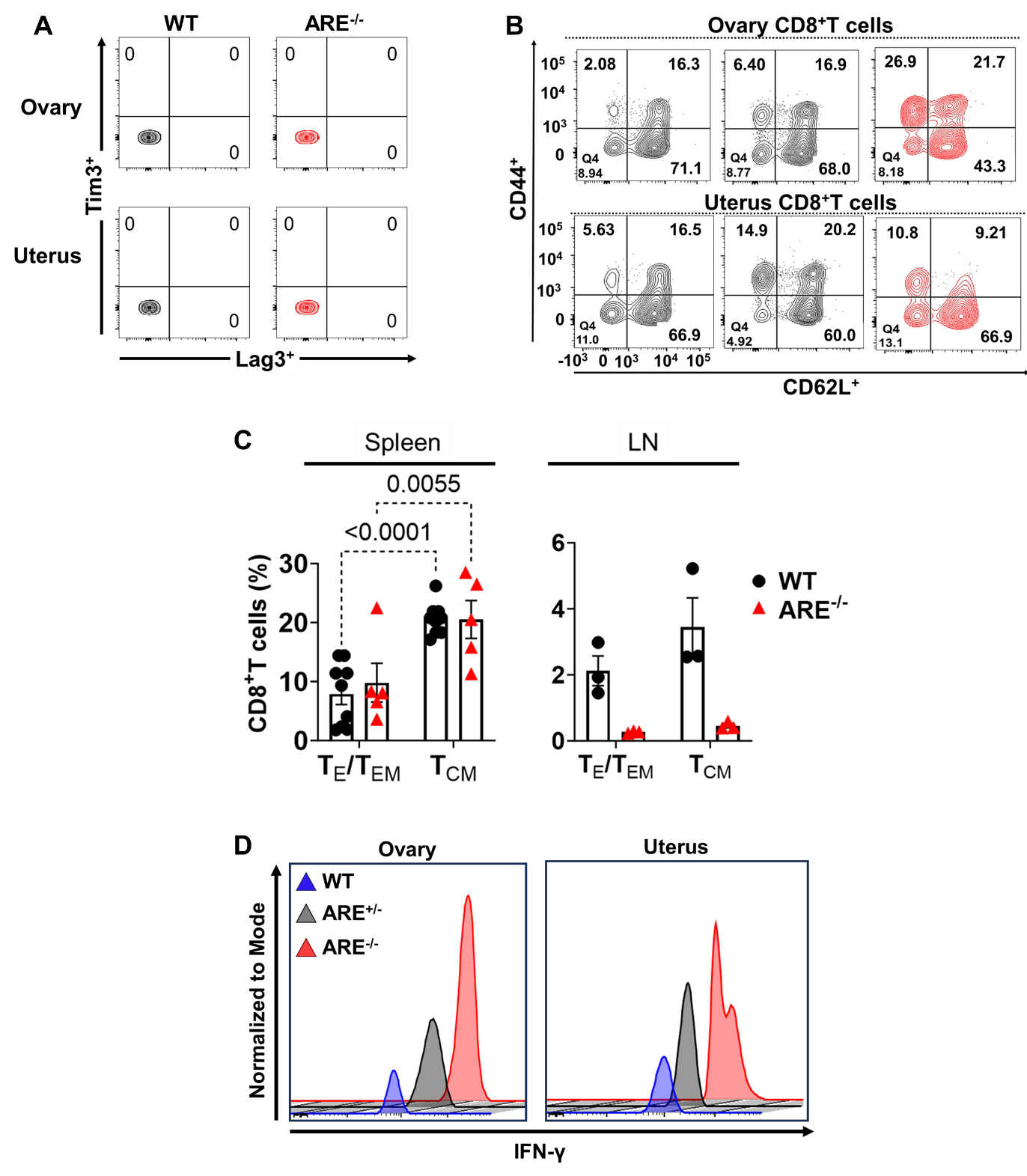
**

**Supplementary Figure 7. Effector/Effector memory CD8^+^T cells are dominant in the ARE^-/-^ mouse ovary and uterus.** Representative flow cytometry density plots of the ovary and uterus showing (**A**) Tim-3 and Lag-3 expression on CD8^+^T cells (n=3), (**B**) Effector/effector memory (T_E/EM_) and central memory (T_CM_) CD8^+^ T cells in the ovary and uterus identified using CD44 and CD62L (n= 5-9). (**C**) CD8^+^ T_E/EM_ and CD8^+^ T_CM_ cells in the spleen and lymph nodes draining the ovary and uterus; LN = lymph node (n= 3-9). (**D**) Representative flow cytometry histograms of CD8^+^T cell IFN-γ expression in the ovary and uterus (n= 4-6); blue = WT, black = ARE^+/-^, and Red = ARE^-/-^. CD69 and CD49a expression on CD8^+^T cells. All experiments were performed three independent times. Statistical significance, two-way ANOVA with Tukey’s test; data represent mean ± SEM, and ‘n’ denotes animals per group.

**Supplementary Table 3. Genes related to CD8^+^T-cell activation in the ovary.**

| Gene Name | ENSEMBL ID | LRT adj  p-value | ARE^+/-^ v WT pairwise log2fc | ARE^-/-^ v WT pairwise log2fc |
| --- | --- | --- | --- | --- |
| *Cd8a* | ENSMUSG00000053977 | 6.69E-29 | 2.642050577 | 4.044199729 |
| *Cd8b1* | ENSMUSG00000053044 | 1.04E-16 | 2.009138096 | 3.731184423 |
| *Cd2* | ENSMUSG00000027863 | 2.97E-10 | 2.592238267 | 3.746760889 |
| *Tapbpl* | ENSMUSG00000038213 | 1.04E-21 | 0.907061531 | 1.569080457 |
| *Tap2* | ENSMUSG00000024339 | 2.06E-27 | 0.824182169 | 1.379802156 |
| *Tap1* | ENSMUSG00000037321 | 2.58E-111 | 2.223381562 | 3.128258844 |
| *B2m* | ENSMUSG00000060802 | 5.86E-37 | 1.801953871 | 2.733604356 |
| *Gzmk* | ENSMUSG00000042385 | 1.00E-07 | 3.620652207 | 4.6362268 |
| *Gzmb* | ENSMUSG00000015437 | 1.70E-06 | 2.144286011 | 3.024775806 |
| *Grn* | ENSMUSG00000034708 | 5.92E-05 | 0.610574573 | 0.851906037 |
| *Gsdmd* | ENSMUSG00000022575 | 6.00E-11 | 0.586973924 | 1.195252689 |
| *Prf1* | ENSMUSG00000037202 | 7.92E-09 | 2.503648767 | 3.287335803 |
| *Trex1* | ENSMUSG00000049734 | 4.76E-07 | 0.626339719 | 0.894538277 |
| *Laptm5* | ENSMUSG00000028581 | 1.13E-07 | 1.43951513 | 1.999733883 |
| *Ccl5* | ENSMUSG00000035042 | 3.12E-26 | 2.93307676 | 4.358135286 |
| *Klrk1* | ENSMUSG00000030149 | 1.46E-08 | 1.729250353 | 2.37546803 |
| *Map4k1* | ENSMUSG00000037337 | 4.01E-05 | 0.892136872 | 1.426704312 |
| *Nkg7* | ENSMUSG00000004612 | 1.15E-23 | 2.37931143 | 3.713582363 |
| *Psmb10* | ENSMUSG00000031897 | 6.39E-30 | 1.127246378 | 1.794339842 |
| *Psmb8* | ENSMUSG00000024338 | 4.35E-73 | 2.194266548 | 3.165650453 |
| *Psmb9* | ENSMUSG00000096727 | 9.01E-92 | 2.463272064 | 3.429174292 |
| *Psme2* | ENSMUSG00000079197 | 1.78E-11 | 0.476661976 | 0.780939425 |
| *Batf2* | ENSMUSG00000039699 | 1.32E-33 | 2.312604919 | 3.481333766 |
| *Fasl* | ENSMUSG00000000817 | 0.12E-07 | 1.44752992 | 1.790701394 |

**Supplementary Table 4. Genes related to CD8^+^T-cell activation in the uterus.**

| Gene Name | ENSEMBL ID | LRT adj  p-value | | ARE^+/-^ v WT pairwise log2fc | ARE^-/-^ v WT pairwise log2fc |
| --- | --- | --- | --- | --- | --- |
| *Cd8a* | ENSMUSG00000053977 | 2.77E-42 | 3.506841729 | | 5.354396887 |
| *Cd8b1* | ENSMUSG00000053044 | 1.51E-24 | 3.464969776 | | 5.185244326 |
| *Zap70* | ENSMUSG00000026117 | 7.10E-07 | 0.609741491 | | 1.627934124 |
| *Tapbpl* | ENSMUSG00000038213 | 5.59E-05 | 0.936960963 | | 1.262240975 |
| *Tap2* | ENSMUSG00000024339 | 9.75E-08 | 1.068152936 | | 1.66465527 |
| *Tap1* | ENSMUSG00000037321 | 8.46E-21 | 2.219551437 | | 2.725434537 |
| *B2m* | ENSMUSG00000060802 | 7.75E-16 | 2.083013523 | | 2.537919498 |
| *Batf3* | ENSMUSG00000026630 | 2.55E-07 | 1.581597111 | | 1.719360821 |
| *Ccl5* | ENSMUSG00000035042 | 1.70E-08 | 1.352425811 | | 2.513804276 |
| *Flt3l* | ENSMUSG00000110206 | 4.97E-06 | 1.064407999 | | 1.560838557 |
| *Gzma* | ENSMUSG00000023132 | 3.75E-05 | 0.81962506 | | 1.904800233 |
| *Gzmk* | ENSMUSG00000042385 | 1.48E-16 | 3.108502619 | | 5.686275271 |
| *Gzmb* | ENSMUSG00000015437 | 0.95E-07 | 0.219788052 | | -0.153170407 |
| *Perforin* | ENSMUSG00000037202 | 0.89E-07 | 0.436251807 | | 0.359067723 |
| *Fasl* | ENSMUSG00000000817 | 0.27E-07 | 1.149768619 | | 1.348074819 |

**
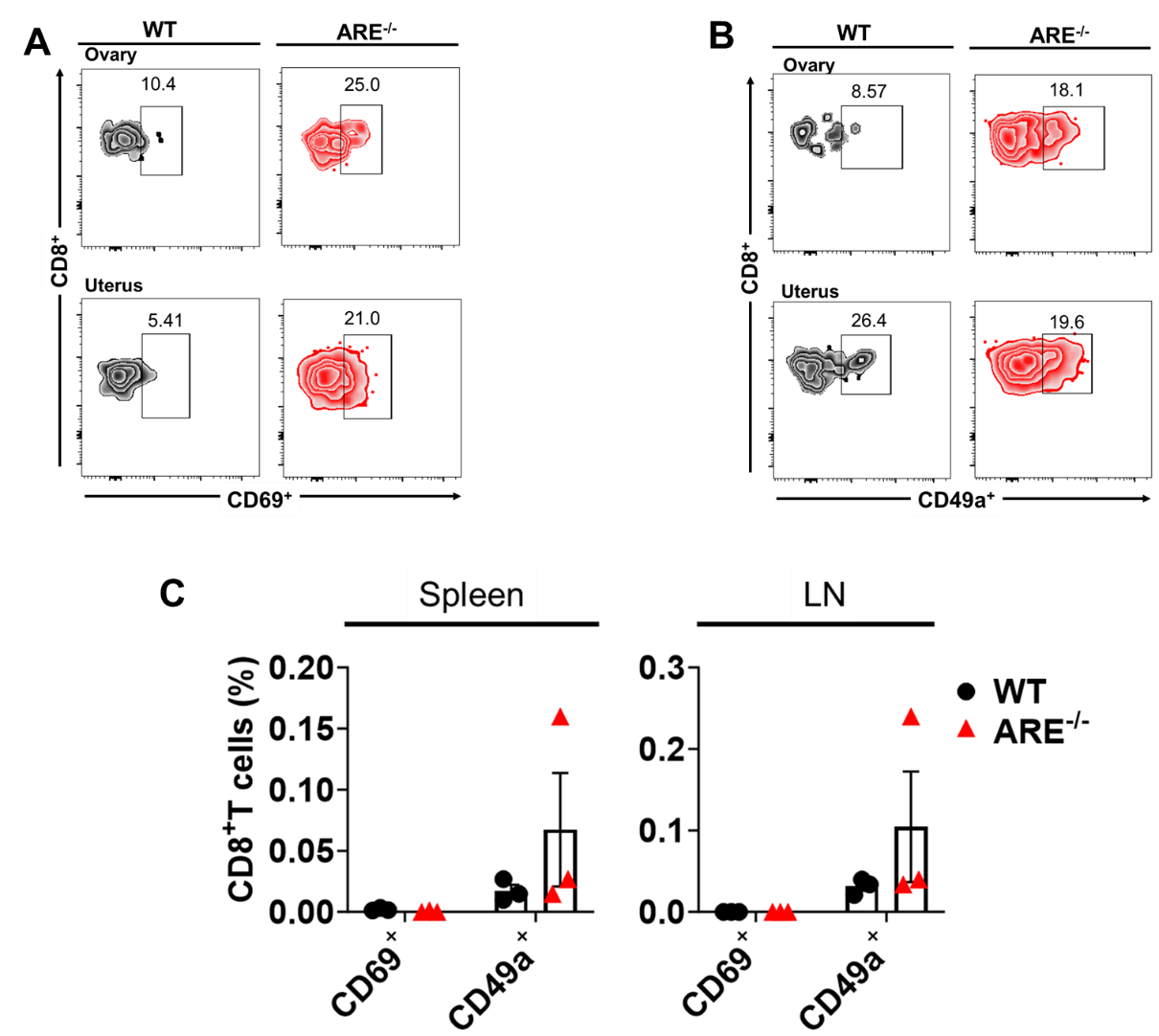
**

**Supplementary Figure 8. CD69 and CD49a expression on CD8^+^T cells are increased in the ARE^-/-^ mouse ovary and uterus.** Representative flow cytometry density plots of the ovary and uterus showing (**A**) CD69 expression and (**B**) CD49a expression on CD8^+^ T cells (n = 3-5). (**C**) CD69 and CD49a expressions on CD8^+^T cells in the spleen and lymph nodes draining the ovary and uterus; LN = lymph node (n= 3). All experiments were performed two independent times. Statistical significance, two-way ANOVA with Tukey’s test; data represent mean ± SEM, and ‘n’ denotes animals per group.

**
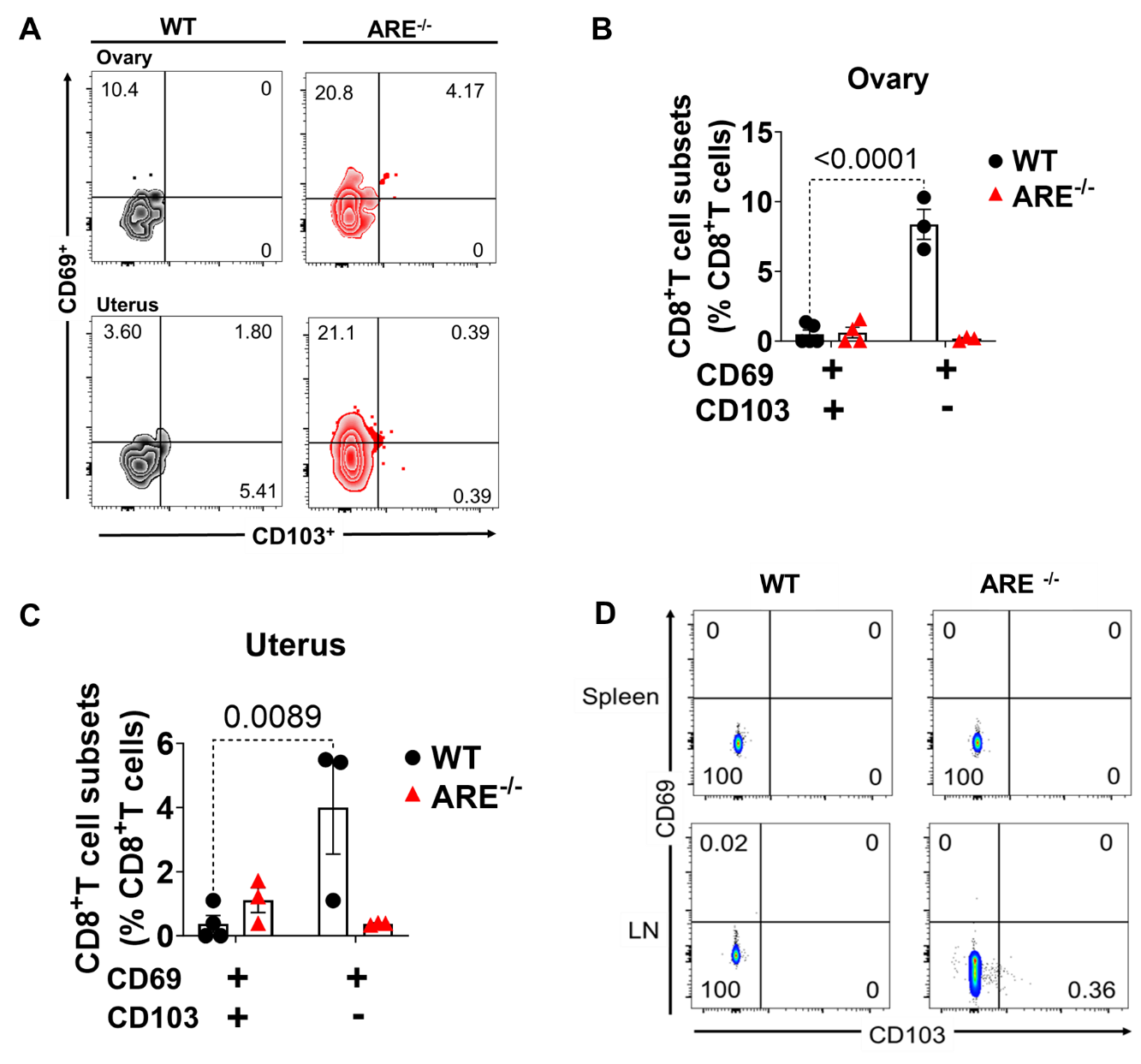
**

**Supplementary Figure 9. CD8^+^T cells co-expressing CD69 and CD103 are low in the ovary and uterus.** Representative flow cytometry density plots of the ovary and uterus showing (**A**) CD69 and CD103 expression on CD8^+^T cells. (**B-C**) CD69^+^CD103^-^ CD8^+^T cells are decreased in the ARE^-/-^ mouse (**B**) ovary and (**C**) uterus (n = 3-4). (**D**) Representative flow cytometry density plots showing CD69 and CD103 expression on CD8^+^T cells in the spleen and lymph nodes draining the ovary and uterus; LN = lymph node (n= 3). All experiments were performed two independent times. Statistical significance, two-way ANOVA with Tukey’s test; data represent mean ± SEM, and ‘n’ denotes animals per group.

**
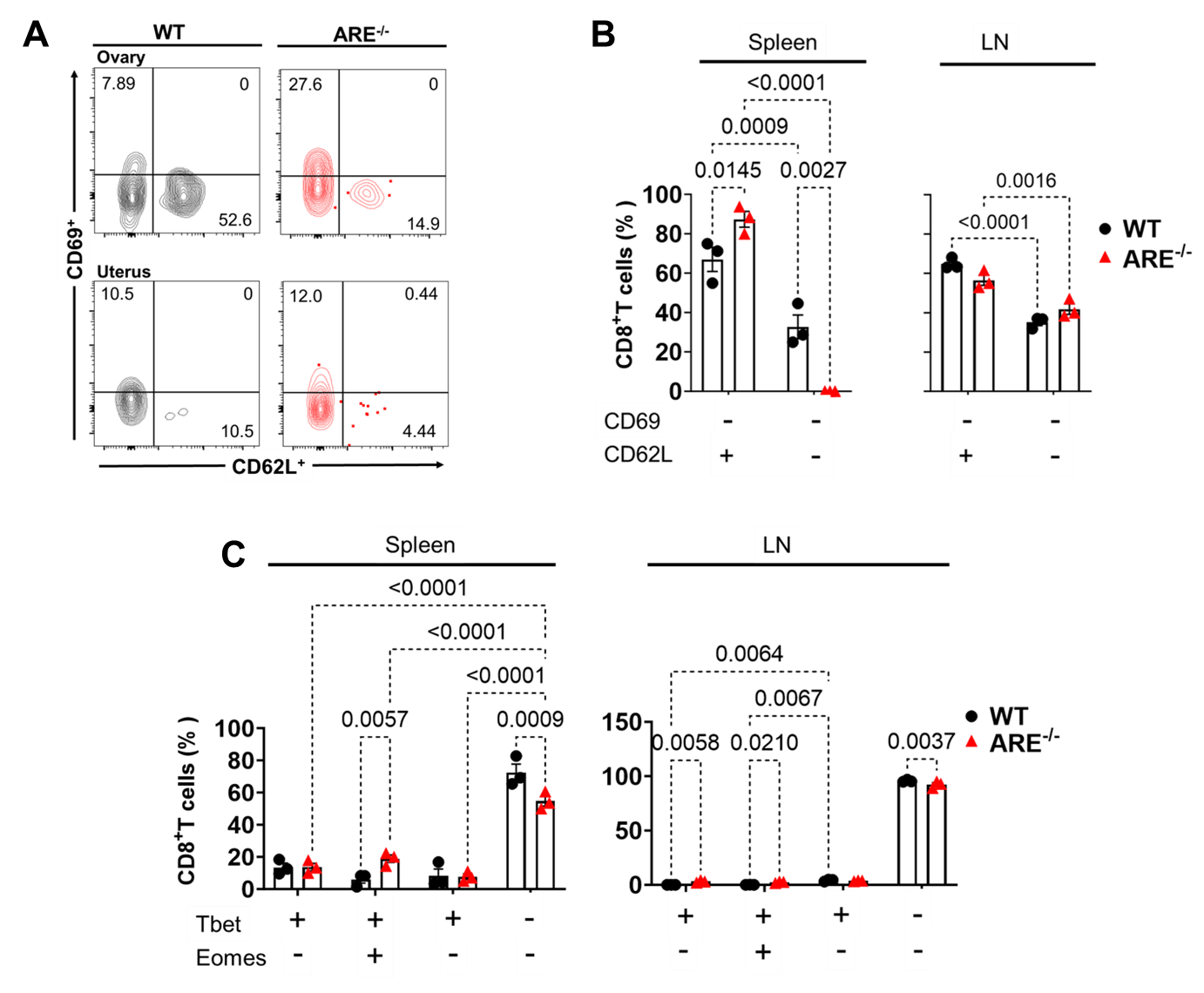
**

**Supplementary Figure 10. CD8^+^T cells negative for CD69 and CD62L expression are dominant in the ovary and uterus. (A)** Representative flow cytometry density plots of the ovary and uterus showing CD69 and CD62L expression on CD8^+^T cells (n = 3-5). (**B**) CD69^-^CD62L^+^ CD8^+^T cells are dominant in ARE^-/-^ mouse spleen and lymph nodes draining the ovary and uterus; LN = lymph node (n= 3). (**C**) CD8^+^T cells negative for Tbet and Eomes are dominant in the spleen and lymph nodes draining the ovary and uterus; LN = lymph node (n= 3). All experiments were performed two independent times. Statistical significance, two-way ANOVA with Tukey’s test; data represent mean ± SEM, and ‘n’ denotes animals per group.


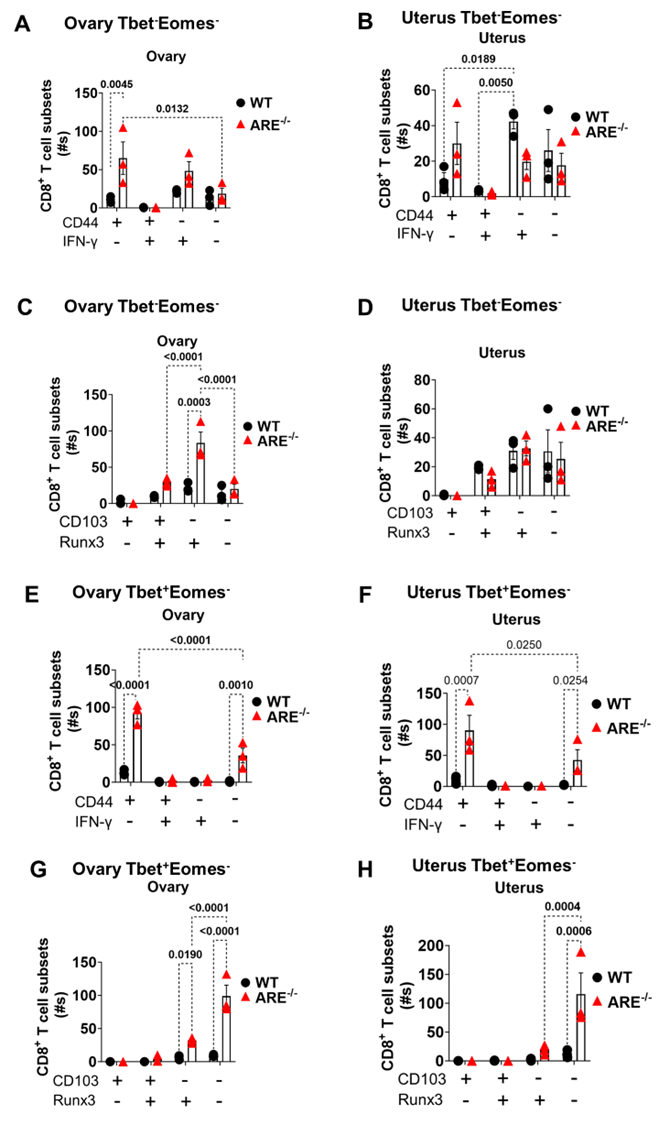


**Supplementary Figure 11. Analysis of T_EM_ and T_RM_ subsets of CD8^+^T cells in the ovary and uterus based on Tbet^-^Eomes^-^ and Tbet^+^Eomes^-^ expression. (A-D)** CD44^+^CD103^+^Runx3^+^ expression, increased in Tbet^-^Eomes^-^CD8^+^T cells in the ovary and uterus (n = 3). (**E-H**) CD44^+^ CD103^-^Runx3^-^ expression increased in Tbet^+^Eomes^-^CD8^+^T cells in the ovary and uterus (n = 3). All experiments were performed two independent times. Statistical significance, two-way ANOVA with Tukey’s test; data represent mean ± SEM, and ‘n’ denotes animals per group.


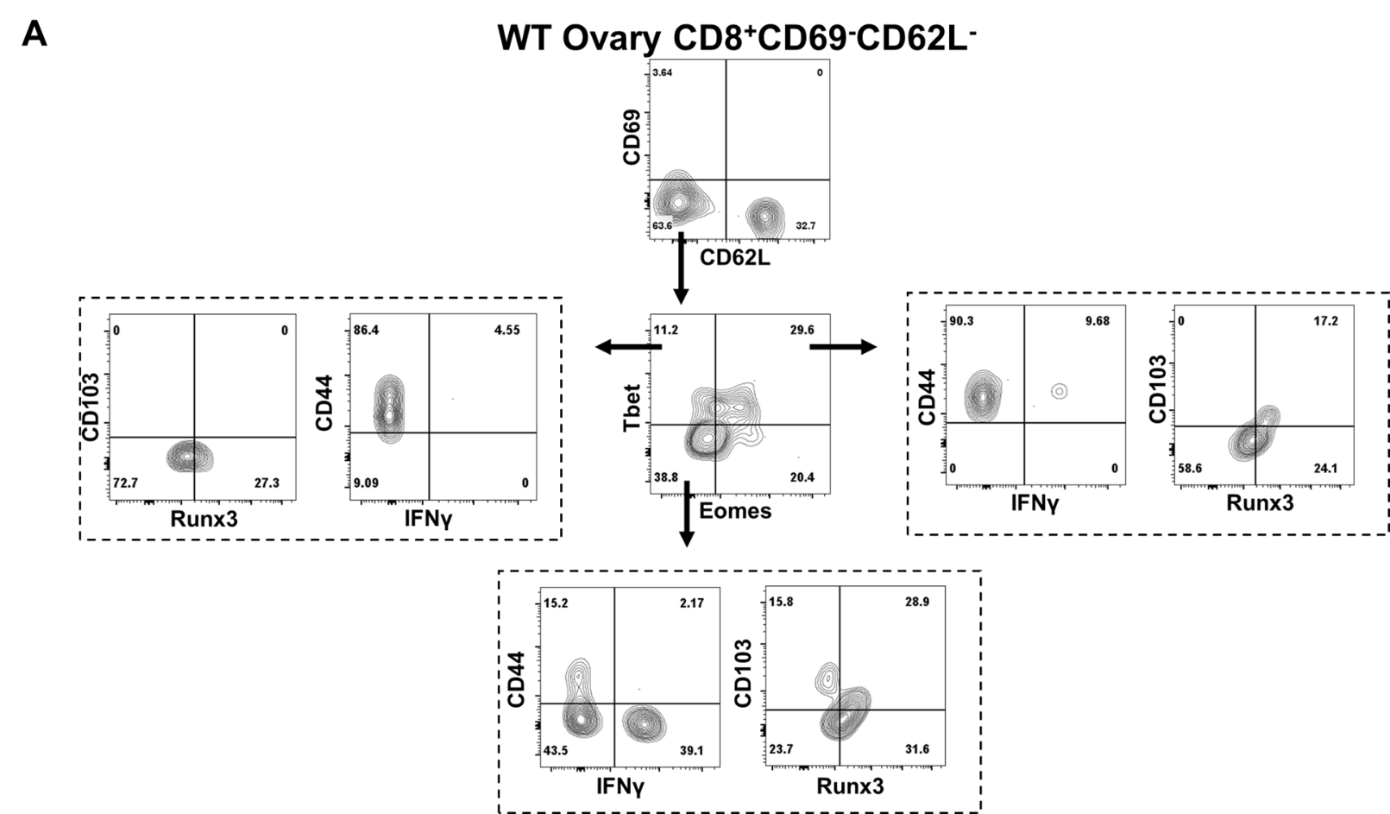


**Supplementary Figure 12. Analysis of CD8^+^CD69^-^CD62L^-^ T cells based on Tbet and Eomes, expression in WT mouse ovary.** (**A**) Representative flow cytometry plots showing frequencies and phenotypes of CD69^-^CD62L^-^CD8^+^T cells based on Tbet, Eomes, CD44, IFN-γ, Runx3, and CD103 expression in WT mouse ovary (n = 3). All experiments were performed two independent times, and ‘n’ denotes animals per group.


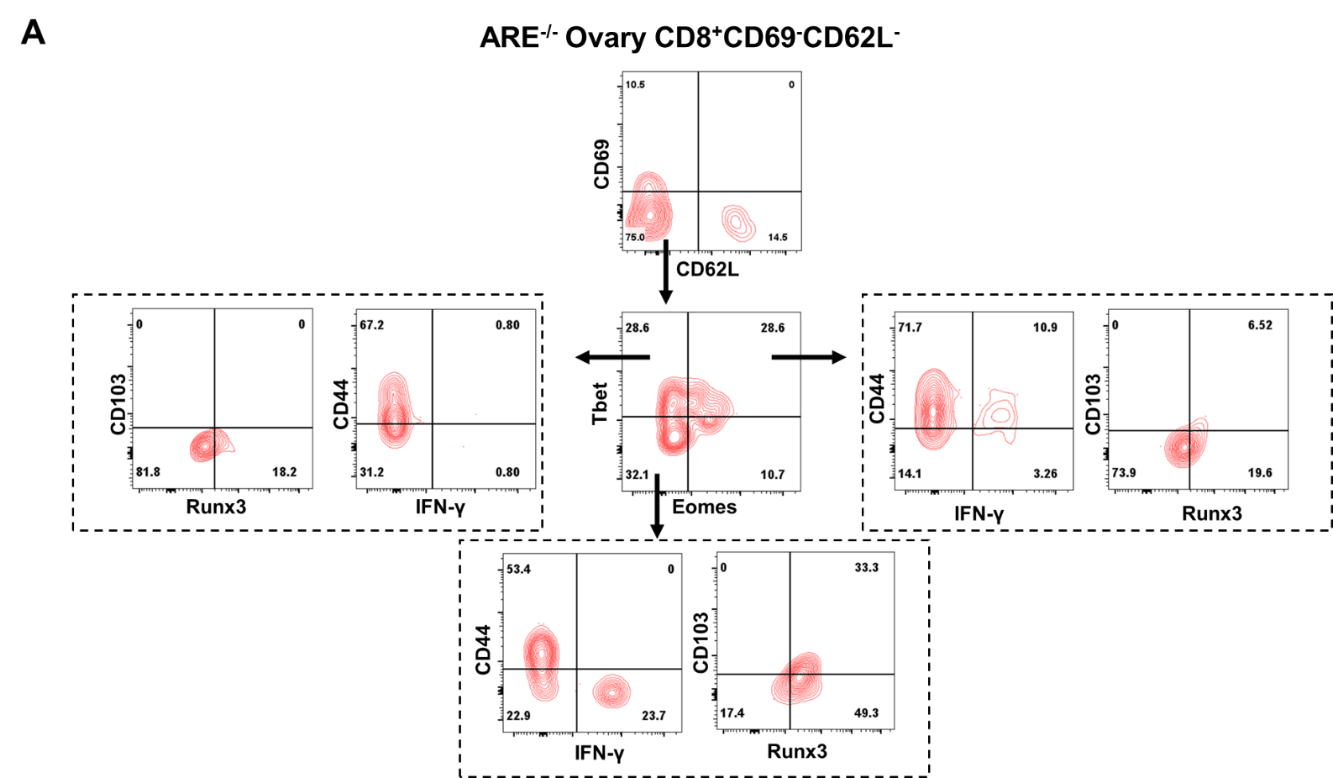


**Supplementary Figure 13. Analysis of CD8^+^CD69^-^CD62L^-^ T cells based on Tbet and Eomes, expression in ARE^-/-^ mouse ovary.** (**A**) Representative flow cytometry plots showing frequencies and phenotypes of CD69^-^CD62L^-^CD8^+^T cells based on Tbet, Eomes, CD44, IFN-γ, Runx3, and CD103 expression in ARE^-/-^ mouse ovary (n = 3). All experiments were performed two independent times, and ‘n’ denotes animals per group.


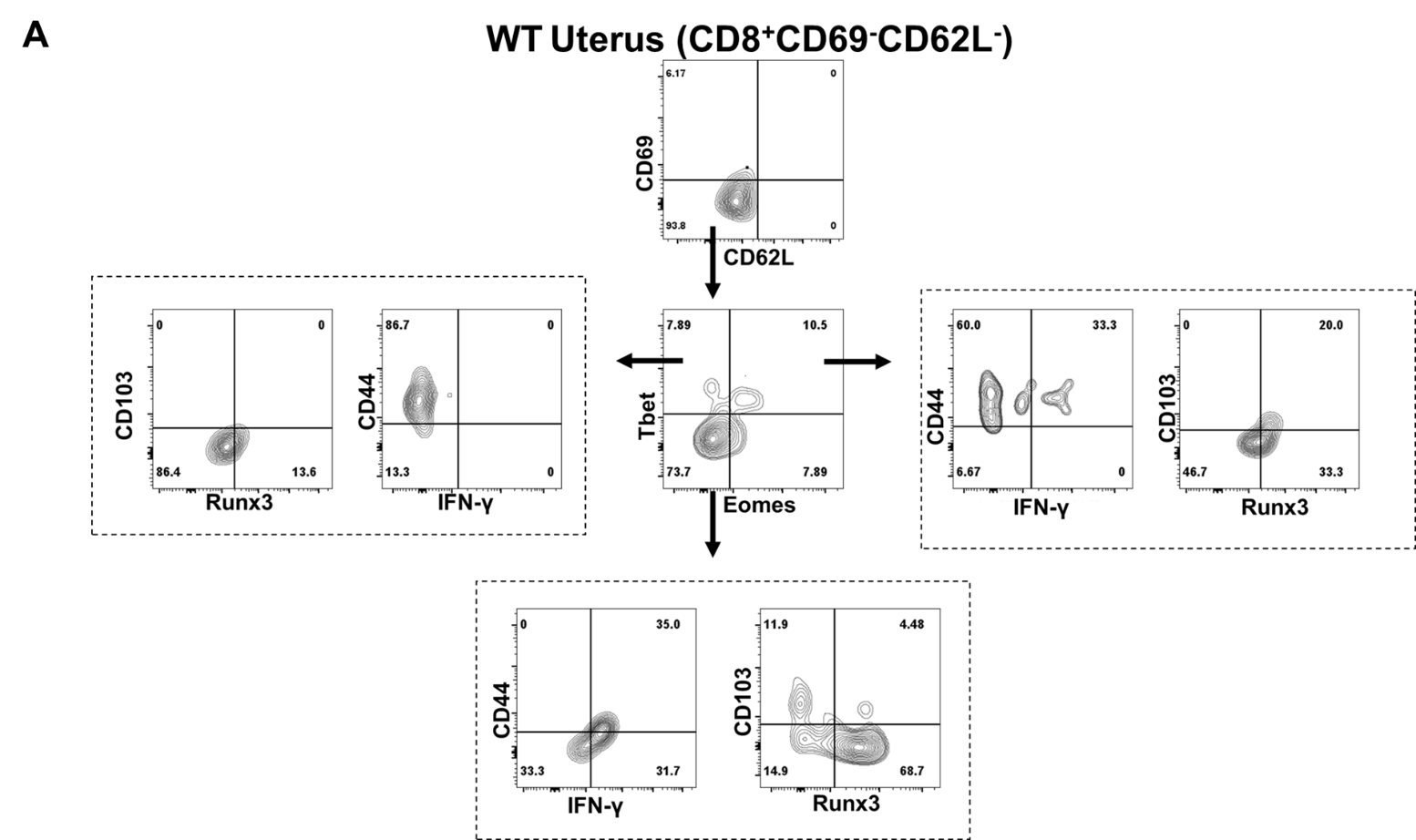


**Supplementary Figure 14. Analysis of CD8^+^CD69^-^CD62L^-^ T cells based on Tbet and Eomes, expression in WT mouse uterus.** (**A**) Representative flow cytometry plots showing frequencies and phenotypes of CD69^-^CD62L^-^CD8^+^T cells based on Tbet, Eomes, CD44, IFN-γ, Runx3, and CD103 expression in WT mouse uterus (n = 3). All experiments were performed two independent times, and ‘n’ denotes animals per group.

**
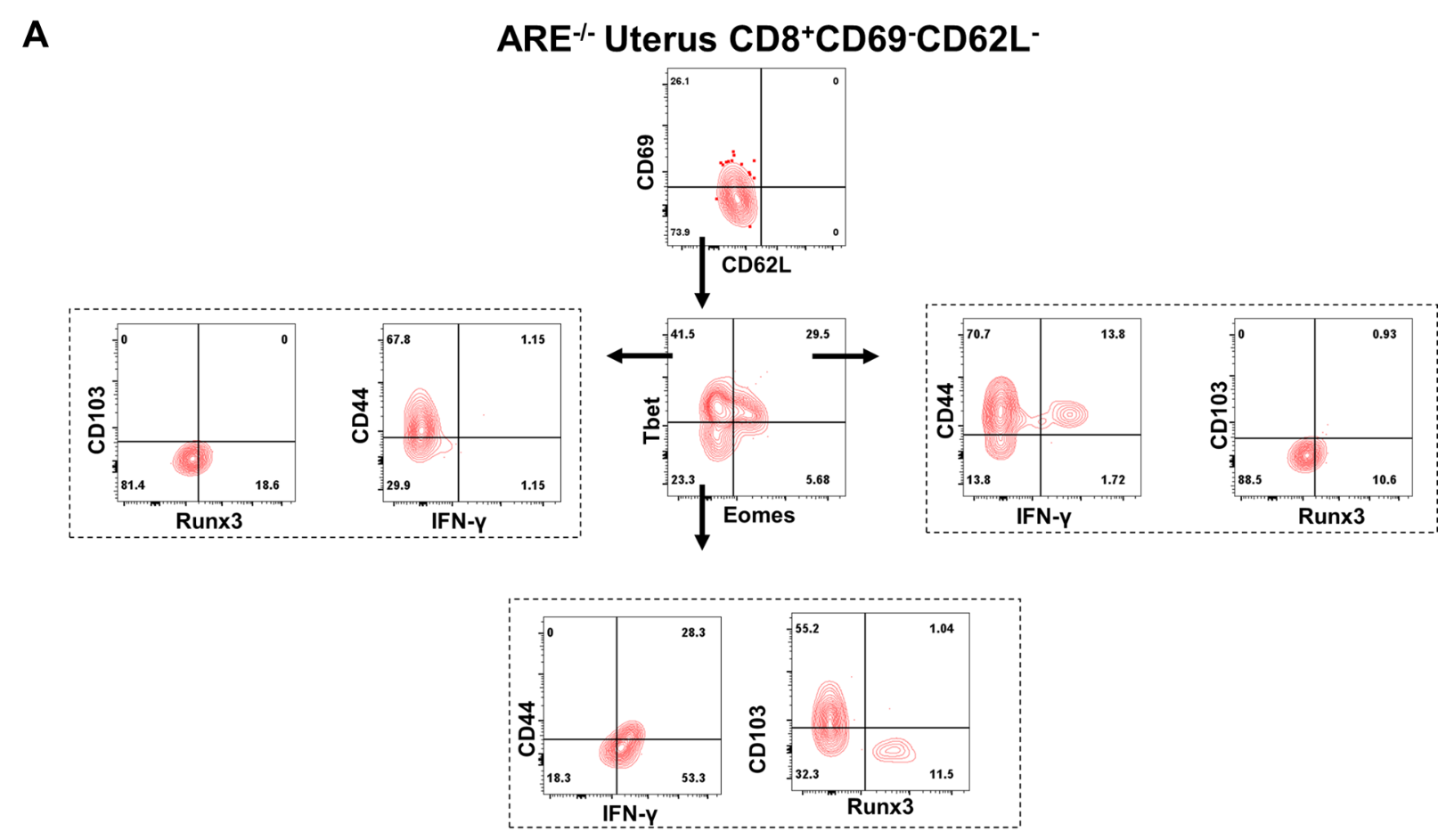
**

**Supplementary Figure 15. Analysis of CD8^+^CD69^-^CD62L^-^ T cells based on Tbet and Eomes, expression in ARE^-/-^ mouse uterus.** (**A**) Representative flow cytometry plots showing frequencies and phenotypes of CD69^-^CD62L^-^CD8^+^T cells based on Tbet, Eomes, CD44, IFN-γ, Runx3, and CD103 expression in ARE^-/-^ mouse uterus (n = 3). All experiments were performed two independent times, and ‘n’ denotes animals per group.

**
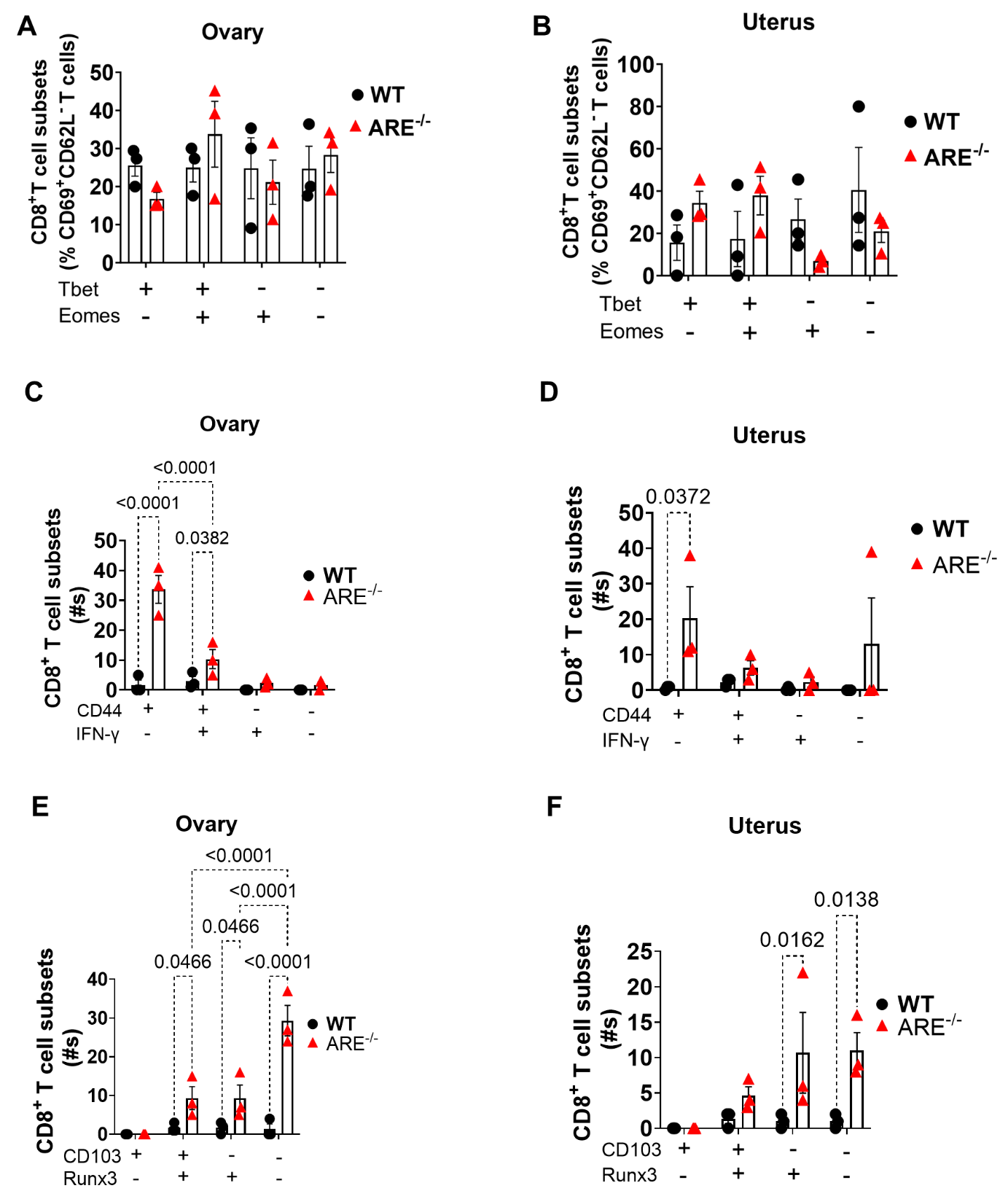
**

**Supplementary Figure 16. Analysis of CD69^+^CD62L^-^ CD8^+^T cell phenotypes in the ovary and uterus. (A-B)** There were no significant differences in the frequencies of Tbet and Eomes expression on CD69^+^CD62L^-^ CD8^+^T cells, in the ovary and uterus of WT and ARE^-/-^ mice (n = 3). (**C-D**) Gating on Tbet^+^Eomes^+^ subsets, absolute numbers of CD44^+^IFN-γ^-^ and CD44^+^IFN-γ^+^ CD8^+^T cell subsets increased in the ARE^-/-^ mouse ovary and uterus compared to the WT group (n = 3). (**E-F**) Gating on Tbet^+^Eomes^+^ subsets, CD103^-^Runx3^-^ subsets increased and were dominant in the ARE^-/-^ mouse ovary and uterus compared to WT cohorts (n = 3). All experiments were performed two independent times. Statistical significance, two-way ANOVA with Tukey’s multiple comparison test; data represent mean ± SEM, and ‘n’ denotes animals per group.


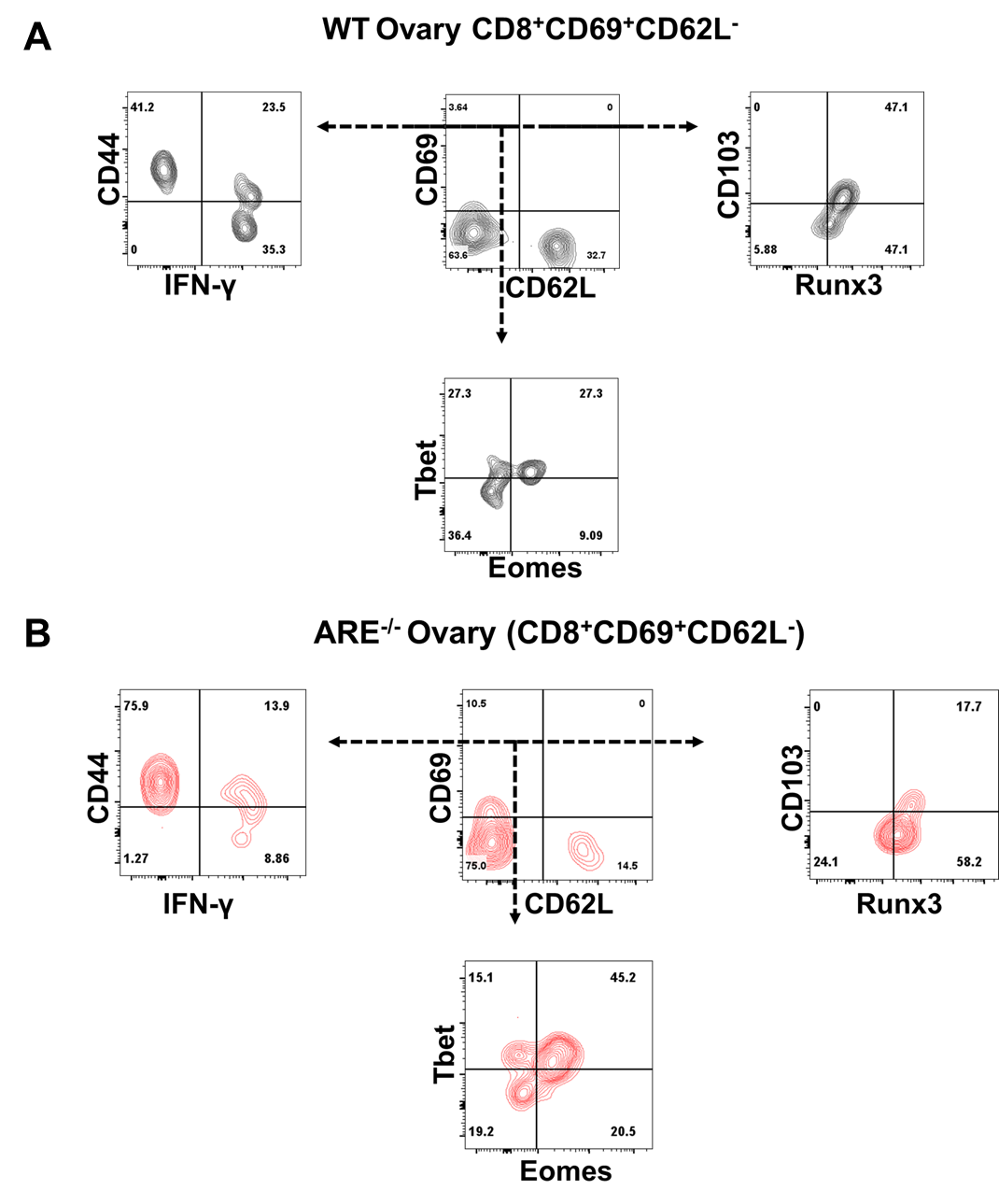


**Supplementary Figure 17. Analysis of CD8^+^CD69^-^CD62L^-^ T cell phenotypes in WT and ARE^-/-^ mouse ovary.** (**A-B**) Representative flow cytometry density plots showing frequencies and phenotypes of CD69^+^CD62L^-^CD8^+^T cells based on Tbet, Eomes, CD44, IFN-γ, Runx3, and CD103 expression in (**A**) WT and (**B**) ARE^-/-^ mouse ovary (n = 3). All experiments were performed two independent times, and ‘n’ denotes animals per group.

**
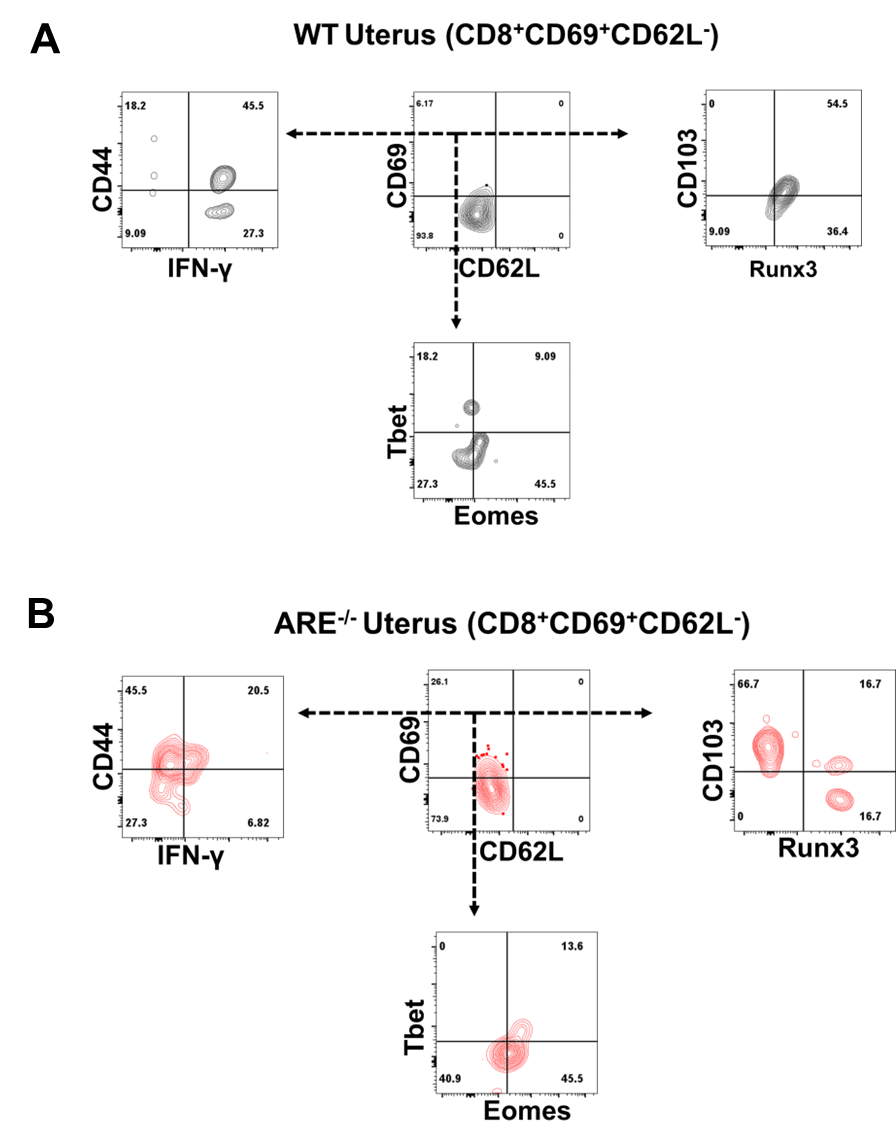
**

**Supplementary Figure 18. Analysis of CD8^+^CD69^-^CD62L^-^ T cell phenotypes in WT and ARE^-/-^ mouse uterus.** (**A-B**) Representative flow cytometry density plots showing frequencies and phenotypes of CD69^+^CD62L^-^CD8^+^T cells based on Tbet, Eomes, CD44, IFN-γ, Runx3, and CD103 expression in (**A**) WT and (**B**) ARE^-/-^ mouse uterus (n = 3). All experiments were performed two independent times, and ‘n’ denotes animals per group.

**
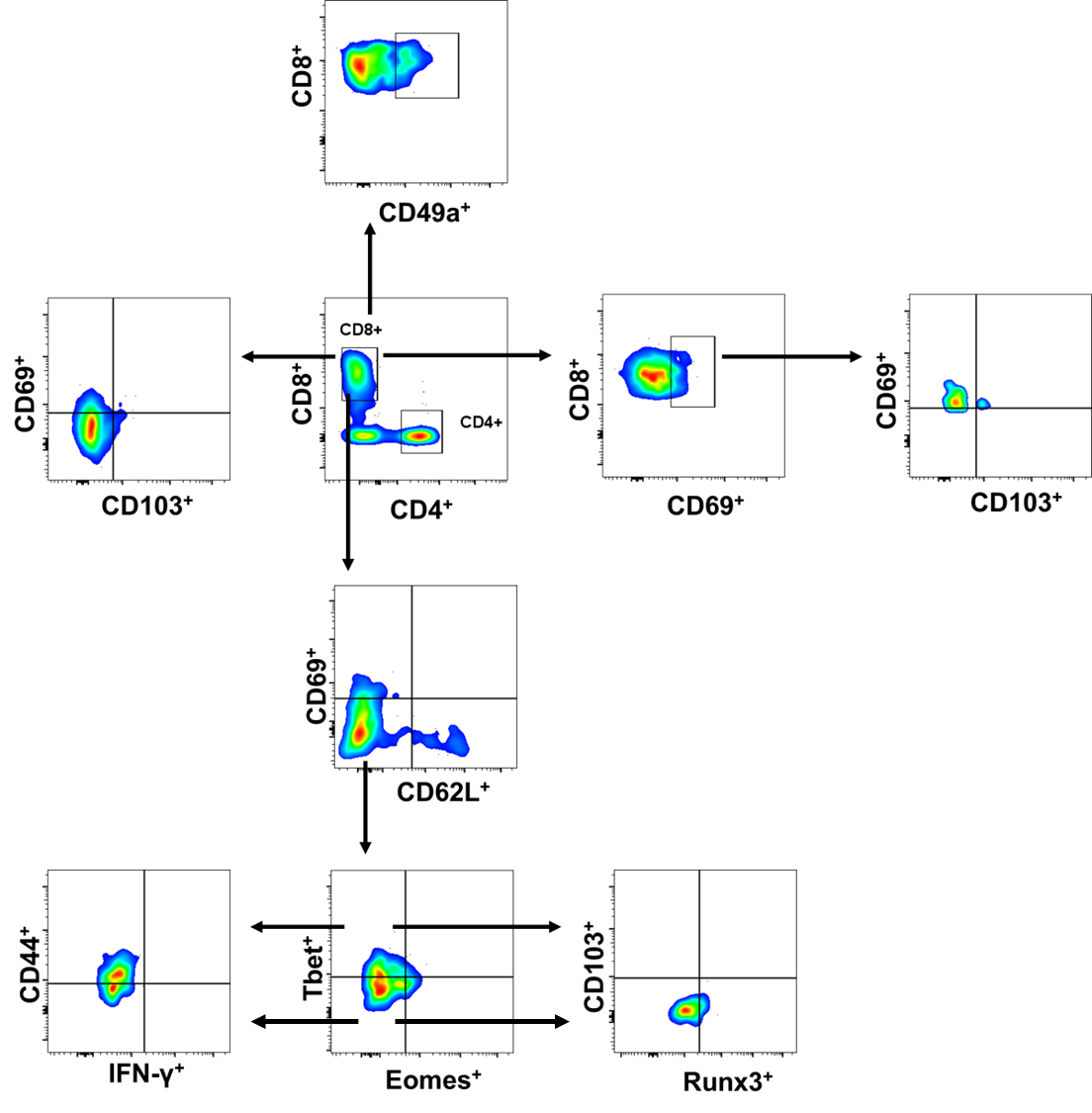
**

**Supplementary Figure 19**. Representative plots showing the gating strategy for T_RM_ and T_EM_ CD8^+^T cells in the ovary and uterus. Following exclusion of lysed cells (≤ 50K on FSC-A) and doublets (FSC-H vs FSC-A) and gating on live cells based on positive staining Zombie Aqua cells (Supplementary Figure 4); CD8^+^T cells were gated from CD3^+^T cells and CD49a and CD69 expression were assessed from CD8 positive cells. Subsequently, Tbet and Eomes expression were assessed on CD69^+/-^ and CD62L^-^ CD8^+^ cells, and CD103, Runx3, CD44 and IFN-γ assessed on Tbet^+/-^ Eomes^+/-^ CD8^+^T cells.

**Supplementary Table 5. Significantly upregulated genes encoding integrins identified by RNAseq in ARE^-/-^ mouse ovary.**

| GENE NAME | ENSEMBL ID | LRT adj  p-value | ARE^+/-^ vs WT  pairwise log2fc | ARE^-/-^ vs WT pairwise log2fc |
| --- | --- | --- | --- | --- |
| *Itgb7* | ENSMUSG00000001281 | 7.56E-19 | 1.784516338 | 2.970706268 |
| *Itga4* | ENSMUSG00000027009 | 2.60E-06 | 1.147899914 | 1.505717165 |
| *ItgaD* | ENSMUSG00000070369 | 5.67E-07 | 4.560485029 | 5.016893473 |
| *Itgal* | ENSMUSG00000030830 | 1.61E-16 | 1.553645021 | 2.478247558 |
| *Itgb2* | ENSMUSG00000000290 | 1.58E-06 | 1.937815357 | 2.11256813 |
| *Itga11* | ENSMUSG00000032243 | 0.966801092 | -0.028162724 | -0.127094391 |

**Supplementary Table 6. Significantly upregulated genes encoding integrins identified by RNAseq in ARE^-/-^ mouse uterus.**

| **GENE NAME** | **ENSEMBL ID** | **LRT adj**  **p-value** | **ARE^+/-^ vs WT**  **pairwise log2fc** | **ARE^-/-^ vs WT pairwise log2fc** |
| --- | --- | --- | --- | --- |
| ***Itgb7*** | ENSMUSG00000001281 | 2.14E-16 | 1.434592508 | 2.074646795 |
| ***Itga4*** | ENSMUSG00000027009 | 0.145642284 | 0.508562085 | 0.980517481 |
| ***ItgaD*** | ENSMUSG00000070369 | 0.705274614 | 0.329810139 | 0.957179967 |
| ***Itgal*** | ENSMUSG00000030830 | 0.006147724 | 1.115384346 | 1.489420721 |
| ***Itgb2*** | ENSMUSG00000000290 | 0.001255199 | 1.332017546 | 1.577231843 |
| ***Itga11*** | ENSMUSG00000032243 | 2.94E-07 | 2.115007255 | 2.547354381 |


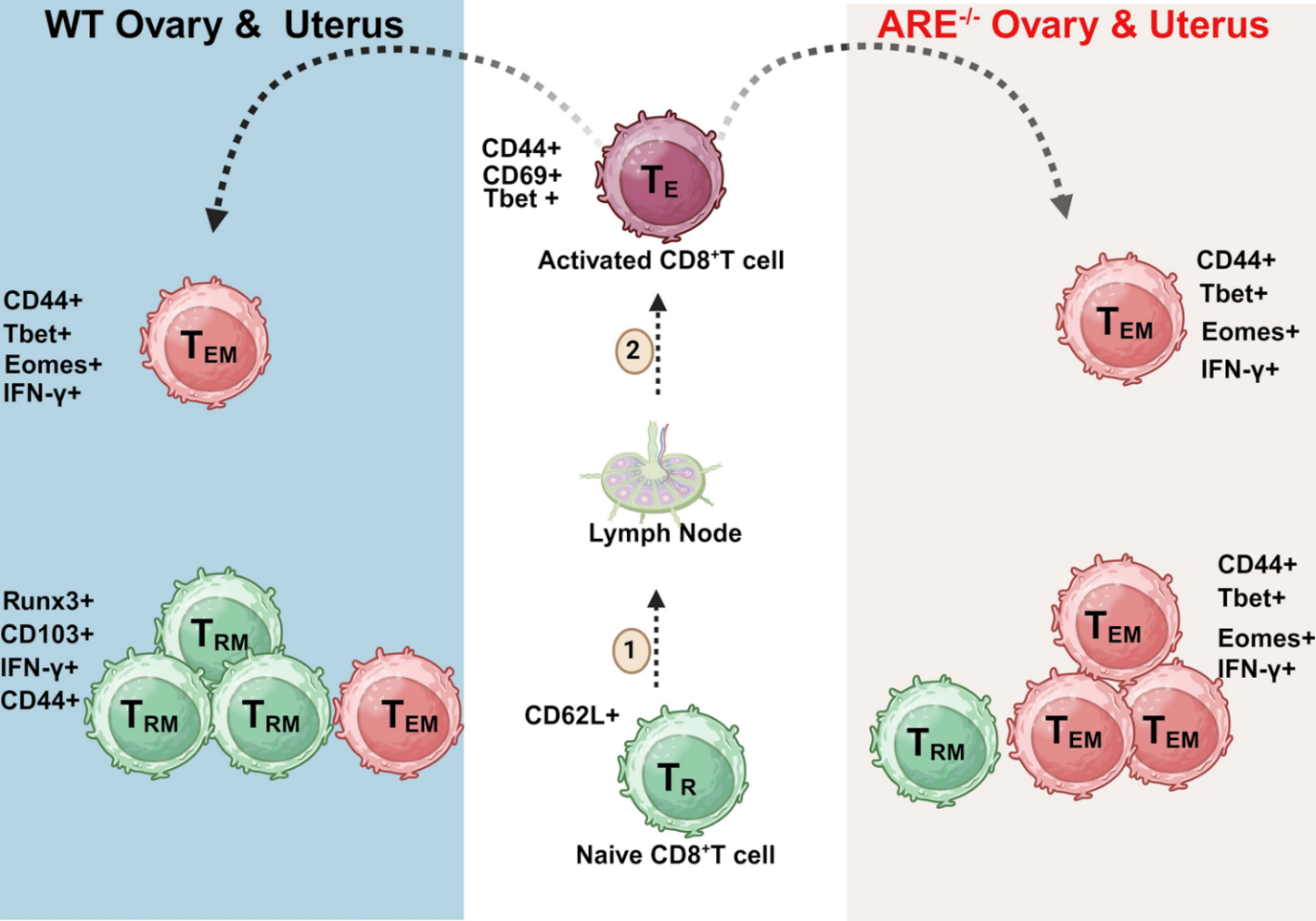


**Supplementary Figure 20. Schematic summarizing changes in the frequency of T_RM_ and T_EM_ CD8^+^T cells in WT and ARE^-/-^ ovary and uterus.** circled numbers = progression of differentiation; T_R_ = resting T cell; T_E_ = effector T cell; T_EM_ = effector memory T cell; T_RM_= resident memory T cell. Created using BioRender.com.

**
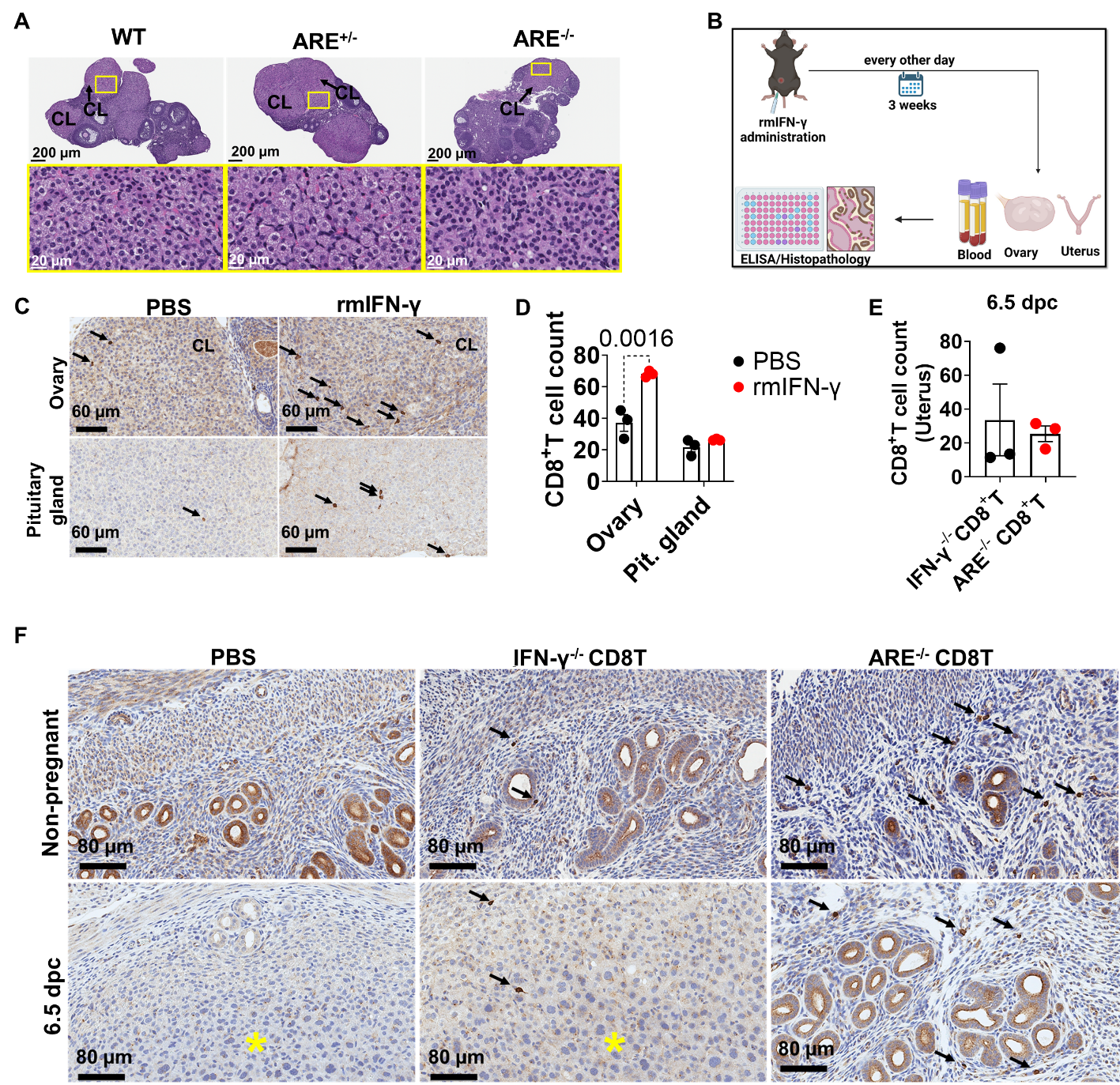
**

**Supplementary Figure 21. Elevated IFN-γ triggered CD8^+^T cell infiltration to the CL and anterior pituitary cells. (A)** Representative H&E images showing luteinized areas (yellow rectangles) in corpus luteum (CL) from 6.5 dpc females, (n= 4 mice/group). (**B**) Schema illustrating exogenous rmIFN-γ administration to WT females. Schematic created using BioRender.com. Representative images of CD8a positive cells (black arrows) in (**C**) ovaries and anterior pituitary in WT mice that received rmIFN-γ (n = 3 mice/group). (**D**) CD8^+^T cells significantly increased in the ovaries of mice that received rmIFN-γ (n = 3); (**E**) with no significant difference in the uterus. (**F**) Representative IHC images of uterine sections showing CD8^+^T cells (black arrows) in recipient *Rag1^-/-^* mice that received ARE^-/-^ or IFN-γ^-/-^ CD8^+^T cells or PBS; (n = 4 mice/group). All experiments were performed two independent times. Statistical significance, two-way ANOVA with Tukey’s test; data represent mean ± SEM, and ‘n’ denotes animals per group.

**Supplementary Materials and Method**

**Mouse genotyping**

All mice were genotyped at 3-4 weeks old and in some cases, tails were clipped when utilized for experiments for phenotypic confirmation by DNA extraction. ARE mice were genotyped as previously described (1). The primers used include WT ARE Reverse: 5’-ATTTAAAAATTCAAATAGTGCTGGC-3’, common ARE forward: 5’-GTCAACAACCCACAGGTCCA-3’ and ARE^-/-^ Reverse: 5’- CCGCGGTGGTACCATAACTT-3’. The PCR conditions for ARE^-/-^ primers were 94 °C for 2 min, 94 °C for 15 s, 60 °C for 15 s, 55 °C for 15 sec, repeat 35 cycles and for WT: 94 °C for 2 min, 94 °C for 15 s, 55 °C for 15 s, 68 °C for 15 sec, repeat 35 cycles.

**Estrous cycle phases**

The estrous cycle (known as the reproductive cycle in humans) is broadly subdivided into four phases: proestrus, estrus, metestrus, and diestrus (in order of progression). Proestrus (preovulatory stage) corresponds to the human follicular phase of the menstrual cycle and is dominated by elevated levels of estradiol (E2) with rising progesterone (P4) levels (2). Estrus corresponds to the peri-ovulation and ovulation periods, and P4 levels rapidly rises but gradually declines towards the end of the cycle. If mating does not occur, the cycle will continue toward the metestrus stage (3, 4). Metestrus (post-ovulation) is equivalent to the human luteal phase, characterized by elevated levels of both P4 and E2 with a gradual decline to basal levels. Rodents do not have a fully functional corpus luteum (CL) at this stage until mating occurs (5). The diestrus phase is the longest phase of the estrous cycle that corresponds to the follicular phase in humans, and it is a stage characterized by relatively low hormone concentrations (6, 7). A gradual rise of E2 occurs during this phase, while P4 concentration remains low (6). The diestrus phase is triggered by CL regression and occurs after metestrus (the period of follicle selection due to decreasing follicle stimulating hormone (FSH) and rising E2 from antral follicles (8). The CL is a transient endocrine gland formed by the ruptured follicle after ovulation (5). Upon ovulation, the oocyte with cumulus granulosa cells is released from the mature follicle and P4 is mainly produced in the CL after ovulation (5).

In this study, proestrus was defined by the dominant presence of nucleated epithelial cells and interspersed leukocytic and cornified epithelial cells (9, 10). Estrus was characterized by the dominant presence of anucleated cornified cells, often appearing in clusters (9, 10). The metestrus stage was defined as the stage characterized by combinations of leukocytic cells nucleated epithelial cells, and cornified epithelial cells (9, 10). The diestrus stage was defined as the stage characterized by the dominant presence of leukocytic cells (9, 10).

**Flow cytometry processing and staining**

Mouse antibodies for surface and intracellular markers are listed in Table S1. Compensation was calculated using single color controls and UltraComp eBeads (Invitrogen, ThermoFisher) and Zombie Aqua (Biolegend) was for live/dead discrimination. Gating analysis was done using single stained cell controls, unstained controls and florescence minus one (FMO) controls (11).

**Supplementary Table 7. Anti-mouse monoclonal antibodies for flow cytometry staining**

| Antibody | Fluorophore | Clone | Company |
| --- | --- | --- | --- |
| CD45 | Alexa Fluor-700 | 30-F11 | Biolegend |
| CD3 | PE/dazzle594 | 17A2 | Biolegend |
| CD4 | APC-eFluor780 | RM4-5 | Invitrogen, ThermoFisher Scientific |
| CD8a | FITC | 53-6.7 | Biolegend |
| CD8a | PE-Cy7 | 53-6.7 | Biolegend |
| NK1.1 | Brilliant Violet 711 | PK136 | Biolegend |
| CD178 | PE | MFL3 | Biolegend |
| IFN-γ | PerCP Cy5.5 | XMG1.2 | Invitrogen eBioscience |
| CD44 | PE | IM7 | Biolegend |
| CD44 | BUV395 | IM7 | BD Bioscience |
| CD62L | PerCPCy5.5 | MEL-14 | Biolegend |
| CD62L | PE-Cy5 | MEL-14 | Biolegend |
| Zombie | Aqua |  | Biolegend |
| CD69 | PE-Cy7 | H1.2F3 | eBioscience |
| CD49a | BUV661 | HMα1 | BD Bioscience |
| Tim-3 | PE | B8.2C12 | BD Bioscience |
| Lag-3 | BUV805 | C9B7W | BD Bioscience |
| CD103 | BUV615 | 2E7 | BD Bioscience |
| Tbet | APC | 4B10 | ThermoFisher |
| Eomes | BV421 | W17001A | BD Bioscience |
| Eomes | PE | W17001A | BD Bioscience |
| Runx3 | BV421 | R3-5G4 | BD Bioscience |

**Cytokine measurements**

Plasma samples from 4–6-month-old female mice per genotype were collected from experimental animals in plasma-separator tubes, processed within one hour of collection, and subsequently stored at -80 ^o^C until needed. Cytokine assays were assayed on plasma samples using custom murine multiplex Meso-Scale Discovery (MSD) kits, which measured analytes in a 10-plex format (IFN-γ, IL-1β, IL-6, IL-10, IL-12p70, IL-27p28/IL-30, IP-10, MIP-1β, MIP-2, and TNF-α; Catalog #. K15069M-2 and K15069L-2). Samples were tested in duplicate and read on MSD QuickPlex SQ 120MM imager. Raw signals generated by the instrument were analyzed using Discovery Workbench 4.0 Software, followed by statistical analysis in GraphPad Prism (v.10.1) for Windows (GraphPad, San Diego, CA). Assays performed in duplicate were averaged and multiplied by the dilution factor for analysis. All cytokine values were reported in units of pg/mL, and data were presented as mean ± SEM.

**Quantitative Real Time-Polymerase Chain Reaction (qRT-PCR)**

RNA and DNA quality were analyzed using Nanodrop one microvolume UV-Vis spectrophotometer (Fisher Scientific). All validated gene target TaqMan probes (Table S2) were processed using TaqMan universal Master Mix II no UNG (Invitrogen) according to manufacturer’s instructions.

**Supplementary Table 8. Anti-mouse Taqman probes and corresponding assay IDs**

| Taqman probe | Assay ID |
| --- | --- |
| *Ifng* | Mm01168134_m1 |
| *Hprt1* | Mm03024075_m1 |

Quantitative RT-PCR was performed on a Light Cycler 480 (Roche) system.

**Supplementary References**

1. Valencia JC, Erwin-Cohen RA, Clavijo PE, Allen C, Sanford ME, Day CP, et al. Myeloid-Derived Suppressive Cell Expansion Promotes Melanoma Growth and Autoimmunity by Inhibiting CD40/IL27 Regulation in Macrophages. Cancer Res. 2021;81(23):5977-90.

2. Wood GA, Fata JE, Watson KL, Khokha R. Circulating hormones and estrous stage predict cellular and stromal remodeling in murine uterus. Reproduction. 2007;133(5):1035-44.

3. Cora MC, Kooistra L, Travlos G. Vaginal Cytology of the Laboratory Rat and Mouse: Review and Criteria for the Staging of the Estrous Cycle Using Stained Vaginal Smears. Toxicol Pathol. 2015;43(6):776-93.

4. Hubscher CH, Brooks DL, Johnson JR. A quantitative method for assessing stages of the rat estrous cycle. Biotech Histochem. 2005;80(2):79-87.

5. Hennebold JD. Corpus Luteum. In: Skinner MK, editor. Encyclopedia of Reproduction (Second Edition). Oxford: Academic Press; 2018. p. 99-105.

6. Becker JB, Arnold AP, Berkley KJ, Blaustein JD, Eckel LA, Hampson E, et al. Strategies and methods for research on sex differences in brain and behavior. Endocrinol. 2005;146(4):1650-73.

7. Bertolin K, Murphy BD. Reproductive Tract Changes During the Mouse Estrous Cycle. In: B. Anne Croy ATY, Francesco J. DeMayo, S. Lee Adamson, editor. The Guide to Investigation of Mouse Pregnancy: Academy Press; 2014. p. 85-94.

8. Zeleznik AJ. The physiology of follicle selection. Reprod Biol Endocrinol. 2004;2:31.

9. Bafor EE, Ukpebor F, Elvis-Offiah U, Uchendu A, Omoruyi O, Omogiade GU. Justicia flava Leaves Exert Mild Estrogenic Activity in Mouse Models of Uterotrophic and Reproductive Cycle Investigations. J Med Food. 2020;23(4):395-408.

10. Diener KR, Robertson SA, Hayball JD, Lousberg EL. Multi-parameter flow cytometric analysis of uterine immune cell fluctuations over the murine estrous cycle. J Reprod Immunol. 2016;113:61-7.

11. Selliah N, Eck S, Green C, Oldaker T, Stewart J, Vitaliti A, Litwin V. Flow Cytometry Method Validation Protocols. Curr Protoc Cytom. 2019;87(1):e53.
